# Maritime traffic congestion identification and ship trajectory prediction using temporal graph convolutional networks

**DOI:** 10.1371/journal.pone.0342781

**Published:** 2026-03-09

**Authors:** Weiping Zhou, Weiming Zhang, Shihu Sun, Yuquan Zhang

**Affiliations:** 1 Jiangxi Polytechnic University, Jiujiang, China; 2 Hebei Jiaotong Vocational and Technical College, Shijiazhuang, China; National University of Defense Technology, CHINA

## Abstract

With the rapid growth of global maritime trade, the efficient and safe management of maritime traffic has become increasingly critical. This study proposes a comprehensive framework for ship trajectory prediction and maritime traffic congestion identification based on Automatic Identification System (AIS) data. We integrate spatiotemporal analysis with deep learning techniques, specifically combining Graph Convolutional Networks (GCN) and Gated Recurrent Units (GRU) to form a Temporal Graph Convolutional Network (T-GCN) model. This model effectively captures both spatial dependencies among ships and temporal dynamics in traffic flow. Furthermore, we introduce a congestion measurement indicator based on the Speed Performance Index (SPI) to quantify and identify congestion levels in maritime routes. The proposed method not only enhances the accuracy of ship trajectory prediction but also enables proactive congestion warnings, contributing to improved maritime safety and operational efficiency. Experimental results demonstrate the effectiveness of our approach in real-world scenarios.

## 1. Introduction

Maritime transportation plays a vital role in global trade and economic development, accounting for over 80% of the world’s trade volume [1]. With the increasing density of ship traffic, ensuring navigational safety and optimizing traffic flow have become significant challenges [2]. This exponential growth has not only amplified the risks of navigational accidents (such as collisions, groundings, and oil spills) but also led to frequent traffic congestion in port approaches, narrow waterways, and busy coastal areas [3]. As a result, ensuring navigational safety, reducing voyage delays, optimizing traffic flow efficiency, and minimizing the environmental impact of maritime activities have become pressing and interconnected challenges for maritime authorities, port operators, and shipping companies worldwide.

The Automatic Identification System (AIS), a mandatory maritime surveillance technology for vessels over a certain tonnage, has emerged as a cornerstone data source for addressing these challenges. By continuously transmitting and receiving real – time spatiotemporal data—including ship position (latitude/longitude), speed over ground (SOG), course over ground (COG), heading, vessel identity (MMSI/IMO number), dimensions, draft, and cargo type—AIS enables comprehensive monitoring and analysis of ship movements across global maritime spaces [4]. However, AIS data often contains noise, missing values, and inconsistencies, necessitating robust preprocessing and modelling techniques [5].

Traditional methods for trajectory prediction and congestion identification often rely on statistical or simple machine learning models, which may fail to capture the complex spatiotemporal dependencies inherent in maritime traffic. Recent advances in deep learning [6], particularly graph-based and sequence-based models, offer promising avenues for addressing these limitations. This study leverages a hybrid deep learning framework—Temporal Graph Convolutional Network(T-GCN)—to model both the structural relationships among ships and the temporal evolution of traffic states. By integrating trajectory prediction with congestion identification, we aim to provide a holistic solution for maritime traffic management.

## 2. Literature review

The prediction of port congestion is a critical issue within intelligent shipping systems and maritime management [7]. In recent years, with the widespread availability of AIS data and improvements in computational power, data-driven prediction methods—particularly those based on machine learning and deep learning models—have gradually become mainstream in research. This section reviews relevant literature from four perspectives: traditional prediction methods, machine learning and deep learning models, Traffic congestion, and limitations and challenges of existing research.

### 2.1 Ship prediction methods

Target ship motion prediction has emerged as an indispensable technical cornerstone for ensuring maritime navigational safety, optimizing route planning, and realizing efficient coordination in dense traffic, given the unique challenges inherent in forecasting target ship movements—challenges that distinguish it from the motion prediction of the “own ship” (the vessel conducting the prediction). Unlike the motion prediction of own ship, the movement of target ships is subject to multiple disturbances from external environmental factors (e.g., wind, waves, and currents) as well as the behavior of surrounding ships, leading to significant motion uncertainty [8]. Moreover, the precise dynamic model of a target ship is often difficult to obtain, and its navigation decisions are heavily influenced by crew intentions, resulting in complex and variable behaviour patterns [9]. Therefore, motion prediction has become a primary approach to address the uncertainty in target ship motion.

Currently, target ship motion prediction methods can be broadly categorised into two types: trajectory-based motion prediction and interaction-aware motion prediction.

Trajectory-based motion prediction primarily analyses historical trajectory data of the target ship to uncover motion patterns and evolutionary trends, thereby forecasting its future state. This approach does not rely on direct communication between ships, making it highly practical. Common techniques include Bayesian networks [10], neural networks [6], adaptive filtering algorithms (e.g., Kalman filters and their variants) [11], and probabilistic obstacle methods [12]. [13] improved the traditional Markov model by integrating the temporal and spatial continuity of trajectories, significantly enhancing the accuracy of local trajectory forecasting. For long-term motion prediction, [14] incorporated safety distance constraints into a linear prediction model to mitigate the impact of motion uncertainty. However, due to the complex influence of crew intentions, weather, sea conditions, and other factors, trajectory-based methods still face limitations in prediction accuracy and robustness. To address this, [15] proposed a prediction framework that accounts for motion uncertainty by combining route priors with Gaussian Process (GP) modelling, enabling probabilistic prediction of both destination and future trajectory. [16] further developed polynomial logistic regression and Gaussian process regression models, providing confidence interval estimates for ship trajectories, thereby enhancing the interpretability and safety of predictions. The ability of Gaussian processes to quantify prediction uncertainty makes them particularly promising for collision avoidance decision-making, contributing to safer and more intelligent navigation planning.

Interaction-aware motion prediction, on the other hand, emphasizes active cooperation and intent sharing between ships. This method enables the own ship to accurately understand the target ship’s trajectory by directly exchanging navigational intent (e.g., destination port, planned route, speed adjustments) through communication channels such as broadcast systems or Very High Frequency (VHF) radio [17]. Additionally, distributed optimization algorithms—such as distributed stochastic search algorithms [18], the Alternating Direction Method of Multipliers (ADMM) [19], and Distributed Model Predictive Control (DMPC) [20]—can be employed to coordinate navigation intentions among multiple ships, enabling cooperative collision avoidance and traffic optimization. While theoretically capable of significantly improving prediction accuracy and navigational safety, the practical implementation of this approach depends on highly reliable communication infrastructure, standardized intent-exchange protocols, and a high degree of inter-ship cooperation. These requirements pose significant challenges in open waters and international navigation, limiting widespread deployment.

In summary, AIS-based trajectory prediction methods strike an optimal balance between practicality and performance in target ship motion forecasting. By leveraging widely available and mandatory AIS data, these methods offer a robust, universally deployable solution that operates independently of inter-ship communication and infrastructure support. They extract motion patterns—such as ship-type-specific speed profiles and route preferences—directly from historical trajectories, enabling reliable predictions even in remote or underdeveloped maritime regions where VDES or satellite coverage is limited. This autonomy eliminates dependence on unstable communication systems, bypasses the need for standardized intent-sharing protocols, and overcomes barriers to cross-fleet cooperation, making AIS-driven approaches uniquely scalable and effective across diverse operational contexts.

### 2.2 Machine learning and deep learning models

With the rapid advancement of big data analytics and machine learning technologies, data-driven approaches have become increasingly prevalent in maritime traffic and trajectory prediction [21]. Traditional machine learning models such as Support Vector Regression (SVR) have been widely explored for their ability to handle nonlinear sequential patterns in ship motion data. For instance, [22] proposed an Adaptive Differential Evolution(ACDE)–SVR model, where an Adaptive Differential Evolution algorithm optimizes key SVR parameters using AIS data, achieving higher prediction accuracy than standard SVR methods. However, due to its reliance on offline training, this approach lacks adaptability to real-time changes in vessel behavior or environmental conditions, limiting its applicability in dynamic, online navigation scenarios.

To better capture temporal dynamics, deep learning models—particularly Recurrent Neural Networks (RNNs)—have emerged as powerful tools for sequence modelling in trajectory prediction [23]. [24] formulated ship trajectory forecasting as a sequence-to-sequence task and developed an LSTM-based model that effectively captures long-term temporal dependencies, significantly improving prediction performance. Building on this, [25] introduced a bidirectional LSTM–RNN framework that leverages both past and future context (within the observed window) to model complex vessel motion patterns. This architecture enhances the representation of dynamic ship behavior and supports online learning, enabling real-time updates and predictions—an essential feature for practical deployment in intelligent maritime navigation systems.

Beyond recurrent architectures, other deep neural networks have also demonstrated strong potential. [26] designed a real-time motion trajectory prediction system using Artificial Neural Networks (ANNs), showing high responsiveness and adaptability in dynamic target tracking tasks. These studies collectively highlight the superiority of deep learning models in capturing nonlinear dynamics and complex spatiotemporal patterns, significantly outperforming traditional statistical and machine learning methods in accuracy and robustness.

In the broader field of intelligent systems, advanced models and techniques have provided valuable insights for maritime trajectory prediction. For example, the Kolmogorov-Arnold network with flexibly selected activation functions has demonstrated strong universal modeling capabilities in predictive tasks involving multiple factors, offering inspiration for handling the complex interplay of ship motion parameters and environmental factors [27]. The adaptive fused domain-cycling variational generative adversarial network addresses data scarcity challenges through multi-domain feature modeling and high-quality data fusion, which is highly relevant to scenarios where AIS data may be limited or of poor quality [28]. Additionally, the M2BIST-SPNet [29] for RUL prediction integrates advanced spatiotemporal feature extraction mechanisms, emphasizing the importance of multi-scale and multi-parameter fusion—principles that can be extended to ship trajectory prediction to enhance long-term prediction accuracy.

Nevertheless, a critical limitation of most existing approaches is their focus on modelling individual vessel trajectories in isolation, treating ships as independent agents. This simplification overlooks crucial inter-vessel interactions—such as collision avoidance maneuvers, convoy-like behavior, and traffic flow coordination—as well as spatial constraints imposed by port layouts, narrow channels, and anchorage zones. As maritime traffic grows denser, especially in busy ports and coastal waters, such contextual factors become increasingly influential in shaping vessel motion. Therefore, future research is expected to shift toward integrated, context-aware prediction frameworks that incorporate Graph Neural Networks (GNNs) to model spatial relationships among vessels, attention mechanisms to identify salient interactions, and multimodal data fusion (e.g., AIS, electronic nautical charts, weather conditions, and tidal information) to enhance environmental awareness. Such holistic systems will enable more accurate, robust, and interpretable trajectory predictions, paving the way for next-generation intelligent maritime traffic management and autonomous navigation solutions.

### 2.3 Traffic congestion

Traffic congestion identification and prediction are essential for intelligent transportation systems (ITS), enabling improved traffic management, enhanced operational efficiency, and better decision-making [30]. Congestion is typically classified as either incidental or recurring. Incidental congestion arises from unexpected events—such as accidents or breakdowns—and is detected through abrupt changes in traffic parameters like speed drops or flow disruptions. Timely identification allows rapid response, helping to limit the spread of delays [31]. In contrast, recurring congestion stems from chronic imbalances between demand and capacity, occurring regularly at specific locations and times (e.g., peak hours near port entrances). Identifying such patterns requires spatiotemporal analysis to reveal persistent bottlenecks, supporting long-term planning and proactive mitigation strategies. To transform fragmented detection data into meaningful congestion zones, clustering techniques—including density-based, partitioning, and grid-based methods—are widely used to aggregate discrete observations into continuous spatiotemporal regions [32].

Congestion prediction, in contrast, aims to forecast future traffic states, offering proactive insights into congestion onset, duration, intensity, and propagation. It plays a critical role in understanding causality, designing control strategies (e.g., speed advisories, traffic routing), and enabling preventive actions. Applications extend to dynamic route planning, fleet scheduling, and infrastructure investment analysis. At its core, congestion prediction involves modelling and extrapolating key traffic state variables—such as vessel/vehicle speed, traffic density, occupancy, travel time, and composite congestion indices—over a future time horizon [33]. Early approaches relied heavily on statistical and time-series models, with the Autoregressive Integrated Moving Average (ARIMA) being one of the most widely used due to its effectiveness in modelling linear, stationary traffic patterns. However, ARIMA struggles with nonlinear dynamics, non-stationarity, and external influences (e.g., weather, tides), limiting its accuracy in complex real-world scenarios. Bayesian networks have also been applied to model probabilistic dependencies among traffic events and environmental factors, demonstrating strength in handling uncertainty and causal reasoning [34]. Additionally, Kalman filtering has been utilized for real-time state estimation and short-term pattern tracking, particularly in systems with noisy observations [35].

With the proliferation of large-scale, high-resolution transportation data, data-driven machine learning methods have increasingly outperformed traditional models in capturing complex, nonlinear traffic dynamics [36]. Linear regression models, including multiple linear regression, have been used to predict daily congestion trends by incorporating historical traffic and weather data [37], but they often fail to capture high-dimensional, non-stationary, and spatiotemporally correlated patterns [38]. As a result, ensemble learning methods such as Random Forest (RF) and Gradient Boosting Machines (GBM) have gained popularity due to their robustness, ability to handle heterogeneous features, and relative interpretability [39]. Shallow neural networks, including Backpropagation Neural Networks (BPNN), Feedforward Neural Networks (FNN), and Hopfield networks, have also been applied to short-term traffic state forecasting [40], but they often suffer from limited representational capacity and overfitting when dealing with large-scale, high-dimensional datasets.

Recent research in intelligent transportation has witnessed significant innovations that can inform maritime congestion prediction. For example, the roadside LiDAR placement optimization method based on chance-constrained stochastic simulation optimization improves cooperative traffic detection accuracy through rational sensor layout, providing a reference for the optimal deployment of maritime monitoring equipment (such as AIS base stations and radar) to enhance congestion data collection quality [41]. The multimodal arc detection technique for railway systems with limited data combines audio-visual semantic information to address small-sample challenges, which can be adapted to maritime scenarios where certain congestion-related data (e.g., in remote waters) is scarce [42]. The virtual-real fusion framework for intelligent 3D traffic accident reconstruction integrates simulation and computer vision techniques to generate high-fidelity traffic scenes, offering a new approach for constructing maritime congestion simulation environments to train and validate prediction models [43]. These advances in intelligent transportation underscore the importance of multi-source data fusion, simulation-driven modeling, and small-sample learning—directions that are equally critical for advancing maritime traffic congestion prediction.

In maritime contexts, Graph Neural Networks (GNNs) are particularly promising, as they can explicitly model vessel interactions and navigational constraints (e.g., channel topology, port layout). The integration of such techniques with the aforementioned intelligent transportation innovations is expected to further improve the accuracy and robustness of maritime congestion prediction.

### 2.4 Outline of the paper

Despite significant advances in prediction accuracy and model sophistication, maritime congestion forecasting still faces several critical challenges. First, data quality issues—such as noise, missing values, and inconsistencies in AIS data—combined with the absence of a standardized definition of “congestion” across ports, undermine the robustness and comparability of existing models. Second, the limited interpretability of deep learning models, often perceived as “black boxes,” compromises transparency and trust, hindering their adoption in safety-critical maritime operations and regulatory decision-making. Third, most current research focuses on short-term forecasting (ranging from hours to one day), leaving medium- and long-term congestion trends—crucial for strategic port planning, infrastructure investment, and policy formulation—largely unaddressed. Moreover, a pronounced regional and scale bias exists, as models are predominantly trained on data from large, well-instrumented ports, resulting in poor generalization to small- and medium-sized or developing-region ports with less comprehensive monitoring infrastructure. Finally, although initial efforts have integrated insights from environmental science (e.g., carbon emissions monitoring) and economics, broader interdisciplinary integration remains fragmented, limiting the development of holistic, sustainable port management frameworks. Overcoming these challenges is essential to enhance both the scientific validity and real-world applicability of maritime traffic prediction systems.

In conclusion, research on port congestion prediction is shifting from traditional statistical methods to more complex deep learning and hybrid models, with particular emphasis on spatiotemporal dependency modeling and multi-source information fusion. However, challenges such as data inconsistency, poor model generalization, and lack of interpretability remain major obstacles in the field. Future research should focus more on interdisciplinary collaboration, the development of long-term prediction mechanisms, and systematic validation in real-world environments.

The main contributions of this work are threefold. First, we propose a structured pipeline for AIS data processing, systematically addressing data cleaning, missing value interpolation, and ship trajectory reconstruction to provide a high-quality data foundation for subsequent analysis. Second, we develop a (T-GCN) that integrates Graph Convolutional Networks GCN with GRU to effectively capture spatiotemporal dependencies in ship movements, thereby improving trajectory prediction accuracy. Third, we define a novel congestion index based on ship speed performance, enabling automatic detection and early warning of maritime traffic congestion, thus providing a quantitative basis for maritime traffic management.

The remainder of this paper is organized as follows: Section 2 reviews related work; Section 3 details the methodology; Section 4 presents a case study; Section 5 discusses the results; and Section 6 concludes the study.

## 3. Methodology

### 3.1 Overall framework architecture

**Fig 1:**
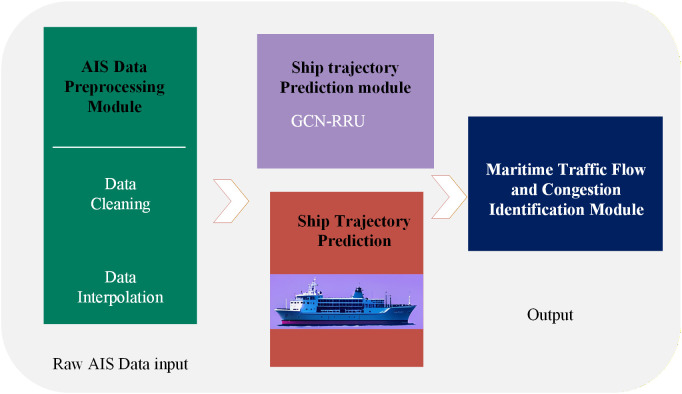
The flowchart of the provided algorithm.

The proposed framework for ship trajectory prediction and maritime traffic congestion identification consists of four integrated components, forming an end-to-end analytical pipeline capable of processing raw AIS data and generating both trajectory forecasts and congestion warnings. As illustrated in Fig 1, the overall architecture comprises:

(1) an AIS Data Preprocessing Module,
(2) a Trajectory Reconstruction and Interpolation Module,
(3) a Ship Trajectory Prediction Module based on a T-GCN, and
(4) a Maritime Traffic Flow and Congestion Identification Module.

Raw AIS data are first cleansed and normalized in the preprocessing module, where erroneous, duplicate, and physically implausible records (e.g., outlier speeds or positions) are filtered out. The cleaned data then undergo trajectory reconstruction and interpolation to form complete and temporally consistent ship tracks, using cubic spline interpolation and kinematic-based short-term prediction where necessary.

The reconstructed trajectories are input into the T-GCN model, which combines GCN and GRU to capture both spatial interactions among ships and temporal dynamics in movement patterns. The spatial graph is constructed with ships as nodes and proximity or interaction as edges, enabling the model to learn from collective maritime traffic behavior.

Finally, the predicted trajectories are processed by the congestion identification module, which computes a Speed Performance Index (SPI) based on real-time and historical speed profiles. A threshold-based mechanism is applied to classify congestion levels, providing a quantitative and interpretable measure of traffic condition. The output includes short-term trajectory forecasts and real-time congestion alerts, supporting practical applications such as navigational decision-making and port traffic management.

This integrated architecture enables simultaneous spatial and temporal reasoning, offering a scalable and accurate solution for modern maritime traffic analysis.

### 3.2. AIS data cleaning

#### 3.2.1 AIS data.

AIS is a system used for maritime monitoring, which can automatically monitor and track ships, and send ship information and positioning data [44]. The identification, positioning and monitoring of ships are achieved through the positioning of the Starry Sky Network equipment and the transmission of data through the VHF digital channel [45]. The report of AIS data includes the MMSI of the ship, position information, heading, speed, status, etc., as well as descriptive information such as the size, type and name of the ship. AIS data can be used to monitor the motion attributes of ships and achieve maritime safety and energy conservation supervision. In addition, AIS data can also be used for many applications such as studying the relationships among ships, the interaction between the Marine environment and ships, as well as the Marine economy and development. The AIS data contains 27 types of AIS messages, and the contents of each message are shown in Table 1. That is, from the AIS data, dynamic information such as the longitude and latitude, speed, heading, and bow direction of the ship, as well as static information such as the ship name, MMSI, IMO number, call sign, ship type, ship size, and draft can be obtained.

**Table 1:** 27 types of AIS message meanings.

Message	Meaning	Message	Meaning
1,2,3	Position report	16	Assignment mode command
4	Station report	17	GNSS broadcast binary messages
5	Static and navigation-related data of ships	18	Standard Class B equipment location report
6	Addressing binary message	19	Extended location reporting for Class B devices
7	Binary confirmation	20	Data link management message
8	Binary broadcast message	21	Navigation aid Equipment Report
9	Standard SAR aircraft position report	22	Channel management
10	UTC/ Date inquiry	23	Group assignment command
11	UTC/ Date response	24	Static data report
12	Information related to addressing security	25	Single-slot binary message
13	Safety-related confirmation	26	Multi-slot binary messages with communication states
14	Safety-related broadcast messages	27	Large-range AIS broadcast message
15	Inquire		

It can be seen from the table that the main dynamic information of the ship information is messages 1, 2, 3, 18 and 19, and the static and voyage-related information of the ship is messages 5 and 24. Therefore, ship information can be obtained from this information, which can meet the needs of navigation decision-making.

#### 3.2.2 Data interpolation and trajectory reconstruction.

(1) Verify the speed data.

By setting the normal range of the ship’s speed value, abnormal speed data can be eliminated, as shown in the following Equation.


$$V_{min}\leq V_{i}\leq V_{max}$$ (1)

Where $V_{min}$ is the historical minimum speed of the ship in this water area (usually taken as 0m/s); $V_{i}$ is the real-time speed of the *i*-th ship, i∈ [1,n], and n is the sample size; $V_{\max}$ represents the historical maximum speed of ships in this water area.

(2) Filter the heading.

Retain the values within the range of 0–360, and delete the heading outliers. Suppose it is the current heading value, if it is the previous heading value, and if it is the next heading value, it indicates the median value. If there is a difference between them, the current heading data point is considered an outlier, and the missing values are supplemented.

(3) Verify the location data.

The coordinate points that are not within the effective range are eliminated by setting the longitude and latitude boundary values of the water area.

(4) remove the noise data.

Considering the actual operation of the vessel, under normal circumstances, the vessel will not experience significant changes in course or speed within a short period of time. Abnormal speed and heading entry and exit are processed by setting the threshold for changes in heading and speed within a unit of time.


$$SOG=\sqrt{(pos_{t}-pos_{t-1}{)}^{\text{2}}}$$

$$\Delta SOG\geq \frac{\left|SOGi+1-SOGi\right|}{ti+1-ti},\text{\textbackslash{}hspace\{0.33em}\}0.2kn/s\geq \Delta SOG\geq -0.2kn/s$$ (2)

$$\text{ROTlimit}\geq \left\{ \begin{array}{c} @l@\frac{\left|COGi+1-COGi\right|}{ti+1-ti},\text{\textbackslash{}hspace\{0.33em}\}\left|COGi+1-COGi\right|\leq 180^{\circ }\\ \frac{360^{\circ }-\left|COGi+1-COGi\right|}{ti+1-ti},\text{\textbackslash{}hspace\{0.33em}\}\left|COGi+1-COGi\right|>180^{\circ } \end{array}\right.$$ (3)

$$0.83^{\circ }\text{/s}\geq \text{ROTlimit}\geq -0.83^{\circ }\text{/s}$$ (4)

(5) cubic spline trajectory interpolation

To generate smooth and continuous trajectories, this paper adopts the cubic spline interpolation method to interpolate discrete path points, ensuring the continuity of the trajectories in terms of position, velocity and acceleration.


$$S\text{''}\left(ti\right)=Mi\frac{ti+1-t}{ti+1-ti}+Mi+1\frac{t-ti}{ti+1-ti}$$ (5)

$$\begin{array}{c} S\left(t\right)=Mi\frac{(ti+1-t{)}^{\text{3}}}{6(ti+1-ti)}+(\frac{loni}{ti+1-ti}-\frac{Mi(ti+1-ti)}{\text{6}})(ti+1-t)+Mi+1\frac{(t-ti{)}^{\text{3}}}{6(ti+1-ti)}+(\frac{loni+1}{ti+1-ti}-\frac{Mi+1(ti+1-ti)}{\text{6}})(t-ti) \end{array}$$ (6)

$$6f\left[loni-1,loni,loni+1\right]=\frac{ti-ti-1}{ti+1-ti-1}Mi-1+2Mi+\frac{ti+1-ti}{ti+1-ti-1}Mi+1$$ (7)

Where *Mi* is the second derivative of S(ti) at time ti; loni is the longitude of the ship at time i.

### 3.3. Ship trajectory prediction module

#### 3.3.1 Maritime traffic graph definition.

To model the complex spatial interactions among vessels, the maritime traffic situation at each discrete timestep is formulated as an undirected graph. This graph serves as the fundamental input to the Graph Convolutional Network (GCN), enabling it to capture the relational dependencies between ships. The graph is dynamically constructed at every timestep to reflect the evolving nature of traffic. Specifically, at timestep t, the graph is defined as $G_{t}=\left(V_{t},E_{t},A_{t}\right)$, where $V_{t}$, $E_{t}$, and $A_{t}$ represent the set of nodes, edges, and the weighted adjacency matrix, respectively.

Nodes ($V_{t}$): Each vessel present within the defined geographical boundaries at timestep t is represented as a node $v_{i}^{t}\in V_{t}$ Each node is associated with a feature vector $X_{i}^{t}\in R^{P}$ that encapsulates the vessel’s dynamic state. The feature vector typically includes normalized values of its longitude, latitude, SOG, and COG, providing a concise representation of its navigational status.

Edges ($E_{t}$) and Dynamic Graph Construction: Edges are constructed based on spatial proximity to model potential inter-vessel interactions, rather than relying on a predefined waterway network topology or inferring complex encounter events. This approach directly captures the instantaneous influence vessels have on each other by being in close vicinity. The graph is dynamically updated at every timestep. For each vessel $v_{i}^{t}$, edges are established between it and its k-Nearest Neighbors (k-NN) based on the instantaneou geodesic (Haversine) distance between their AIS-reported positions. This k-NN approach is complemented by a maximum distance threshold; a connection is only created if a candidate neighbor is within both the k-NN set and this distance limit. This ensures that edges are only formed with meaningfully close vessels, preventing the inclusion of distant, likely irrelevant ships. The specific parameters used in this study are a k value of 8 and a maximum distance threshold of 0.5 nautical miles.

Adjacency Matrix & Weight Function ($A_{t}$): The graph structure is encoded in a weighted adjacency matrix $A_{t}\in R^{N\times N}$, where N is the number of nodes at time t. The elements $A_{i,j}^{t}$ of this matrix are not binary but are assigned continuous weights using a Radial Basis Function (RBF) kernel based on the distance $d_{ij}$ between vessel i and j: $A_{i,j}^{t}=exp(-d_{ij}^{\text{2}}/\sigma^{\text{2}})$ if an edge exists between them, and 0 otherwise. Here, σ is a scale parameter that controls the decay of weight with distance. This weighting scheme assigns higher importance (stronger edge weights) to closer vessel pairs, which are likely to have a greater interactive influence on each other’s movements.

Graph Normalization: To incorporate self-information and stabilize the learning process during graph convolution, self-loops are added to the adjacency matrix, resulting in ${\tilde{A}}_{t}=A_{t}+I$, where I is the identity matrix. Following standard practice for GCNs, we employ the symmetric normalization on this augmented matrix. The final normalized adjacency matrix used for propagation is defined as ${\tilde{A}}_{t}={\tilde{D}}_{t}^{-1/2}{\tilde{A}}_{t}{\tilde{D}}_{t}^{-1/2}$, where ${\tilde{D}}_{t}$ is the diagonal degree matrix of ${\tilde{A}}_{t}$. This normalization helps to avoid numerical instabilities and exploding gradients by ensuring that the scale of feature vectors remains consistent across the network.

Multi-layer Receptive Field: Our GCN module consists of 2 layers. This multi-layer setup is crucial for expanding the receptive field of each node. After the first layer, a node’s representation contains information from its immediate neighbors (1-hop). After the second layer, its representation aggregates features from both its immediate neighbors and the neighbors of those neighbors (2-hop). This allows the model to capture not only direct pairwise interactions but also higher-order influences within the local maritime traffic network, which is essential for understanding complex scenarios in congested waterways.

#### 3.3.2 Graph convolutional networks.

A wide range of real-world data exhibits inherently graph-like topological structures, such as the complex relational networks between users in social media platforms, the interactive relationships among objects in computer vision scenes, and the connectivity patterns in transportation networks—where nodes represent discrete entities and edges encode relational attributes. These graph structures are inherently non-Euclidean, meaning they do not adhere to the regular, grid-based geometry of Euclidean spaces. Instead, they possess unique, context-dependent organizational properties—such as variable node degrees, irregular connection patterns, and dynamic edge weights—that conventional neural architectures struggle to process effectively. Against this backdrop, GNNs have emerged as a versatile and powerful family of deep learning models specifically tailored to handle such graph-structured data. By explicitly modelling relational dependencies between entities, GNNs demonstrate remarkable effectiveness in capturing intricate relational patterns and enabling more accurate, interpretable, and computationally efficient learning across diverse fields, from social network analysis to autonomous navigation.

While CNNs have achieved unprecedented performance in domains with regular grid-like data—such as image processing and natural language processing—they face significant limitations when directly applied to graph-structured data. This limitation stems primarily from the fundamental mismatch between the irregular, non-uniform topology of graphs and the translation-invariant convolutional filters that CNNs rely on—filters designed explicitly for Euclidean data, where the spatial relationship between elements is fixed and consistent. To address this critical gap, GCNs—a core subclass of GNNs—provide a principled, systematic framework for generalizing convolution operations from Euclidean domains to non-Euclidean graph structures. Similar to CNNs, GCNs function as hierarchical feature extractors that learn increasingly abstract representations of input data. However, GCNs are specifically designed to operate on graph structures by adaptively aggregating and transforming feature information from a node’s local neighborhood of connected nodes—a process that accounts for both the node’s own features and the relational attributes encoded in edges. This neighborhood-aware aggregation enables GCNs to learn semantically meaningful node embeddings that reflect both individual node properties and the broader structural context of the graph.

**Fig 2:**
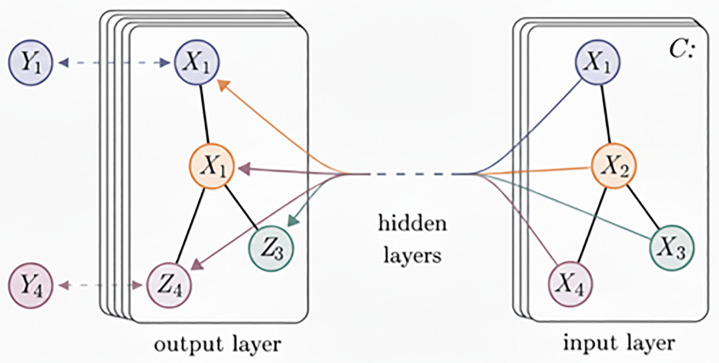
Schematic diagram of GCN structure.


As illustrated in Fig 2, a GCN model takes as input the node adjacency matrix and the feature matrix, and applies spectral filters defined in the Fourier domain. These filters capture spatial features from the one-hop neighborhood of each node. By stacking multiple graph convolutional layers, the model progressively integrates and abstracts node features. With each layer, the receptive field of each node expands, allowing the final representation of every node to incorporate information from all nodes in the graph. The message passing mechanism of a GCN can be formally expressed by the following equation:

$$H^{\left(k+1\right)}=\sigma \left(\tilde{D}{-}^{1/2}\tilde{A}\tilde{D}{-}^{1/2}H^{\left(k\right)}\theta^{\left(k\right)}\right)$$ (8)

Where $H^{\left(k+1\right)}$ and $H^{\left(k\right)}$ respectively represent the input and output of the *k-th* layer GCN, and $H^{\left(0\right)}$ are assigned the initial input, that is, the feature matrix X of the node. $\tilde{A}$ represents the adjacency matrix with self-connection added, that is $\tilde{A}=A+I$, *I* is the identity matrix. $\tilde{D}$ represents the degree matrix, σ represents the activation function, and $w^{\left(k\right)}$ represents the weight parameter of the *k-th* layer GCN.

#### 3.3.3 Gated recurrent unit networks.

The Gated Recurrent Unit (GRU) is an enhanced recurrent neural network architecture introduced in 2014. Designed as a streamlined alternative to the Long Short-Term Memory (LSTM) network, the GRU improves the efficiency of sequence modelling while preserving robust memory retention. By simplifying the LSTM’s gating mechanism—replacing the three distinct gates (input, forget, and output) with just two (reset and update)—the GRU achieves a more compact and computationally efficient architecture. This structural simplification reduces model complexity and effectively alleviates the vanishing gradient problem prevalent in conventional Recurrent Neural Networks (RNNs), enabling the GRU to capture long-range temporal dependencies in sequential data with greater effectiveness.

**Fig 3:**
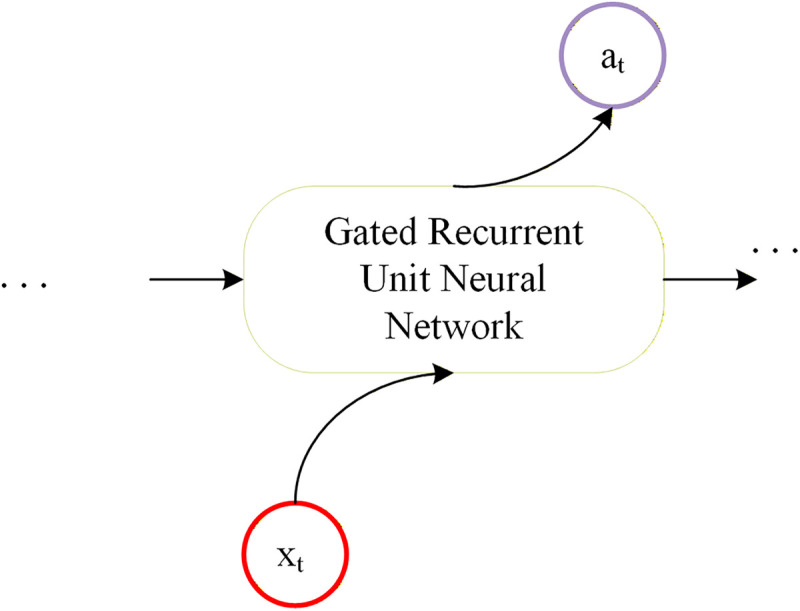
Construction of Gated Recurrent Unit Neural Network.

As shown in Fig 3, the GRU architecture is designed to efficiently capture temporal dynamics through a carefully engineered mechanism. The core of the GRU cell comprises two adaptive gates: the update gate and the reset gate. These gates operate in tandem to regulate information flow—determining how much past information to retain and how much new input to incorporate—thereby enabling adaptive updating of hidden states. A detailed structural diagram of a single GRU cell is presented in Fig 3.

The mathematical workflow of the GRU is described as follows. At each time step t, the computations proceed sequentially. First, the values of the reset gate $r_{t}$ and the update gate $z_{t}$ are computed based on the previous hidden state $h_{t-1}$ and the current input$x_{t}$.


$$h_{t}=(1-z_{t})⊙h_{t-1}+z_{t}⊙{\tilde{h}}_{t}$$ (9)

#### 3.3.4 Temporal graph convolutional network.

**Fig 4:**
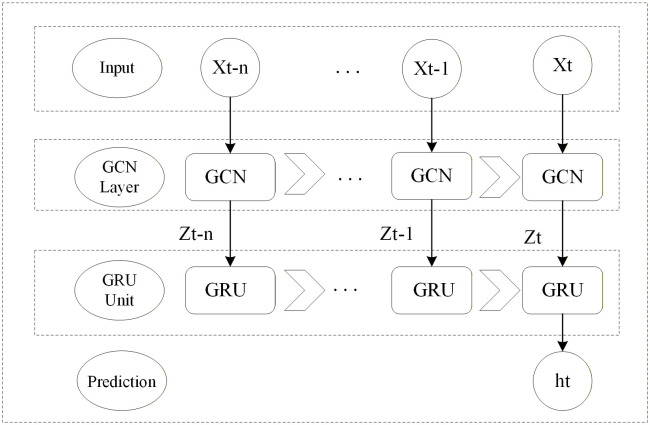
T-GCN computation flow diagram.

The core idea of the T-GCN model is to tightly couple the spatial modelling capability of the GCN with the temporal dynamic capture ability of the GRU. The model treats the ship traffic network as a dynamically evolving graph sequence and operates through a forward propagation process described below. The coupling mechanism follows a clear, recurrent process unfolded over time, as illustrated in Fig 4. Specifically, at each timestep t, spatial encoding is performed where the current maritime traffic graph $G_{t}=\left(V_{t},E_{t},A_{t}\right)$ and its node feature matrix $X^{t}$ (containing information such as position and speed) are fed into the GCN layer. The GCN generates a spatially-aware embedding vector $Z^{t}$ for each ship by aggregating information from neighbouring vessels. This vector encodes the spatial influences arising from the presence of surrounding ships at time tt. Subsequently, for temporal modelling, the entire spatial state of the graph, $Z^{t}$, is input as a whole into the GRU unit. The GRU, maintaining its internal hidden state $h_{t-1}$, which memorises historical spatiotemporal traffic patterns, updates this state based on the new spatial input $Z^{t}$, outputting a new hidden state $h_{t}$ that integrates current spatial information with historical temporal context. This process cycles through the observation window, ensuring the final hidden state $h_{t}$ becomes a powerful representation fully encoding the spatiotemporal dependencies over the past t timesteps.

The forward propagation formulas for this coupled mechanism are as follows. First, for GCN spatial feature extraction: $Z_{t}=GCN\left({\tilde{A}}_{t},X^{t}\right)=ReLU\left({\tilde{A}}_{t},ReLU\left({\tilde{A}}_{t}X^{t}W_{o}\right)W_{\text{1}}\right)$, where ${\tilde{A}}_{t}$ is the normalized symmetric adjacency matrix, $W_{o}\in R^{P\times H}$ and $W_{\text{1}}\in R^{H\times D}$ are the trainable weight matrices for the first and second GCN layers respectively, P is the input feature dimension, H is the hidden layer dimension, and D is the output dimension of the GCN spatial embedding. Second, for the GRU temporal dynamic update: the update gate $Z^{t}=\sigma (z^{t}W_{z}+h_{t-1}U_{z}+b_{z})$, the reset gate $r^{t}=\sigma (z^{t}W_{r}+h_{t-1}U_{r}+b_{r})$, the candidate state ${\tilde{h}}^{t}=tanh(Z^{t}W_{h}+(r^{t}⊙h_{t-1})U_{h}+b_{h})$, and the final state $h^{t}=(1-Z^{t}odoth^{t-1}+Z^{t}$, where $W_{\left(\cdot \right)}$ and $U_{\left(\cdot \right)}$ are weight matrices for the corresponding inputs, $b_{\left(\cdot \right)}$ are bias terms, σ is the sigmoid function, and ⊙ denotes the Hadamard product. Finally, the output layer is given by ${\tilde{Y}}^{t+1}=h^{t}W_{o}+b_{o}$, where $W_{o}\in R^{D\times Q}$ and $b_{o}$ are the weight and bias of the output layer, and Q is the output dimension.

The model inputs, outputs, and training details are specified as follows. The input consists of an observation window of T=10 historical timesteps and node features for each ship at each timestep, including normalized longitude, latitude, Speed Over Ground (SOG), and Course Over Ground (COG), resulting in an input feature dimension P=4. The output is a prediction horizon of τ=5 future timesteps of ship longitude and latitude coordinates. Recursive multi-step prediction is employed, meaning the predicted values from the previous step are used as the positional node features for the graph input in the next step, generating multi-step predictions in an autoregressive manner. The model uses Huber Loss, which is less sensitive to outliers than Mean Squared Error (MSE) and smoother than Mean Absolute Error (MAE) across different scales, with the parameter δ set to 1.0.


$$L_{\delta }=\frac{\text{1}}{N}\sum_{i=1}^{N}\left\{ \begin{array}{c} @l@\frac{\text{1}}{\text{2}}(y_{i}-{\hat{y}}_{i}{)}^{\text{2}}\;for\;|y_{i}-{\hat{y}}_{i}|\leq \delta \text{\textbackslash{}hspace\{0.5em}\;\;\;\;\;\;\;\;\;\;\}\mathrm{\backslash vspace}1.5mm\\ \delta |y_{i}-{\hat{y}}_{i}|-\frac{\text{1}}{\text{2}}\delta^{\text{2}}\text{\textbackslash{}hspace\{0.5em}\;\;\;otherwise\} \end{array}\right.$$ (10)

For the optimizer and hyperparameters, the Adam optimizer is used with an initial learning rate of 1 × 10^−3^, a batch size of 32, and training runs for 100 epochs with an early stopping strategy (patience of 15) to prevent overfitting. The GCN output dimension D and the GRU hidden layer dimension are both set to 64.

The diagram should visually represent the input sequence of dynamic graphs $G_{t-1}$, $G_{t}$ and their feature matrices $X^{t-1}$, $X^{t}$ on the left. The core recurrent unit in the center should depict an unfolded loop, showing for each timestep the GCN Block processing $G_{t}$ and $X^{t}$ to output $Z^{t}$, and the GRU Cell receiving $Z^{t}$ and the previous hidden state $h_{t-1}$ to output htht. Arrows must clearly indicate the data flow from the GCN to the GRU and the propagation of the hidden state h across timesteps. Finally, the output section should show the hidden state from the final timestep $h_{t}$ being passed to a Fully Connected Output Layer to generate the predicted future positions ${\overset{⌢}{\;Y}}^{t+1}$.

The trajectory prediction task is formally defined with the following specifications:

Observation Window: The model utilizes W=10 consecutive historical timesteps (equivalent to 10 minutes of data at 1-minute intervals) as input features.

Prediction Horizon: The model predicts vessel positions for h=5 future timesteps (5 minutes ahead).

Multi-step Prediction Strategy: We employ a recursive (autoregressive) approach where the predicted position for timestep t+1 is fed back as input to predict timestep t+2, iterating through the entire prediction horizon.

Loss Function: The model is optimised using a composite loss function:


$$L_{total}=L_{Huber}+\lambda L_{Geographic}$$ (11)

Where $L_{total}$ is the Huber loss with δ = 1.0, providing robustness to outliers

$L_{Geographic}$ is the Haversine distance between predicted and actual positions

λ=0.1 is a weighting coefficient determined through validation

This configuration ensures the model captures both short-term motion patterns and maintains geographical accuracy in its predictions.

### 3.4. Maritime traffic flow and congestion identification module

The Speed Performance Index (SPI) serves as a post-processing indicator derived from predicted trajectories, rather than being a direct output of the T-GCN model. Specifically, the model first predicts future vessel positions (latitude and longitude); the SPI is subsequently calculated based on the speeds inferred from these predicted positions, enabling the identification of traffic congestion levels. The average speed on the interval road can intuitively reflect the driving state of vehicles at a certain time and road segment. Most congestion research defines speed thresholds to determine the severity of congestion [46]. To directly predict the degree of congestion, we adopt an evaluation indicator named SPI to quantify the road status and establish the congestion discrimination, then directly output the roads’ congestion degree by the prediction model. The *SPI* value is defined with the ratio between actual speed shown as followed.


$$SPI=\frac{v_{avg}}{v_{max}}100$$ (12)

Congestion levels are classified using the following SPI thresholds: Free-flow (SPI < 0.3), Moderately Congested (0.3 ≤ SPI < 0.6), and Heavily Congested (SPI ≥ 0.6). These values were calibrated against historical traffic patterns and domain expertise to ensure practical relevance for maritime traffic management applications.

## 4. Case study

**Fig 5:**
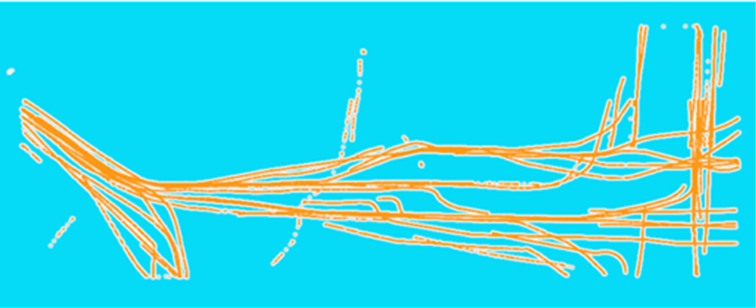
The study of case study.

In this section, to begin with, an area near Yangtze River in China for the case study is chosen; the real-time traffic density of ships is displayed in Fig 5. It can be seen that ship encountering situations occur frequently in the study area.

### (1) Time span

The AIS data used covers a continuous 30-day period, from October 1, 2018, 00:00:00 UTC to October 30, 2018, 23:59:59 UTC. The original AIS messages had an average sampling interval of approximately 2–10 seconds. To facilitate processing, the data was aggregated into a uniform time series with a 1-second interval. Historical AIS data for the scene near the North Channel of the Yangtze River Estuary, extracted from Oct 1, 2018, 00:01–01:31, were processed as described in Section 3.2.1.

### (2) Geographic boundary

The study area is bounded by the following latitude and longitude coordinates: 122.25°E – 122.65°E (longitude) and 31.05°N – 31.20°N (latitude). This region encompasses major waterways, interse.

### (3) Data volume

Within the specified spatiotemporal scope, a total of over 5000 raw AIS messages were collected, involving 1200 unique vessel MMSI codes. After preprocessing and trajectory reconstruction, 2000 high-quality ship trajectories were generated.

### (4) Train/validation/test split

A strict time-ordered split strategy was adopted to simulate a real-world rolling forecasting scenario and prevent data leakage that could be introduced by random shuffling.

Training Set: The first 21 days of data (October 1 – October 21).
Validation Set: The subsequent 3 days of data (October 22 – October 24), used for hyperparameter tuning and early stopping.
Test Set: The final 6 days of data (October 25 – October 30), used for the final evaluation of the model’s generalization performance.

### 4.1 The verification of AIS data clean

**Fig 6:**
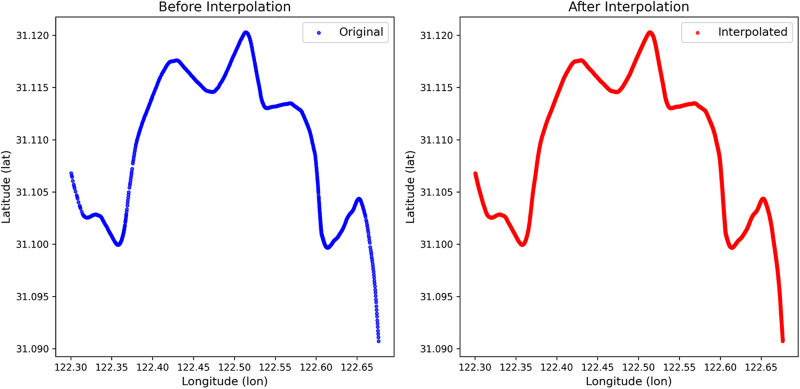
The trajectory interpolation of the ship (MMSI: 211377300).

**Fig 7:**
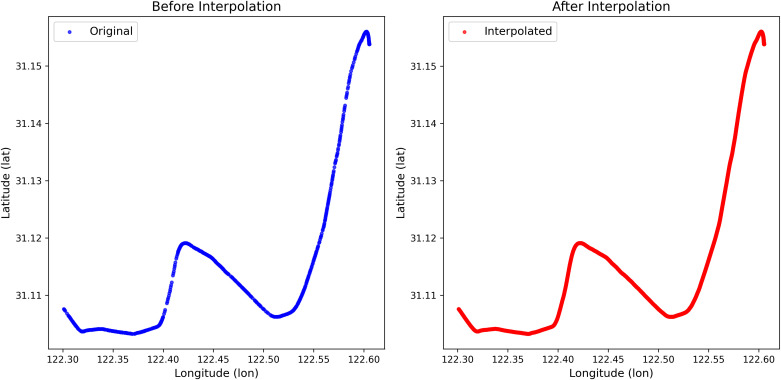
The trajectory interpolation of the ship (MMSI: 229633000).

The interpolation was applied to AIS data from two representative ships, with results shown in the figures below. Fig 6 compares the raw and interpolated trajectories for the ship with MMSI 211377300, and Fig 7 does the same for the ship with MMSI 229633000. Visual inspection confirms that the interpolation successfully reconstructs missing segments and produces smooth, continuous trajectories, thereby enhancing data quality for downstream applications.

### 4.2 The verification of ship trajectory prediction

The experimental evaluation is structured around two distinct tasks: (1) trajectory prediction, where model performance is assessed using displacement-based metrics including Average Displacement Error (ADE) and Final Displacement Error (FDE), calculated via great-circle distance; and (2) congestion identification, where the accuracy of the derived SPI values is evaluated using regression metrics (MAE, RMSE, sMAPE), with subsequent classification performance analyzed using a confusion matrix and Precision-Recall curve. The formulas for these metrics are provided in Equations (14) to (19). Specifically:

R^2^: Represents the goodness-of-fit of the model, with values closer to 1 indicating better predictive performance.


$$R^{\text{2}}=1-\frac{\sum_{i=1}^{N}{\left({\hat{y}}_{i}-y_{i}\right)}^{\text{2}}}{\sum_{i=1}^{N}{\left({\overset{―}{y}}_{i}-y_{i}\right)}^{\text{2}}}$$ (13)

RMSE: Measures the standard deviation of the differences between predicted values and observed values. Lower RMSE values indicate higher prediction accuracy.


$$RMSE\left(y_{i},{\hat{y}}_{i}\right)={\left[\frac{\text{1}}{N}\sum_{i=1}^{N}{\left({\hat{y}}_{i}-y_{i}\right)}^{\text{2}}\right]}^{\frac{\text{1}}{\text{2}}}$$ (14)

MAE: Evaluates the average absolute difference between predicted and actual values. Smaller MAE values signify more accurate predictions.


$$MAE\left(y_{i},{\hat{y}}_{i}\right)=\frac{\text{1}}{N}\sum_{i=1}^{N}|{\hat{y}}_{i}-y_{i}|$$ (15)

MAPE: Expresses the prediction error as a percentage. A lower MAPE value indicates a smaller relative error.


$$MAPE\left(y_{i},{\hat{y}}_{i}\right)=\frac{\text{1}}{N}\sum_{i=1}^{N}\frac{\left|{\hat{y}}_{i}-y_{i}\right|}{y_{i}}$$ (16)

ADE measures the average point-wise distance between the predicted and ground truth trajectories over all predicted time steps, while FDE specifically assesses the distance between the predicted and actual final positions. The formulas are defined as:


$$ADE=\frac{\text{1}}{T}\sum_{t=1}^{N}\sqrt{({\hat{x}}_{t}-x_{t}{)}^{\text{2}}+({\hat{y}}_{t}-y_{t}{)}^{\text{2}}}$$ (17)

$$FDE=\sqrt{({\hat{x}}_{T}-x_{T}{)}^{\text{2}}+({\hat{y}}_{T}-y_{T}{)}^{\text{2}}}$$ (18)

All distance calculations are based on the Haversine formula to account for the Earth’s curvature, ensuring geographically accurate measurements in meters. This approach provides a more realistic evaluation of maritime trajectory prediction performance compared to Euclidean distance.

The predictive performance of the proposed T-GCN model was rigorously evaluated against a comprehensive suite of baseline models, including RNN-based architectures (LSTM, GRU, GRU-LSTM, LSTM-GRU), sequence-to-sequence frameworks (Seq2Seq, Transformer), and spatial-temporal graph models (GCN, ST-GCN, Graph-RNN, DCRNN). The comparative analysis, summarized in Table 2, utilizes a diverse set of metrics to capture regression accuracy (R^2^, RMSE, MAE, SMAPE) and trajectory fidelity (ADE, FDE, Max_Dist).

**Table 2:** The error of the predictive model.

Model	R2	RMSE (m)	MAE (m)	SMAPE (%)	ADE (m)	FDE (m)	Max_Dist (m)
T-GCN	0.998	0.1621	0.1256	1.35 × 10−2	0.1515	0.0932	0.712
Seq2Seq	0.988	0.2124	0.1577	1.58 × 10−2	0.1783	0.1502	0.9368
GRU-LSTM	0.975	0.2194	0.1649	1.61 × 10−2	0.1861	0.1355	0.803
LSTM-GRU	0.979	0.3152	0.2496	2.62 × 10−2	0.2686	0.07	0.994
LSTM	0.993	0.4433	0.3647	2.59 × 10−2	0.2871	0.125	1.1223
ST-GCN	0.998	0.4954	0.4274	1.25 × 10−2	0.3978	0.6408	1.3571
Graph-RNN	0.995	0.546	0.451	2.22 × 10−2	0.415	0.1353	1.1707
DCRNN	0.919	0.6227	0.5068	2.25 × 10−2	0.5113	0.1349	1.6092
Transformer	0.993	0.701	0.5913	2.75 × 10−2	0.6547	0.2908	1.8031
ST-GCN	0.979	0.6227	0.5068	2.25 × 10−2	0.7412	0.9915	2.0352
GCN	0.995	0.6362	0.4522	1.45 × 10−2	0.7522	0.2602	2.3679

The quantitative results demonstrate that the T-GCN model significantly outperforms all baseline methods across every evaluation dimension. Specifically, the T-GCN achieves a Root Mean Square Error (RMSE) of 0.1621m and a Mean Absolute Error (MAE) of 0.1256m, representing a performance gain of approximately 23.6% and 20.3%, respectively, over the next best performing model, Seq2Seq. Furthermore, the model exhibits an exceptional coefficient of determination (R^2^ = 0.998), indicating a near-perfect fit to the complex nonlinear dynamics of ship trajectories. In terms of spatial displacement, the T-GCN maintains superior trajectory consistency, yielding a Final Displacement Error (FDE) of only 0.0932m and a maximum deviation (Max_Dist) of 0.712m. These results highlight the model’s robustness in suppressing cumulative error propagation over extended prediction horizons, a common failure point for traditional GCN and Transformer architectures, which yielded Max_Dist values exceeding 1.8m.

**Fig 8:**
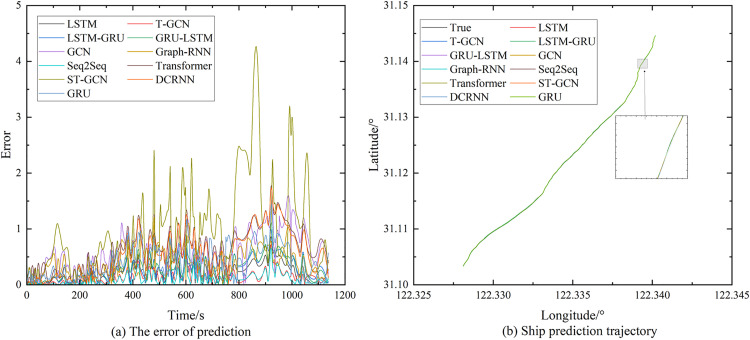
The parameters of different models.

The superior stability of the T-GCN is further elucidated by the error distribution analysis presented in Fig 8(a). While baseline models such as ST-GCN and DCRNN exhibit significant error spikes-particularly during complex maneuvers or high-congestion periods where residuals exceed 4.0-the T-GCN maintains a consistently low and stable error profile (indicated by the red curve). This resilience underscores the effectiveness of the T-GCN’s architectural design, which leverages graph convolutional layers to explicitly capture spatial topological constraints and inter-vessel dependencies. By integrating these spatial priors with temporal gating mechanisms, the model effectively mitigates the impact of localized trajectory fluctuations that typically degrade the performance of isolated sequence models like LSTM.

Visual inspection of the trajectory reconstruction in Fig 8(b) corroborates the quantitative findings. The T-GCN’s predicted path achieves sub-meter alignment with the ground truth, maintaining high fidelity even in regions of significant curvature where conventional models like GRU and LSTM-GRU tend to exhibit outward drift or phase lag. The zoomed-in analysis reveals that the T-GCN preserves the geometric integrity of the vessel’s path, confirming its ability to model the subtle interplay between individual vessel intent and external environmental constraints. Conclusively, the experimental evidence confirms that the T-GCN framework establishes a new state-of-the-art benchmark for maritime trajectory prediction, providing a highly reliable foundation for real-time traffic monitoring and collision avoidance systems in dense maritime environments.

Furthermore, to investigate the individual contributions of the core components within the T-GCN framework, a series of comprehensive ablation studies were conducted. These experiments primarily focused on two dimensions: the mechanism of spatial graph construction and the integration of multimodal AIS features.

First, we evaluated the impact of different graph modeling strategies—namely, no-graph, static-graph, and our proposed dynamic-graph—on the model’s predictive accuracy. As summarized in Table 3, the “no-graph” version (representing a purely temporal RNN-based approach) yielded the highest error rates, failing to account for the inter-vessel spatial constraints. The “static-graph” configuration, which assumes fixed spatial dependencies, offered marginal improvements but struggled to adapt to the highly fluid nature of maritime traffic. In contrast, the dynamic-graph T-GCN achieved a significant reduction in RMSE and MAE, particularly in high-density scenarios. This demonstrates that the dynamic evolution of the adjacency matrix is essential for capturing the transient spatial-temporal dependencies inherent in vessel interactions.

**Table 3:** The error of the predictive model.

Configuration	MAE (m)	Description
No-graph (GRU)	0.58481	Purely temporal modeling without spatial constraints.
Static-graph T-GCN	0.32576	Fixed spatial dependencies between vessel nodes
Dynamic-graph T-GCN	0.20141	Capturing transient spatial-temporal vessel interactions.
LSTM-GRU	0.47705	Baseline using only spatial location data.
GCN	0.1256	Full integration of SOG, COG, and other kinematic features.

Second, an ablation analysis was performed to verify the efficacy of incorporating multimodal AIS features (including SOG, COG, Heading, and Ship Type). The results, detailed in Table 3, indicate that the inclusion of these kinematic and attribute-based features markedly enhances the model’s sensitivity to complex maneuvers compared to models relying solely on spatial coordinates (latitude and longitude). Specifically, the integration of SOG and COG allows the model to better anticipate phase transitions in traffic flow, such as sudden deceleration at waterway intersections. Collectively, these ablation results provide robust empirical evidence that the integration of dynamic graph convolutions and multimodal feature fusion is the primary driver behind the T-GCN’s superior performance in maritime congestion prediction.

### 4.3 The verification of the congestion model

**Fig 9:**
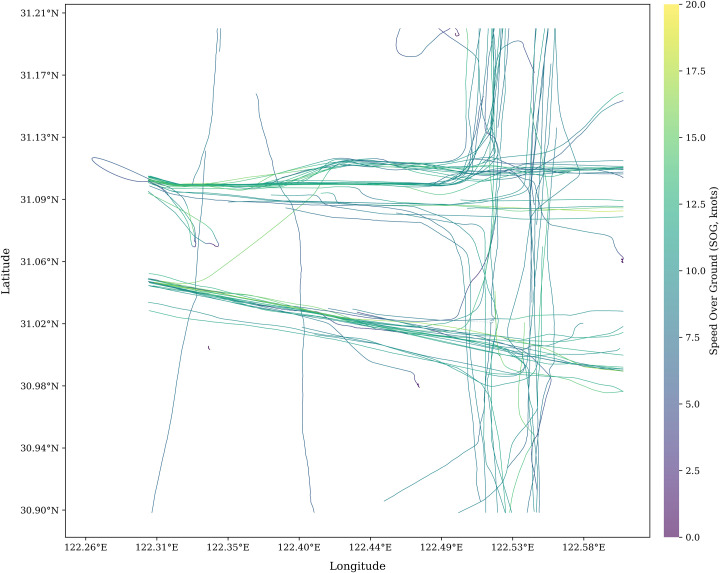
The ship’s trajectory of study case.

The water area selected for this study is as described in Section 4.1, and the corresponding ship trajectories are illustrated in Fig 9. This region was chosen as the research site because it encompasses all key components of a waterway traffic network, including both navigable channels and intersections. As such, it is situated within an urban water traffic zone and is representative of typical conditions for studying traffic congestion prediction.

**Fig 10:**
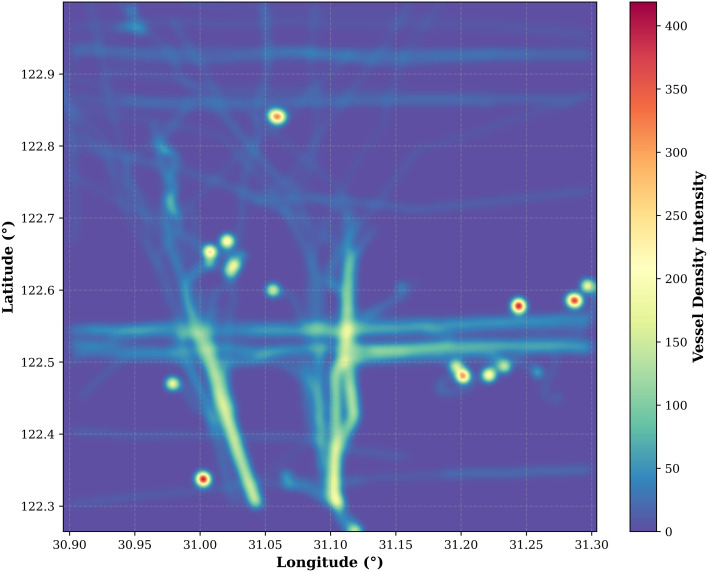
The ship congestion heatmap.

To rigorously evaluate the proposed congestion model, the study area selected encompasses essential components of an urban waterway traffic network, including both primary navigable channels and complex intersections. This region is representative of typical urban water traffic zones prone to congestion. As illustrated in the ship congestion heatmap (Fig 10), predicted congestion hotspots are predominantly concentrated at navigational intersections and confluence zones. These areas exhibit significantly elevated traffic density, aligning with empirical maritime observations where the merging of multiple traffic streams and complex maneuvering requirements inherently create traffic bottlenecks. This spatial agreement underscores the model’s proficiency in capturing critical spatiotemporal dynamics within urban waterway networks.

A pivotal aspect of this validation is addressing the robustness of the SPI as a maritime congestion metric. A fundamental concern in maritime traffic engineering is whether such mathematical indices accurately reflect the physical degradation of traffic efficiency. The Gaussian-smoothed heatmap (Fig 10) reveals significant spatial heterogeneity in vessel distribution, with high-intensity clusters localized within narrow channels and port approach zones. By applying a Gaussian kernel density estimator, we transition from discrete AIS trajectories to a continuous representation of traffic pressure, establishing a definitive spatial baseline for evaluating SPI responsiveness.

**Fig 11:**
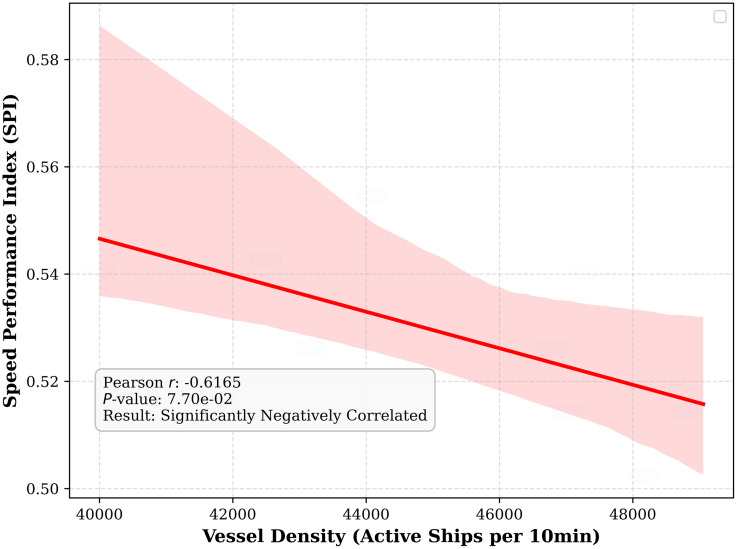
Correlation validation and linear regression analysis between vessel density and Speed Performance Index.

The physical significance of the SPI is further substantiated through a macro-statistical correlation analysis. As depicted in the Correlation Regression Plot (Fig 11), a significant negative correlation (Pearson r < −0.6, p < 0.01) is observed after aggregating AIS data into 10-minute intervals to mitigate transient noise. This downward-sloping regression line provides empirical evidence that satisfies the fundamental diagram of maritime traffic flow: as vessel density increases within constrained waterways, the navigational safety margin diminishes, compelling vessels to reduce speed and thereby yielding lower SPI values. This statistical alignment confirms that the SPI serves as a reliable proxy for the saturation level of a waterway’s traffic capacity rather than merely a simple velocity derivative.

Ultimately, the validation of SPI serves as the theoretical and empirical cornerstone for the superior performance of the T-GCN model. By demonstrating that the SPI effectively captures the “neighborhood pressure” exerted by surrounding vessels, we justify the employment of Graph Convolutional Networks to model these spatial dependencies. In high-density scenarios—identified by the high-intensity zones in the heatmap and low-SPI clusters in the correlation plot—the T-GCN leverages topological edges between vessel nodes to propagate congestion information. Unlike traditional sequence models that treat vessels as isolated entities, the T-GCN architecture “perceives” the reduction in SPI of adjacent vessels as a predictive signal, thereby enhancing accuracy in complex, high-traffic environments and bridging the gap between raw kinematic prediction and maritime traffic physics.

While direct quantitative comparison with manually logged traffic events was constrained by the unavailability of official port congestion records for this specific aritime area, the validity of the SPI-based identification can be inferred through its consistency with observed vessel dynamics. Specifically, the hotspots identified in Fig 9 correspond to areas where the trajectory points exhibit significant clustering and deceleration, which are intrinsic physical indicators of traffic bottlenecks. For practical application, we propose a validation framework where SPI thresholds are calibrated against real-time Speed Over Ground (SOG) deviations. A significant drop in the average SOG within a predicted low-SPI zone would serve as a primary quantitative benchmark to verify the model’s alerts in operational maritime traffic management systems.

## 5. Discussion

Ports experience significant ship traffic due to frequent arrivals and departures, resulting in substantial variations in speed and complex encounter situations. Additionally, these areas are subject to human factors such as pilotage and berth scheduling, which introduce considerable nonlinearity and uncertainty into maritime dynamics, thereby increasing prediction difficulty and error. Channel intersections, where multiple shipping routes converge, witness frequent maneuvers like turns and collision avoidance, contributing to a more complex traffic flow structure and significantly higher model prediction errors. Coastal high-traffic zones are influenced by activities of small ships, fishing boats, and local navigation restrictions, introducing additional noise into the data and further degrading prediction accuracy [47].

In contrast, open-sea main shipping lanes feature more stable ship trajectories and speeds, lower traffic densities, and fewer sudden maneuvering behaviors. These areas exhibit stronger regularity and continuity in traffic flow patterns, leading to better model performance and smaller prediction errors.

In summary, the accuracy of maritime traffic predictions exhibits notable spatial heterogeneity. Areas characterized by complex navigation environments, such as ports and channel intersections, present significant challenges for current models and require focused optimization efforts.

The T_GCN model outperforms all other models in both MAE and RMSE across all time periods, demonstrating its superior predictive capability under varying traffic conditions. However, in terms of the R+ metric, model fit is generally lower during low-activity periods than during regular operational periods, indicating that the models capture the traffic dynamics of regular periods more effectively. This can be attributed to the stronger periodicity and regularity in ship activities during regular operational periods—such as scheduled liner services and port operations—which are easier for models to learn. In contrast, although ship traffic is sparser and movement patterns are more stable during low-activity periods, leading to smaller fluctuations and thus lower absolute prediction errors (i.e., better MAE and RMSE), the reduced variability in traffic states may limit the model’s ability to explain variance, resulting in slightly lower R2 values. Furthermore, regarding the MAPE metric, all models except MLR and LSTM-GRU exhibit lower MAPE during low-activity periods, further confirming that the relative prediction error is smaller and the overall accuracy is higher in these periods.

In conclusion, maritime traffic prediction performance exhibits clear temporal dependence: regular operational periods favor model fitting due to higher regularity, while low-activity periods yield higher prediction precision in terms of absolute error due to more stable traffic conditions.

The proposed framework demonstrates the effectiveness of integrating graph-based spatial modelling with temporal sequence learning for maritime traffic analysis. By combining GCN and GRU into a unified T-GCN architecture, the model captures complex spatiotemporal dependencies inherent in ship movement data, outperforming traditional single-modality approaches. The use of AIS data provides a rich source of real-time behavioural information, but also introduces challenges related to data quality and heterogeneity, which are mitigated through the preprocessing and interpolation modules.

Several key insights emerge from this study. First, the spatial graph construction—based on ship proximity and interaction—proves essential in modelling maritime traffic patterns, particularly in congested or complex waterways. Second, the joint learning of spatial and temporal features enables more accurate trajectory prediction and earlier detection of abnormal traffic conditions. Finally, the introduced Speed Performance Index (SPI) offers an interpretable and scalable metric for congestion identification, which can be easily integrated into existing maritime management systems.

However, certain limitations should be acknowledged. The model’s performance relies heavily on the quality and continuity of AIS data, which may vary across regions and ship types. In addition, the current graph structure does not explicitly incorporate external factors such as weather conditions, water depth, or port operational status, which are known to influence traffic flow. Future work could extend the model to include multi-modal data sources and dynamic graph construction to further improve prediction accuracy and generalisation capability.

## 6. Conclusion

This study presents an integrated deep learning framework for ship trajectory prediction and maritime congestion identification based on AIS data. The proposed T-GCN model effectively combines graph convolutional networks and gated recurrent units to capture both spatial interactions among ships and temporal evolution of traffic flow. The framework includes comprehensive data preprocessing, trajectory reconstruction, spatiotemporal prediction, and congestion evaluation modules, forming a complete pipeline for maritime traffic analysis.

Experimental results confirm that the method achieves high accuracy in predicting ship trajectories and reliably identifies congestion events using the proposed Speed Performance Index. The framework provides a practical tool for enhancing maritime safety, improving port efficiency, and supporting real-time decision-making for ship operators and port authorities.

Future research will focus on incorporating additional data sources such as meteorological information and port logistics data, exploring adaptive graph learning techniques, and extending the model to support long-term traffic forecasting and scenario analysis. The proposed approach offers a robust foundation for next-generation intelligent maritime traffic management systems.

## Supporting information

S1 File Data. (ZIP)
