# Supplementary figures and images for: Maritime traffic congestion identification and ship trajectory prediction using temporal graph convolutional networks
Source: PLoS One. 2026 Mar 9;21(3):e0342781. doi: 10.1371/journal.pone.0342781 (PMC12970927; doi:10.1371/journal.pone.0342781)

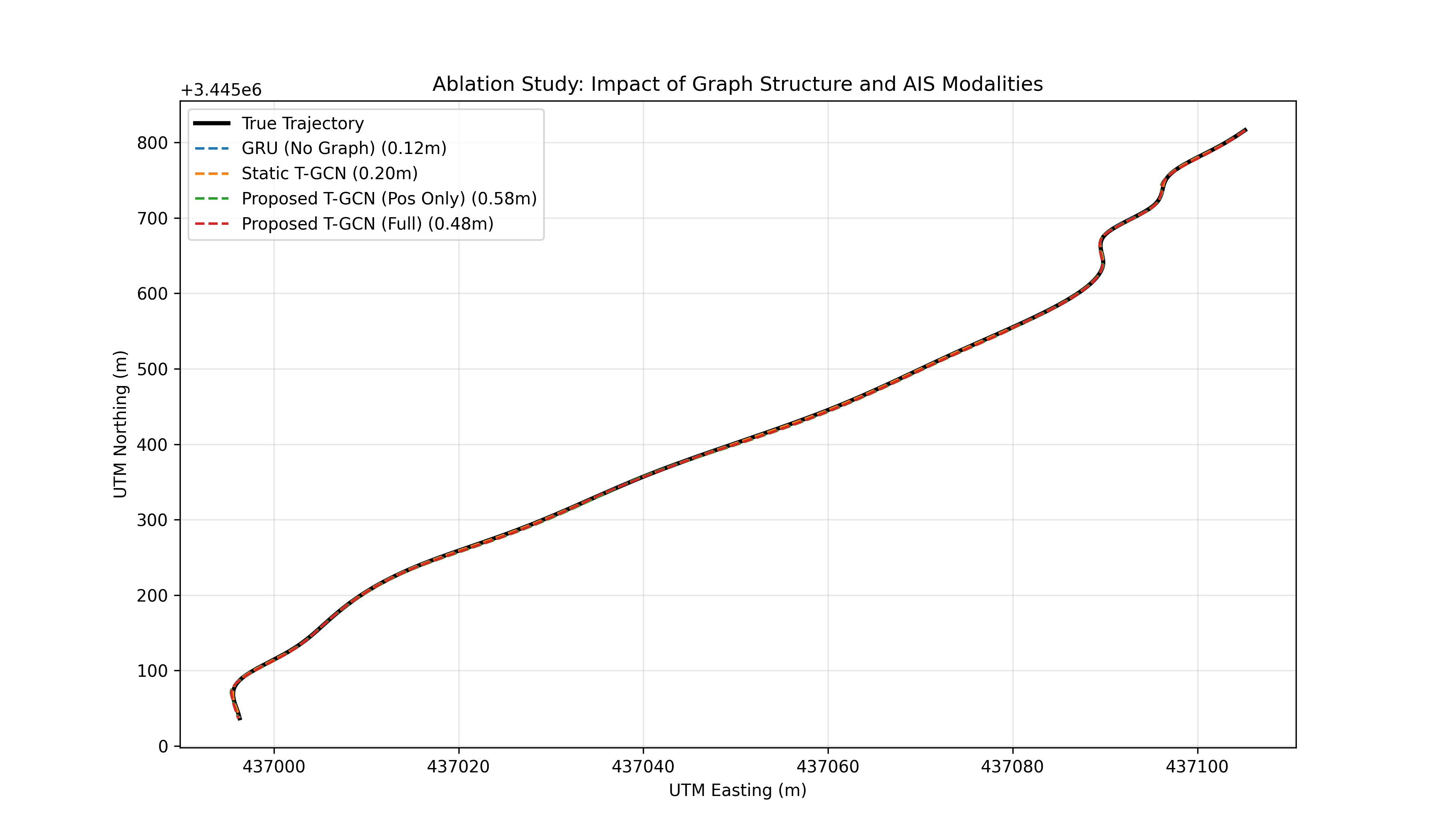

Supplement: S1 File — (ZIP) [file pone.0342781.s001.zip › data/interpolation/Ablation_Study_Comparison.png]

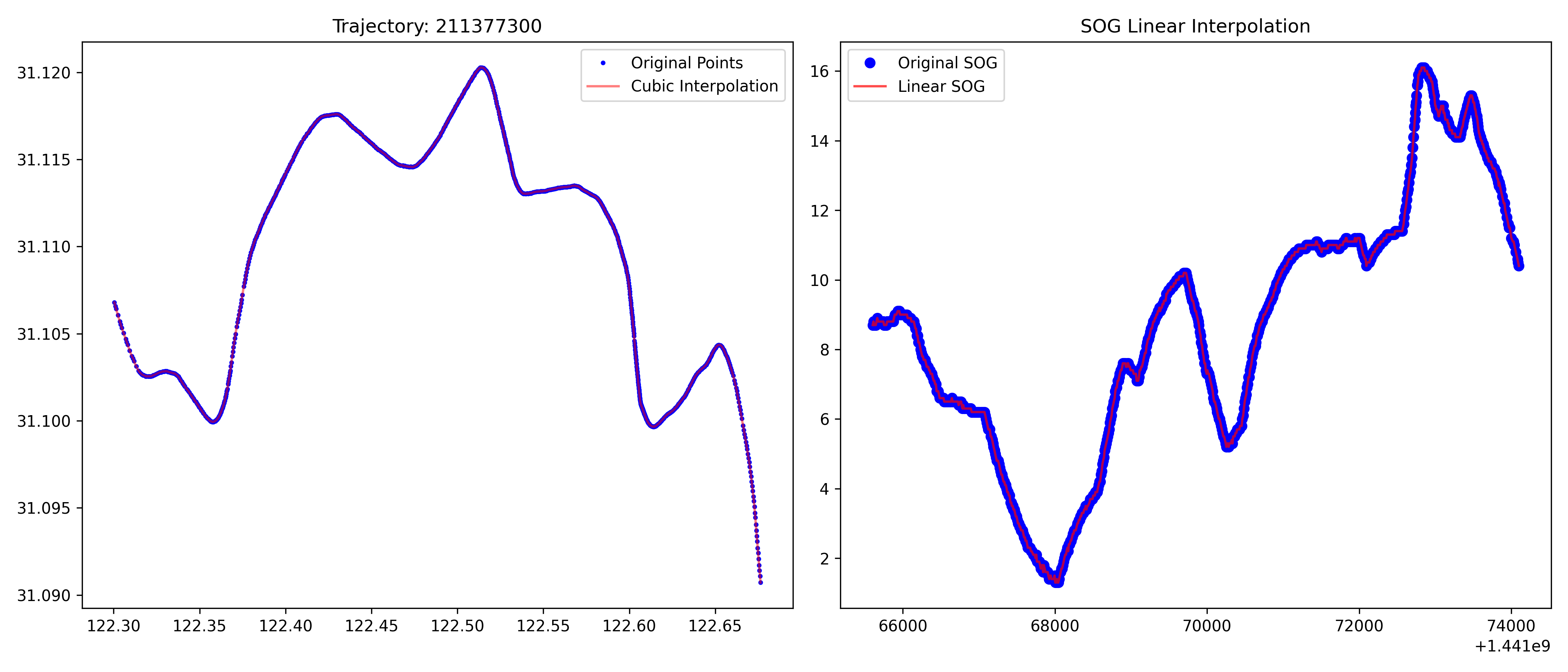

Supplement: S1 File — (ZIP) [file pone.0342781.s001.zip › data/interpolation/shipid_211377300_plot.png]

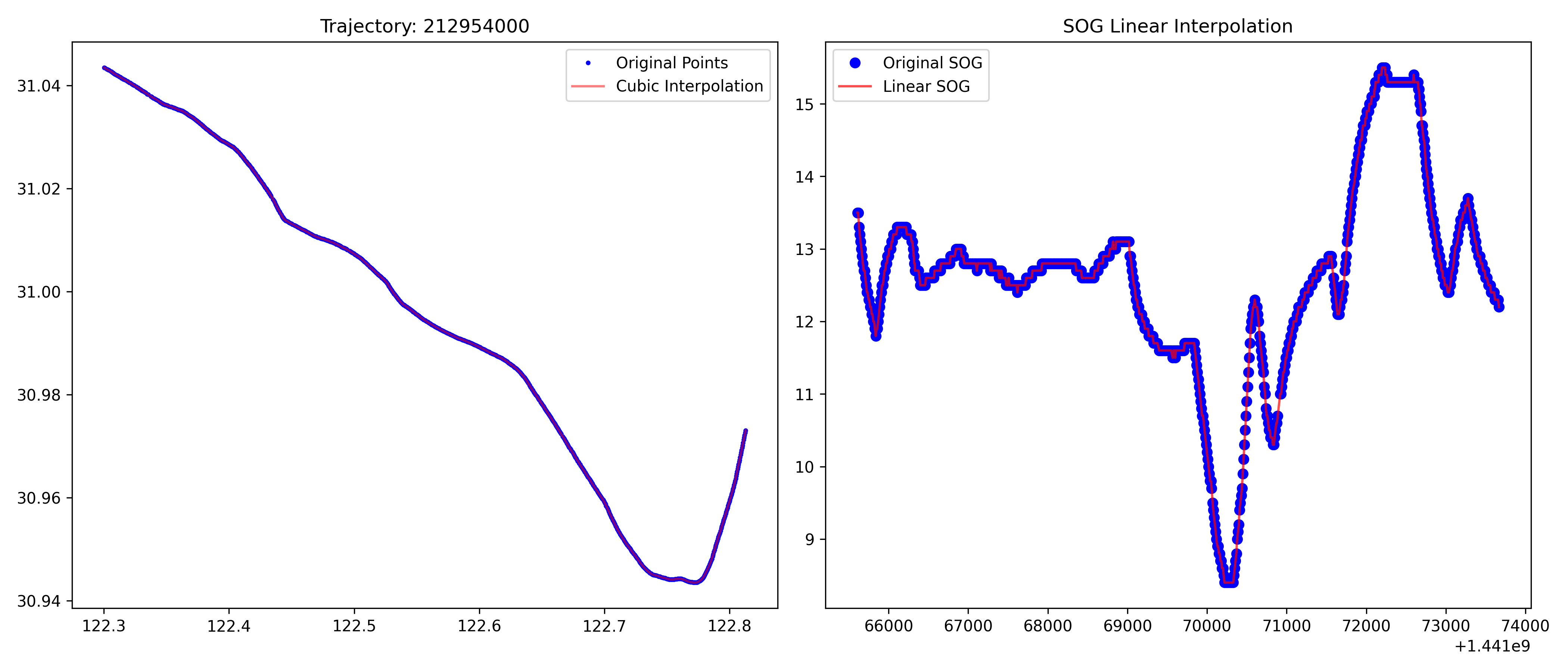

Supplement: S1 File — (ZIP) [file pone.0342781.s001.zip › data/interpolation/shipid_212954000_plot.png]

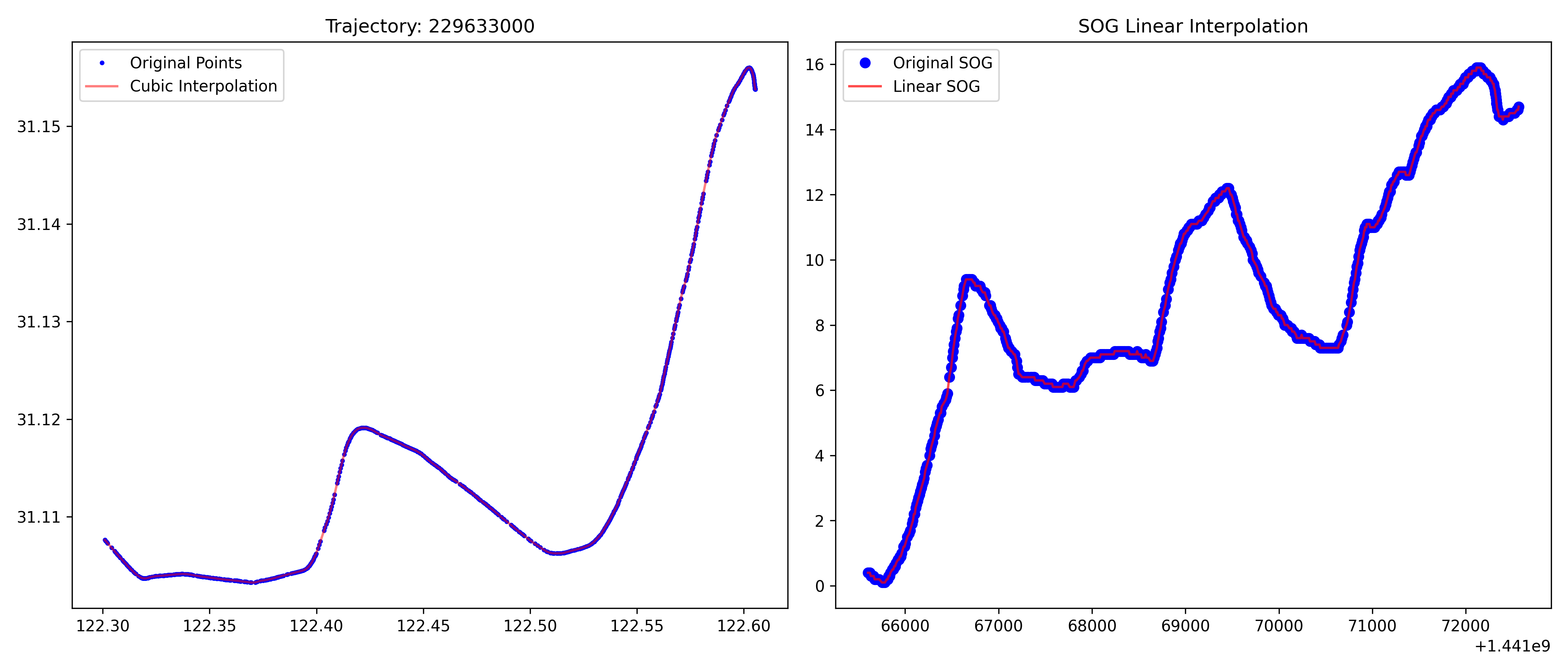

Supplement: S1 File — (ZIP) [file pone.0342781.s001.zip › data/interpolation/shipid_229633000_plot.png]

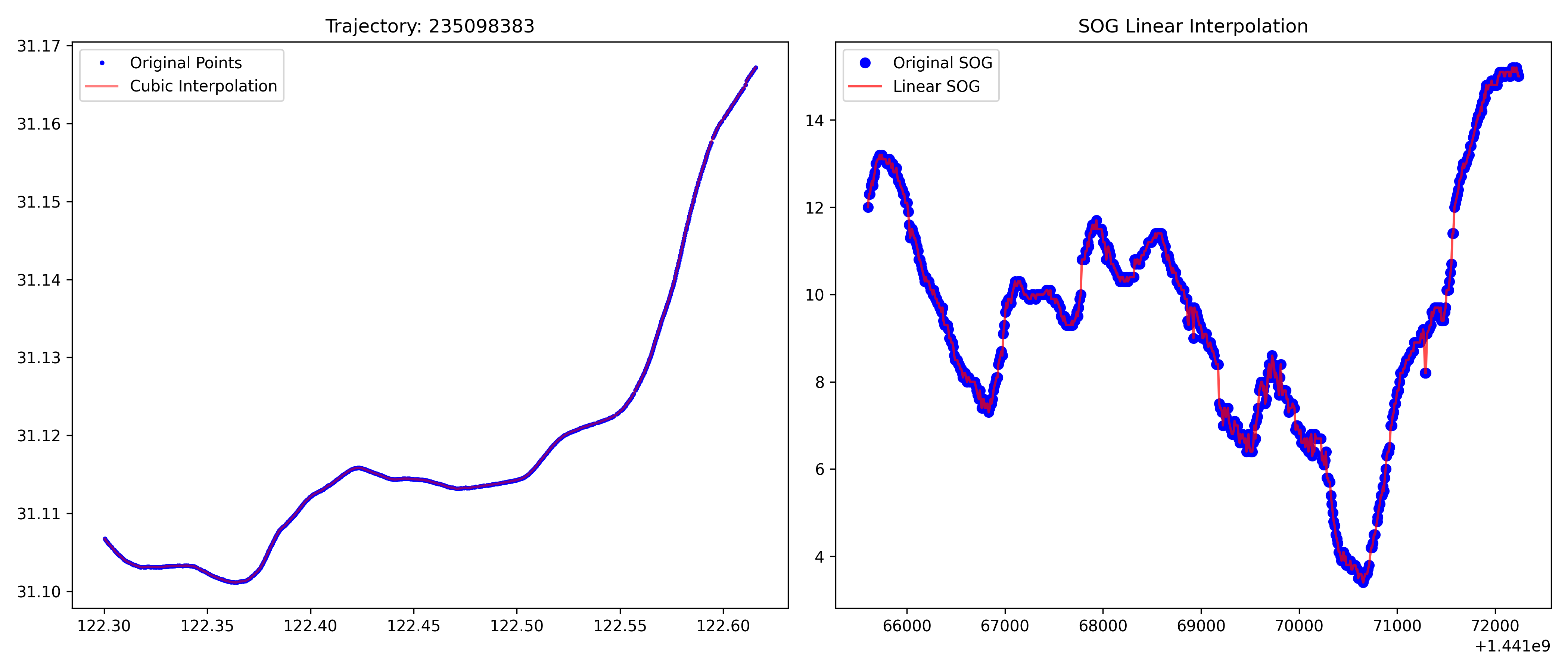

Supplement: S1 File — (ZIP) [file pone.0342781.s001.zip › data/interpolation/shipid_235098383_plot.png]

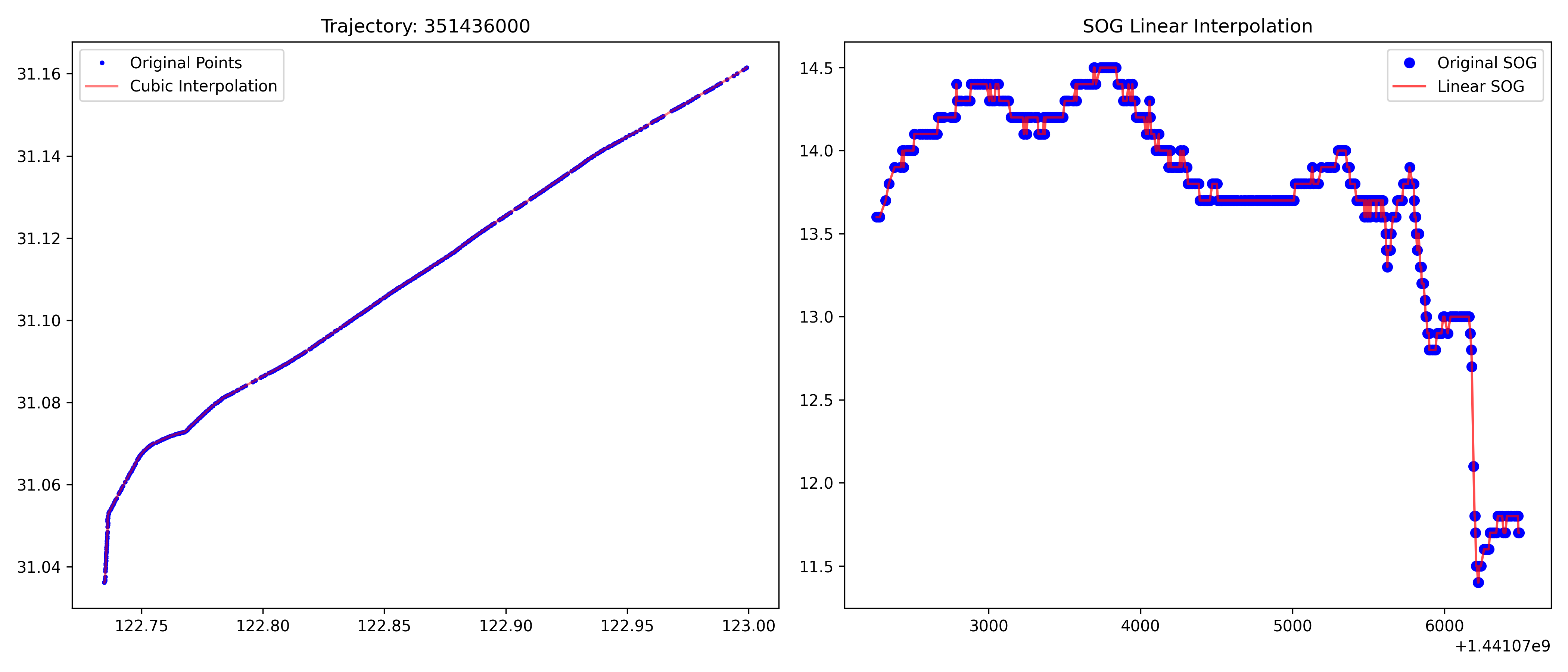

Supplement: S1 File — (ZIP) [file pone.0342781.s001.zip › data/interpolation/shipid_351436000_plot.png]

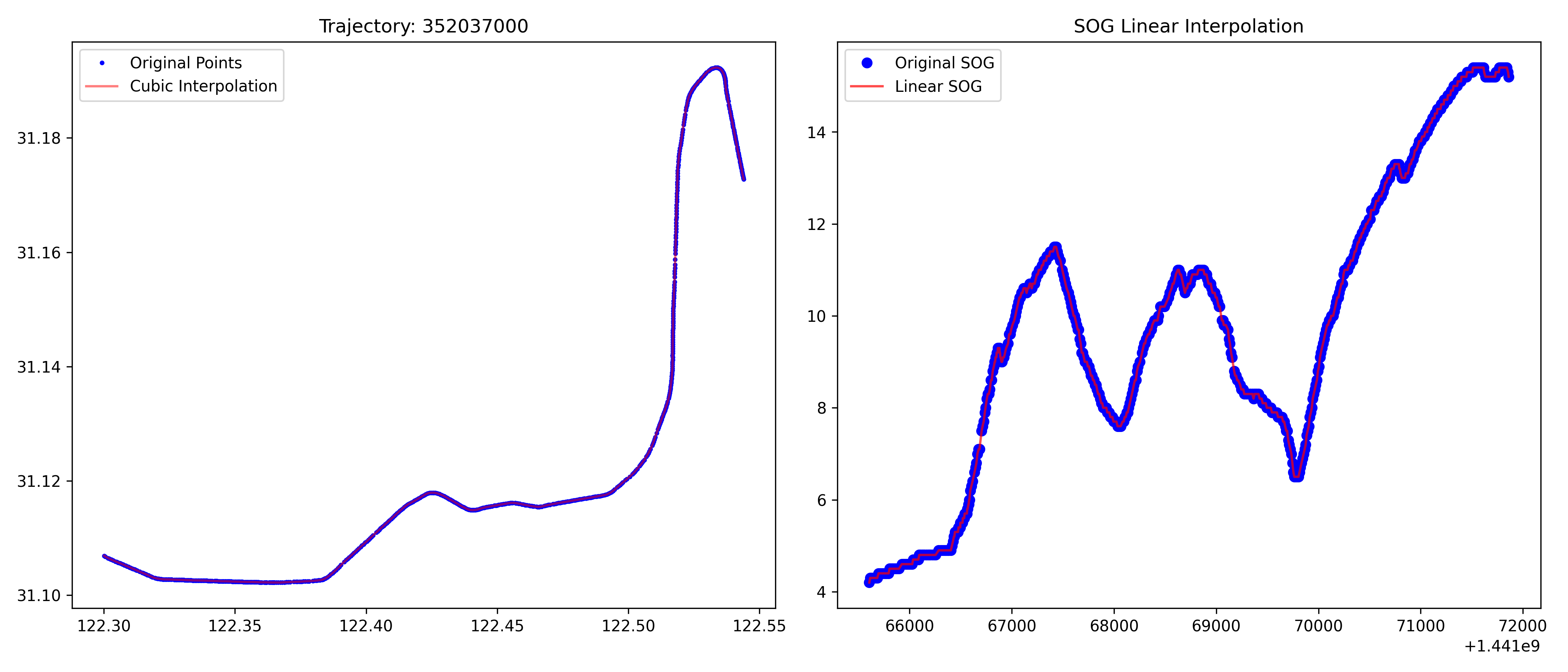

Supplement: S1 File — (ZIP) [file pone.0342781.s001.zip › data/interpolation/shipid_352037000_plot.png]

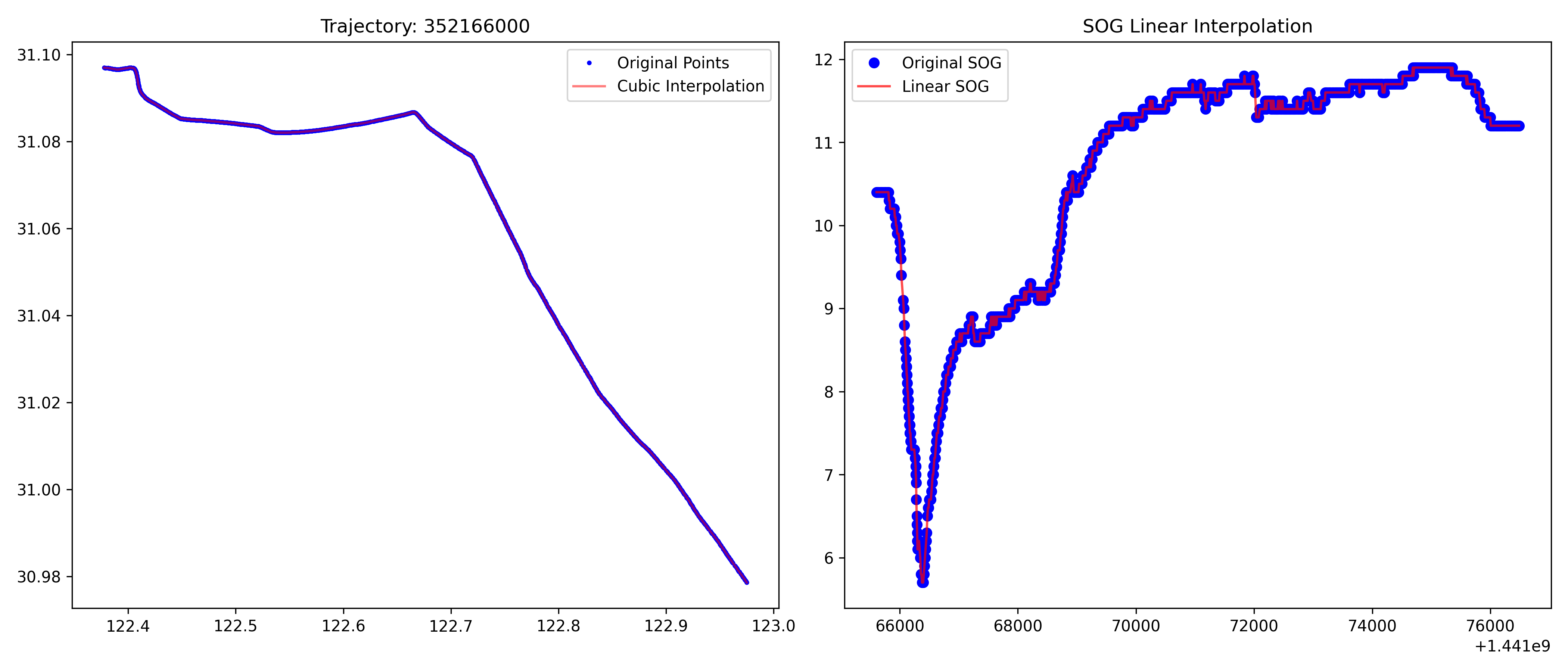

Supplement: S1 File — (ZIP) [file pone.0342781.s001.zip › data/interpolation/shipid_352166000_plot.png]

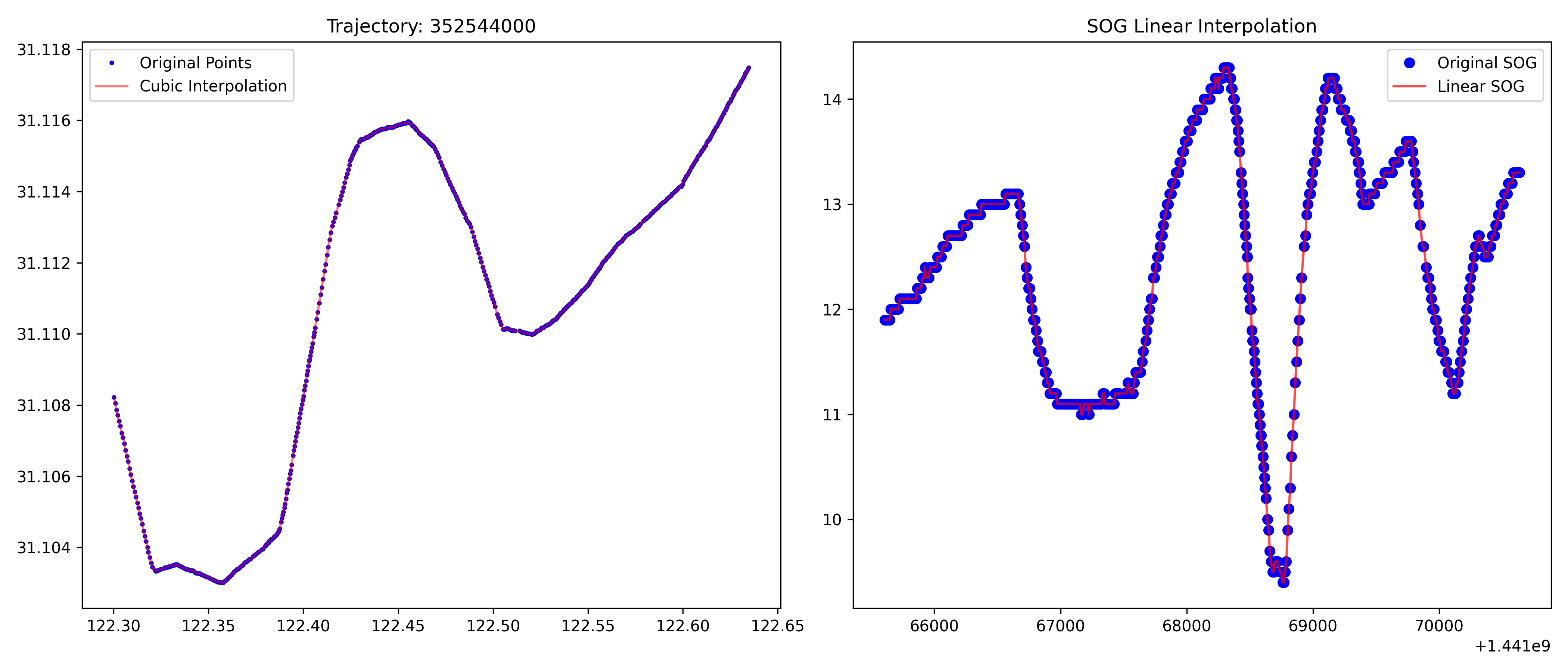

Supplement: S1 File — (ZIP) [file pone.0342781.s001.zip › data/interpolation/shipid_352544000_plot.png]

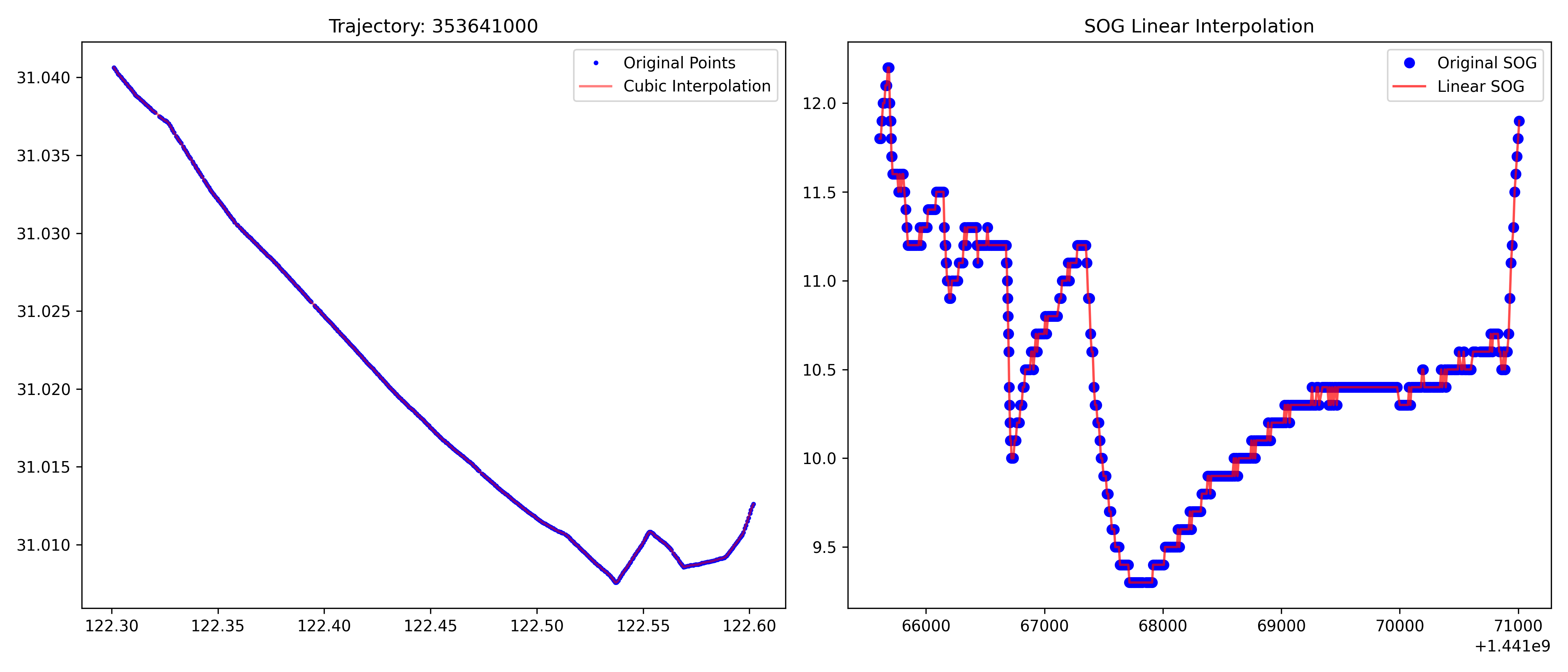

Supplement: S1 File — (ZIP) [file pone.0342781.s001.zip › data/interpolation/shipid_353641000_plot.png]

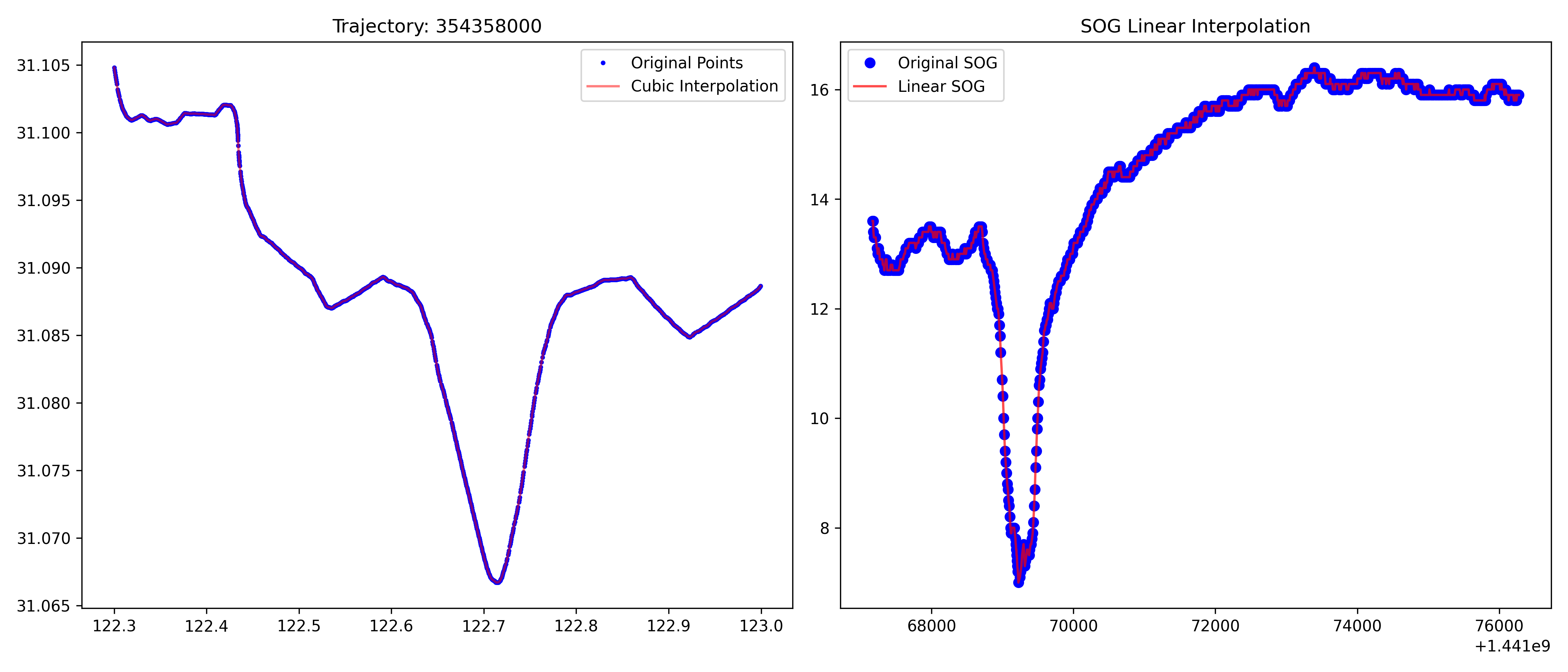

Supplement: S1 File — (ZIP) [file pone.0342781.s001.zip › data/interpolation/shipid_354358000_plot.png]

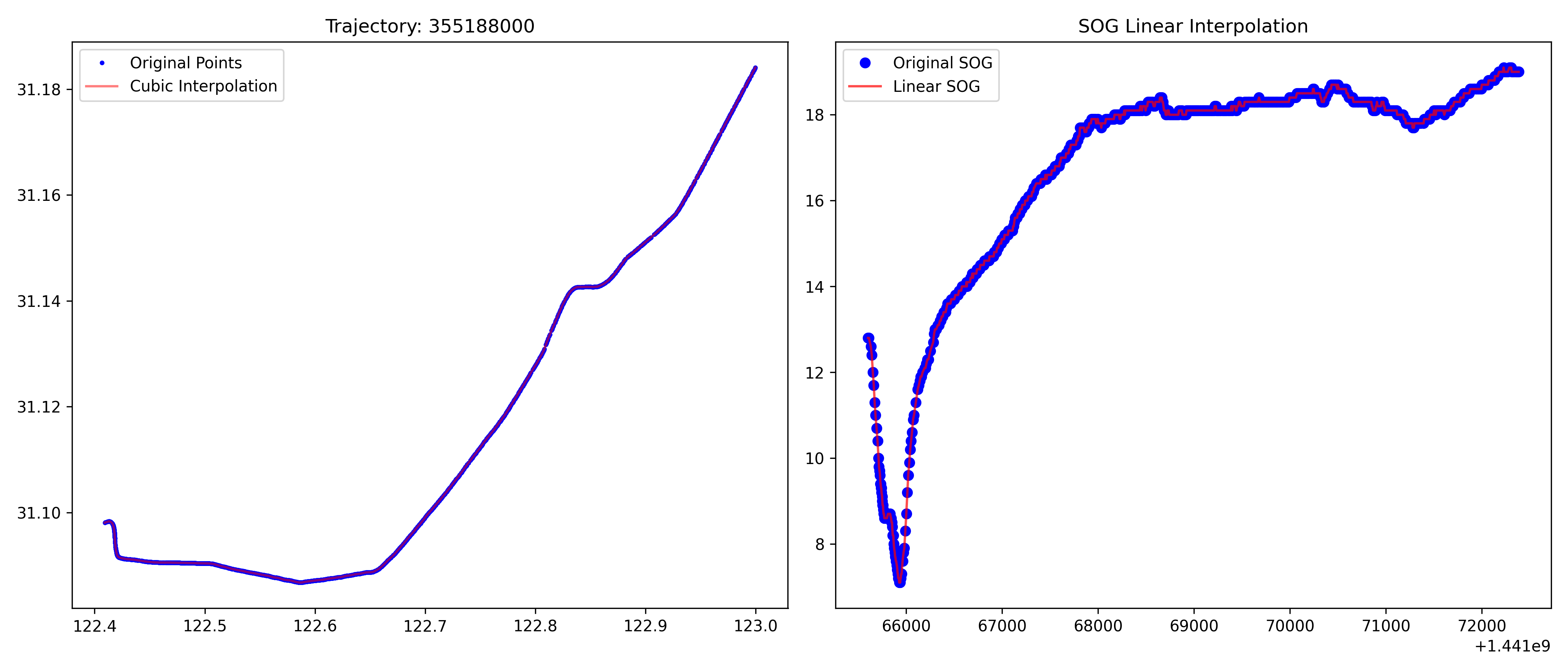

Supplement: S1 File — (ZIP) [file pone.0342781.s001.zip › data/interpolation/shipid_355188000_plot.png]

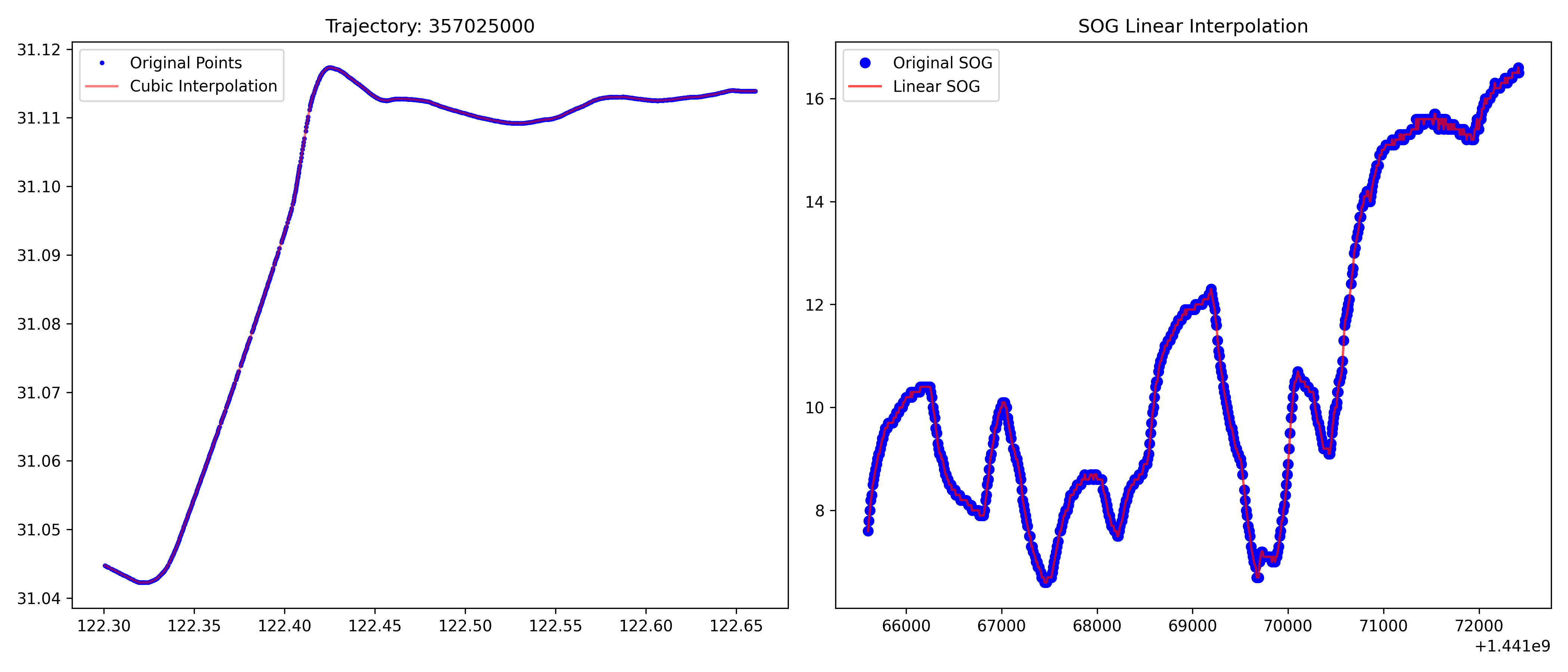

Supplement: S1 File — (ZIP) [file pone.0342781.s001.zip › data/interpolation/shipid_357025000_plot.png]

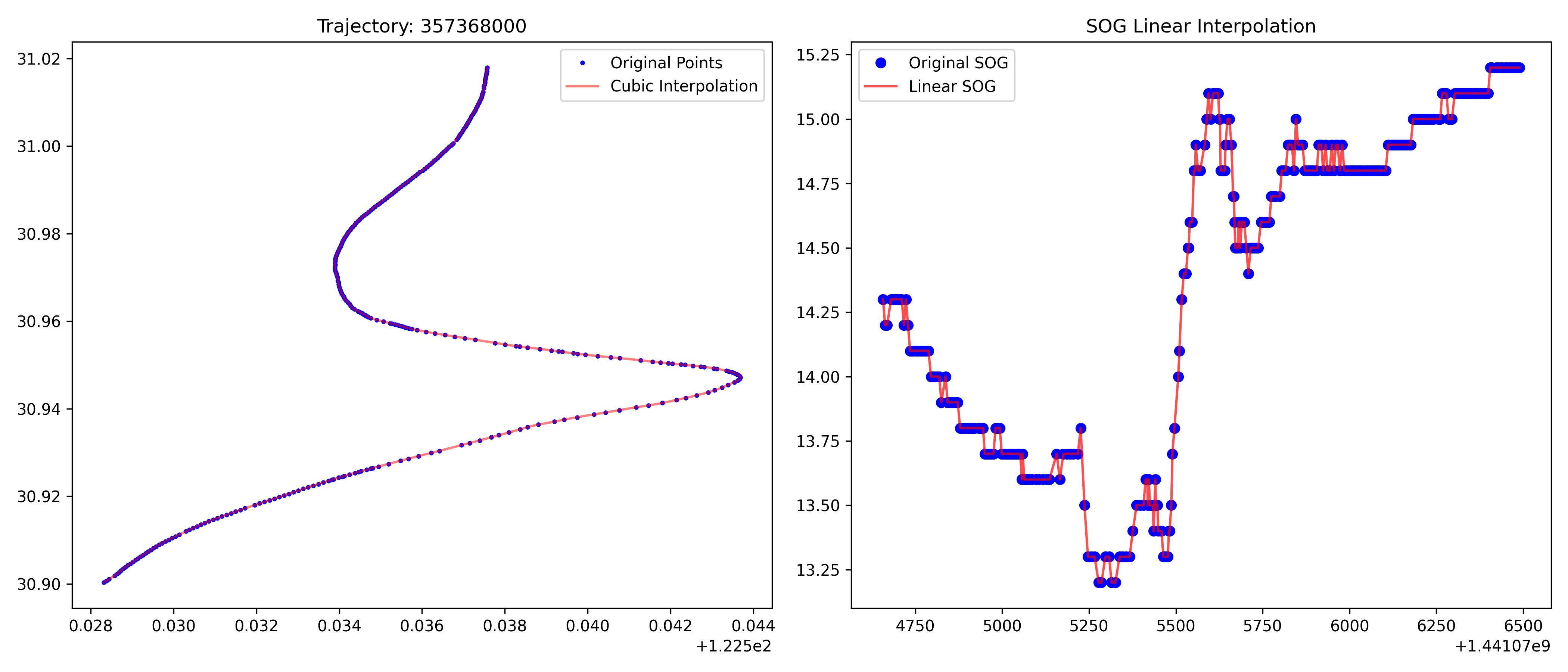

Supplement: S1 File — (ZIP) [file pone.0342781.s001.zip › data/interpolation/shipid_357368000_plot.png]

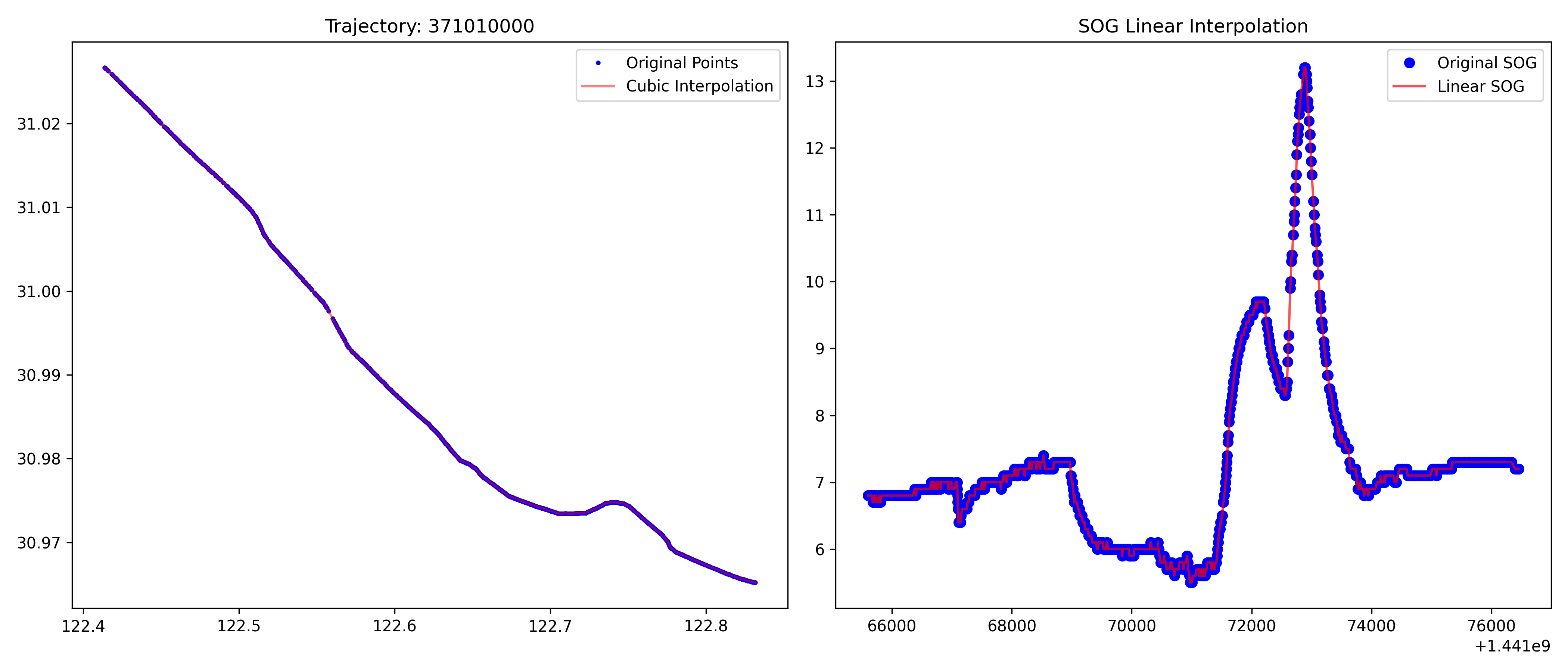

Supplement: S1 File — (ZIP) [file pone.0342781.s001.zip › data/interpolation/shipid_371010000_plot.png]

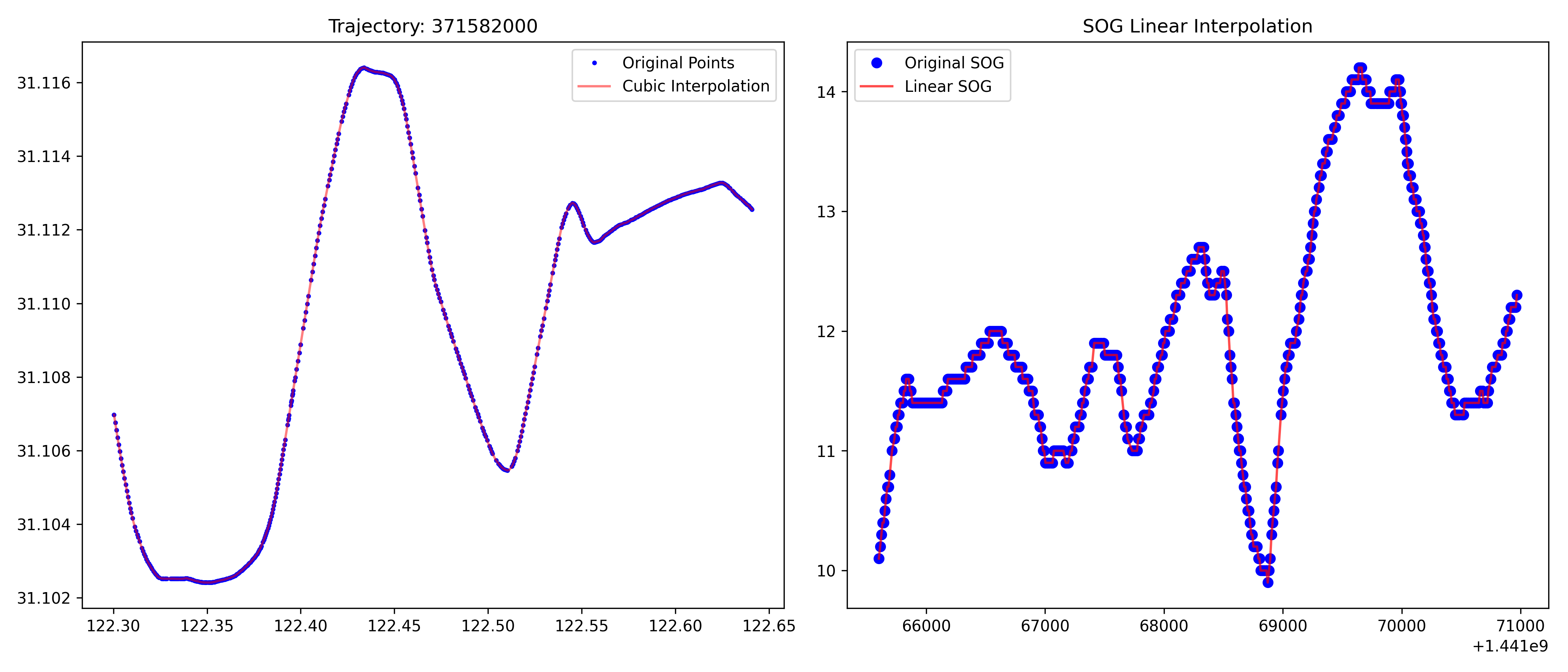

Supplement: S1 File — (ZIP) [file pone.0342781.s001.zip › data/interpolation/shipid_371582000_plot.png]

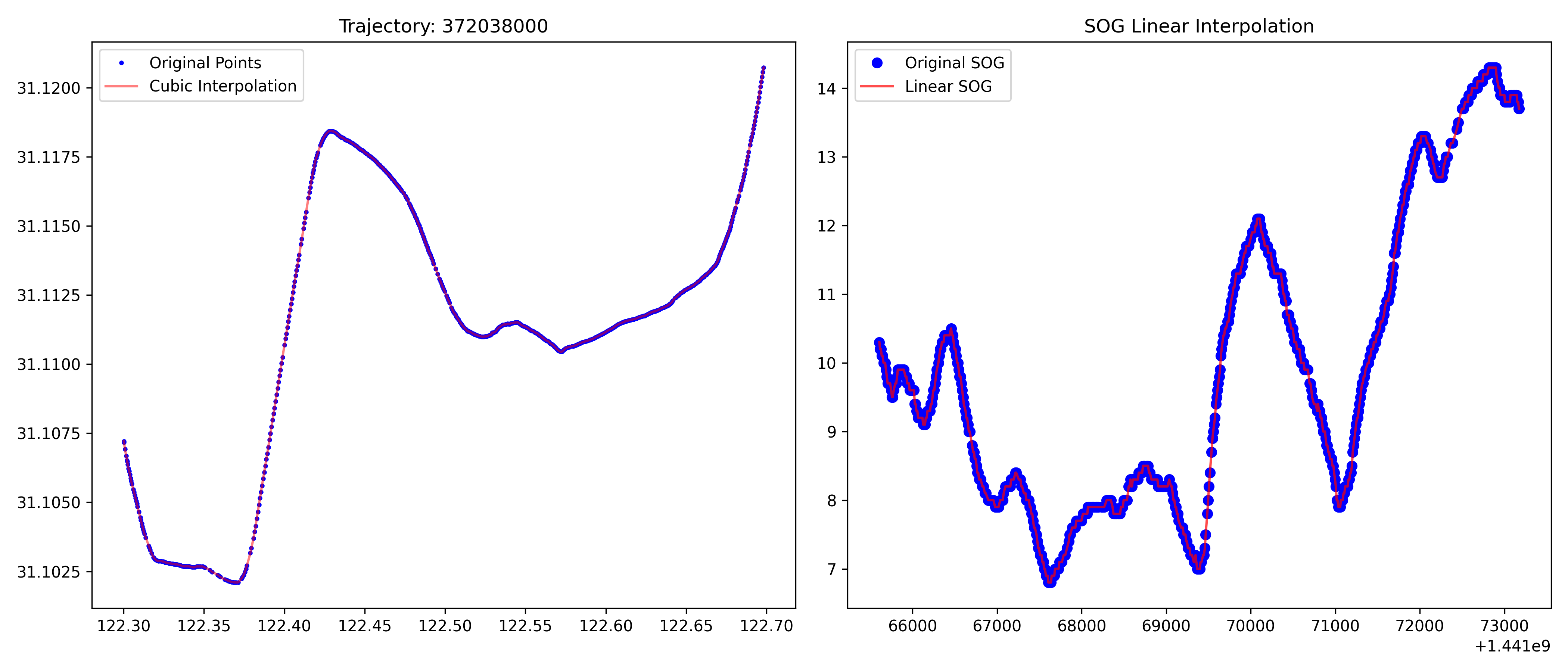

Supplement: S1 File — (ZIP) [file pone.0342781.s001.zip › data/interpolation/shipid_372038000_plot.png]

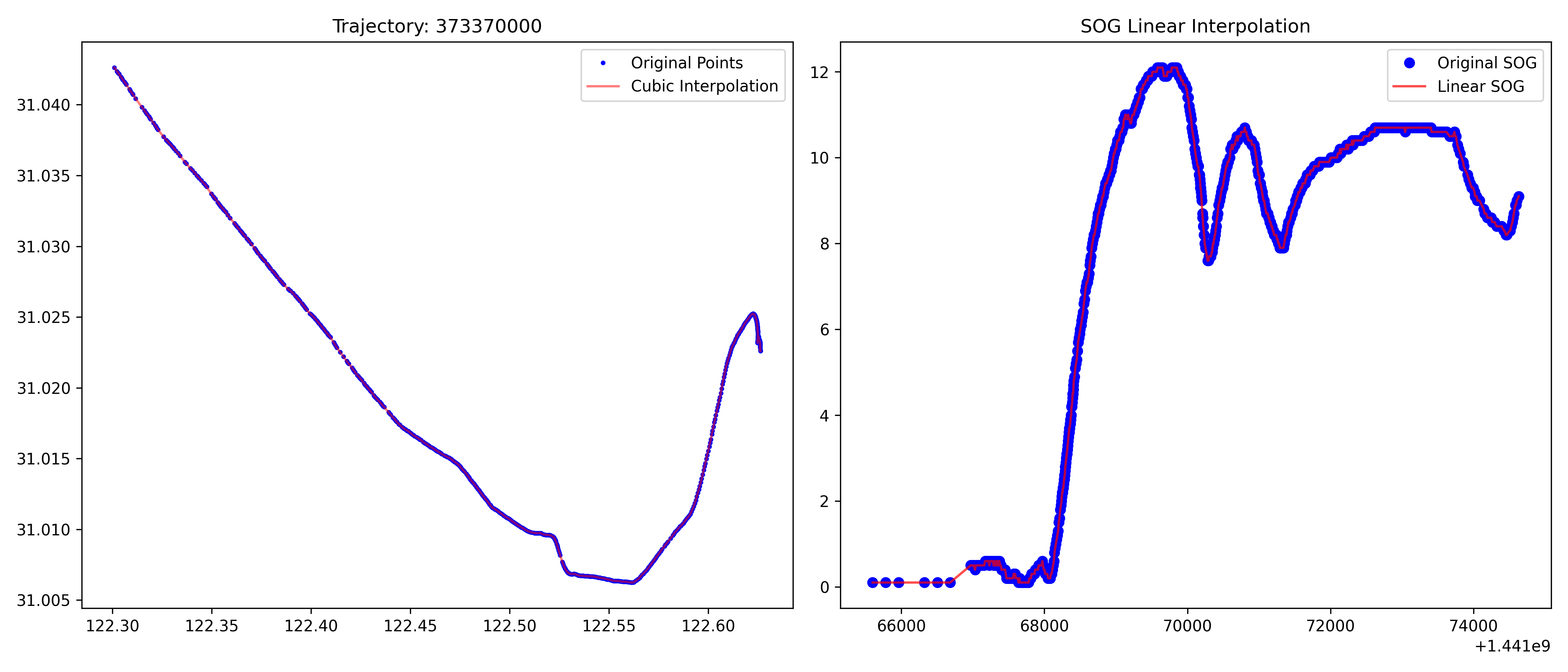

Supplement: S1 File — (ZIP) [file pone.0342781.s001.zip › data/interpolation/shipid_373370000_plot.png]

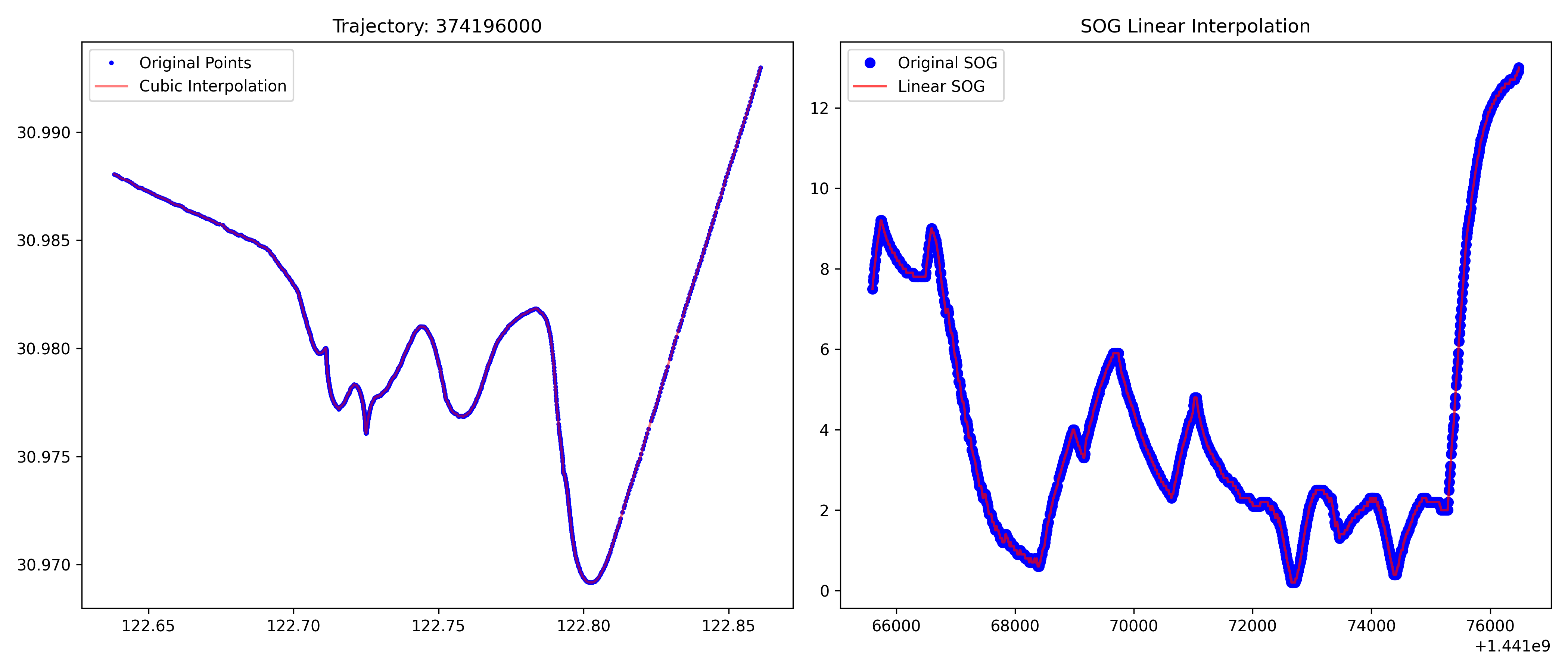

Supplement: S1 File — (ZIP) [file pone.0342781.s001.zip › data/interpolation/shipid_374196000_plot.png]

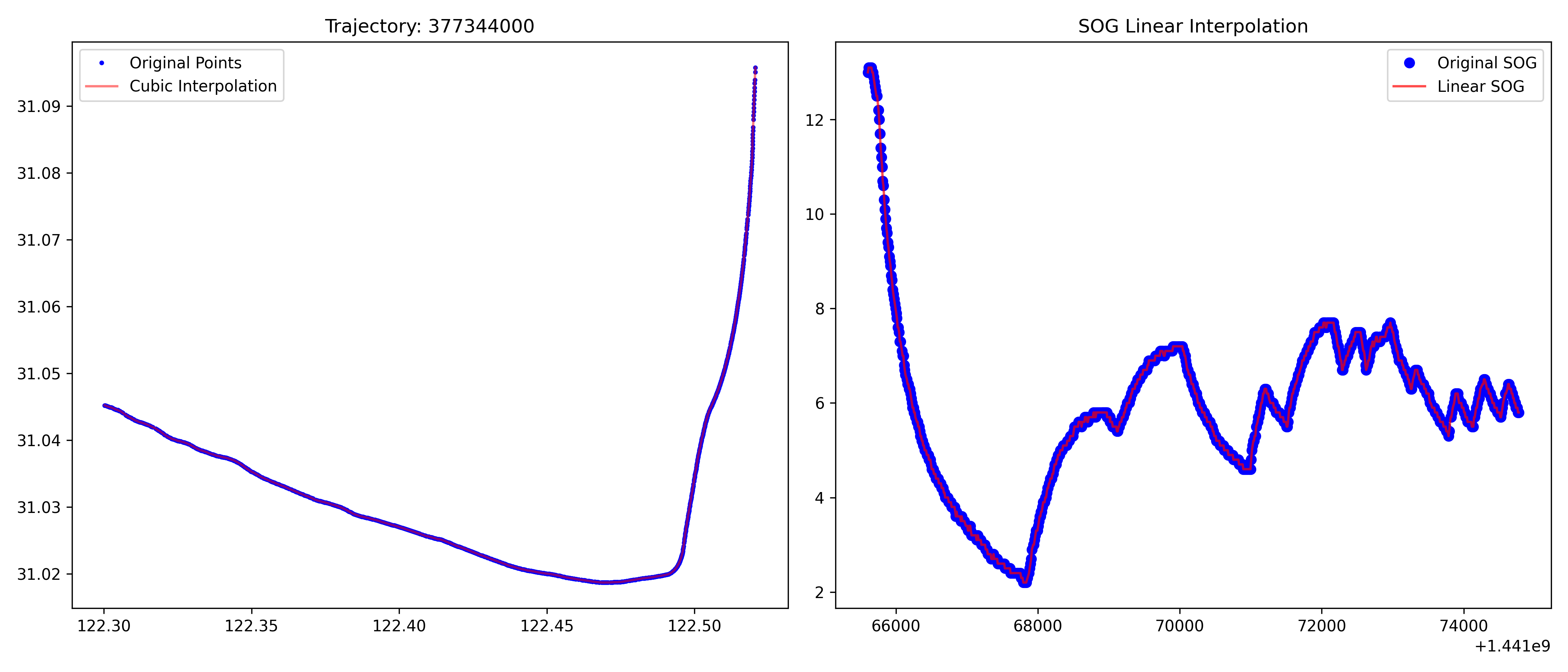

Supplement: S1 File — (ZIP) [file pone.0342781.s001.zip › data/interpolation/shipid_377344000_plot.png]

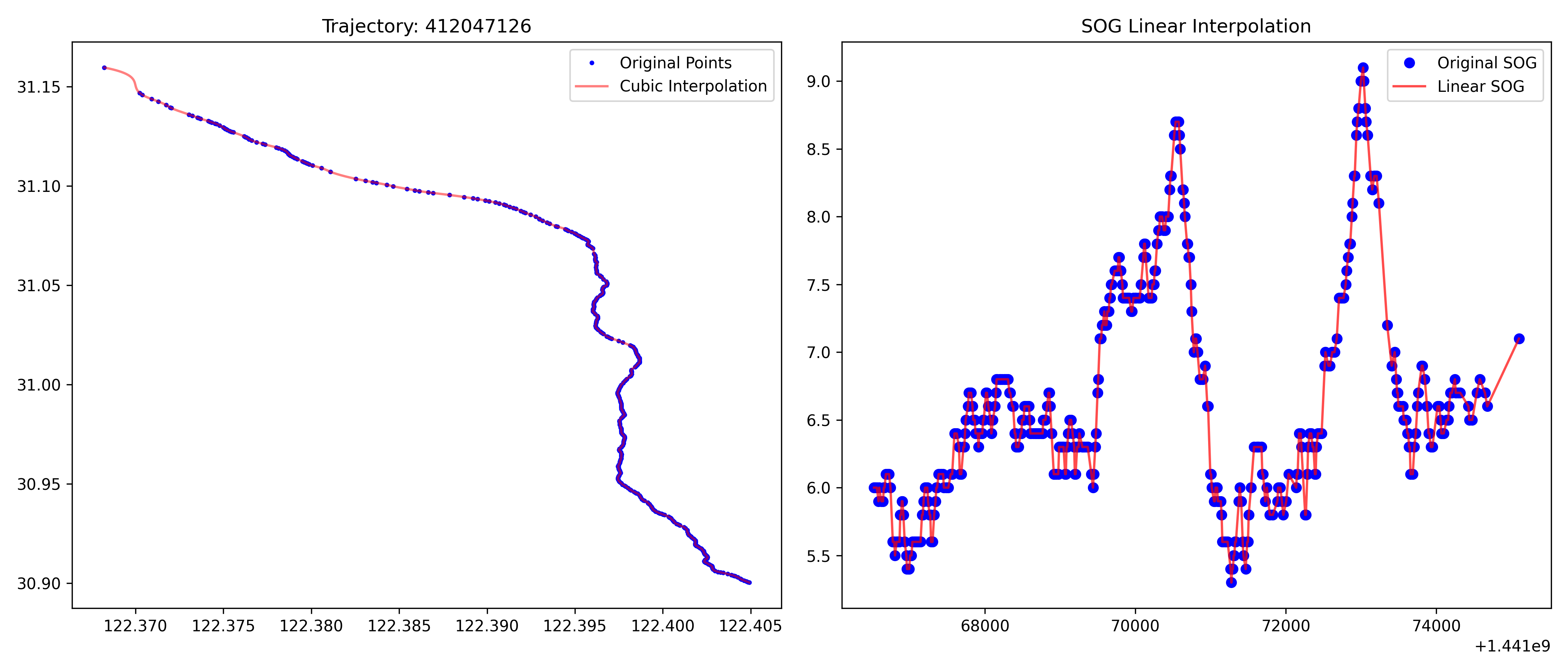

Supplement: S1 File — (ZIP) [file pone.0342781.s001.zip › data/interpolation/shipid_412047126_plot.png]

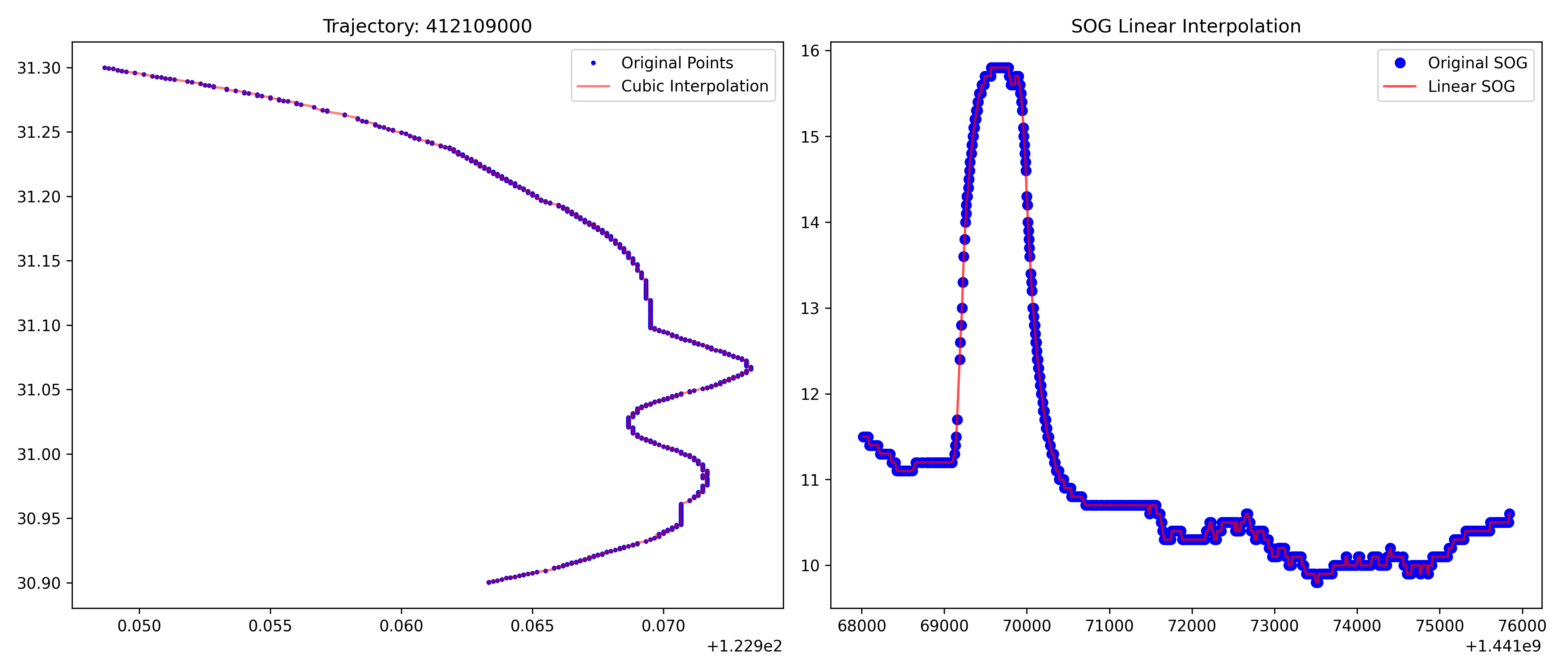

Supplement: S1 File — (ZIP) [file pone.0342781.s001.zip › data/interpolation/shipid_412109000_plot.png]

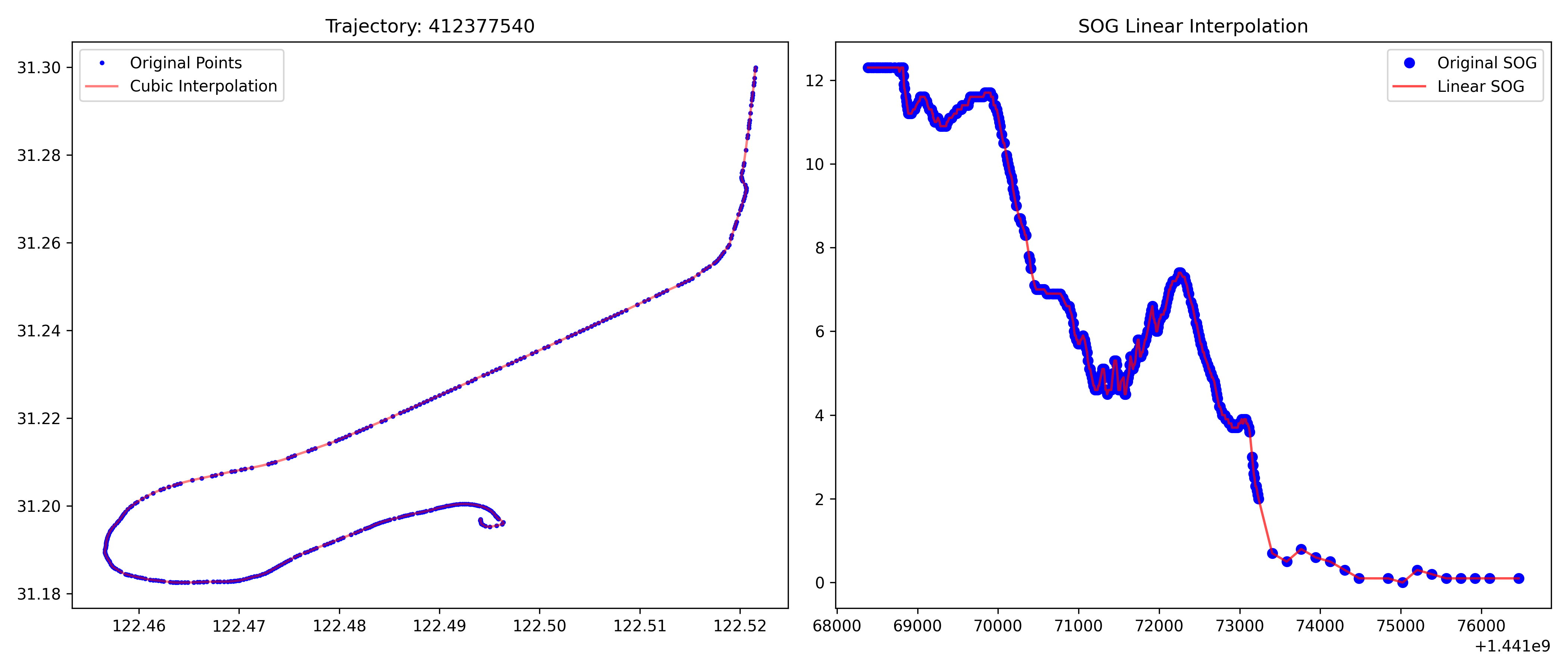

Supplement: S1 File — (ZIP) [file pone.0342781.s001.zip › data/interpolation/shipid_412377540_plot.png]

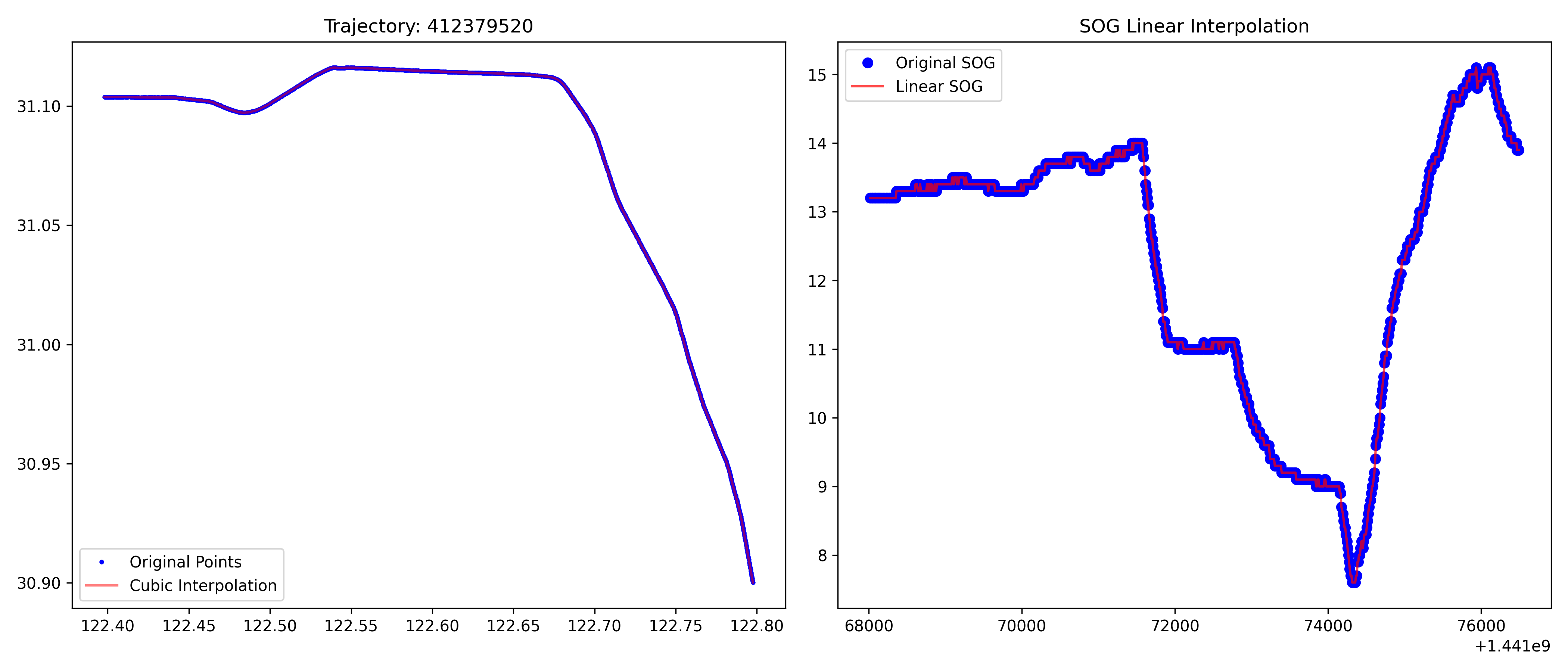

Supplement: S1 File — (ZIP) [file pone.0342781.s001.zip › data/interpolation/shipid_412379520_plot.png]

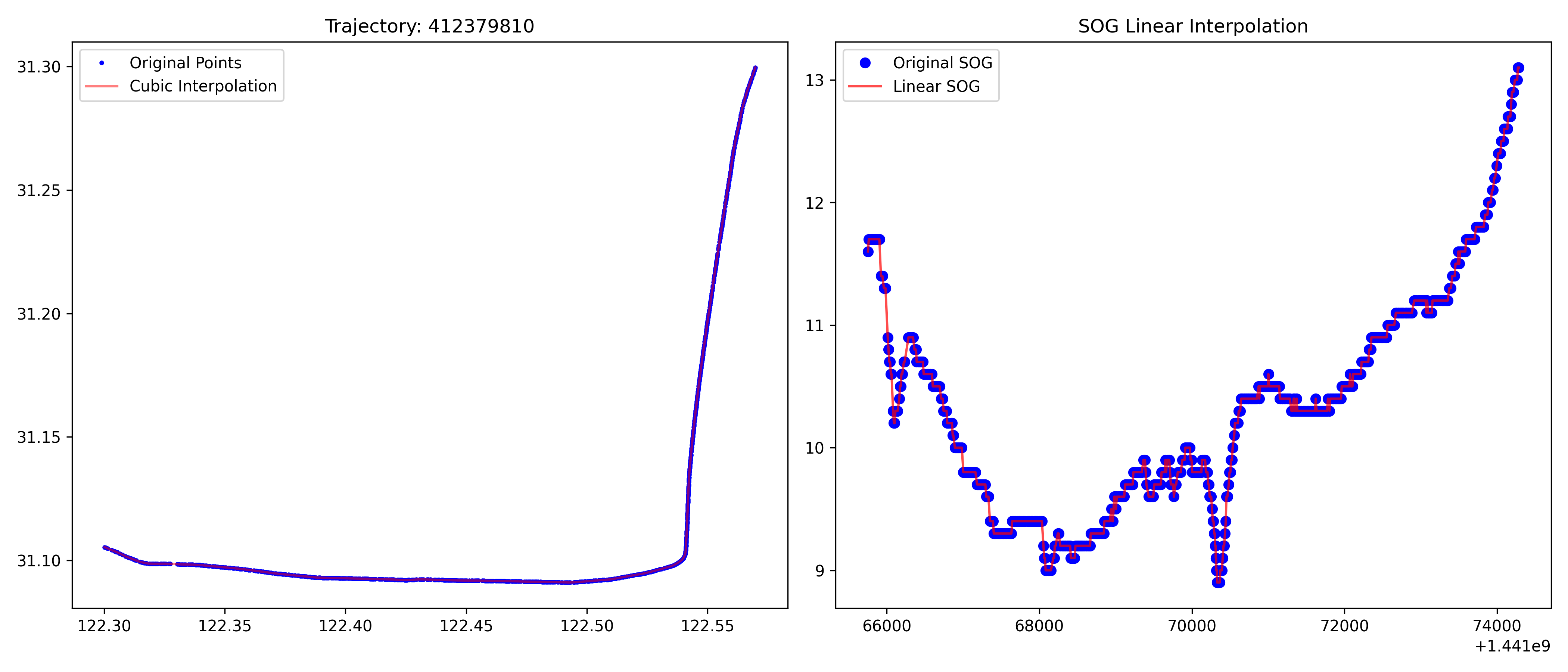

Supplement: S1 File — (ZIP) [file pone.0342781.s001.zip › data/interpolation/shipid_412379810_plot.png]

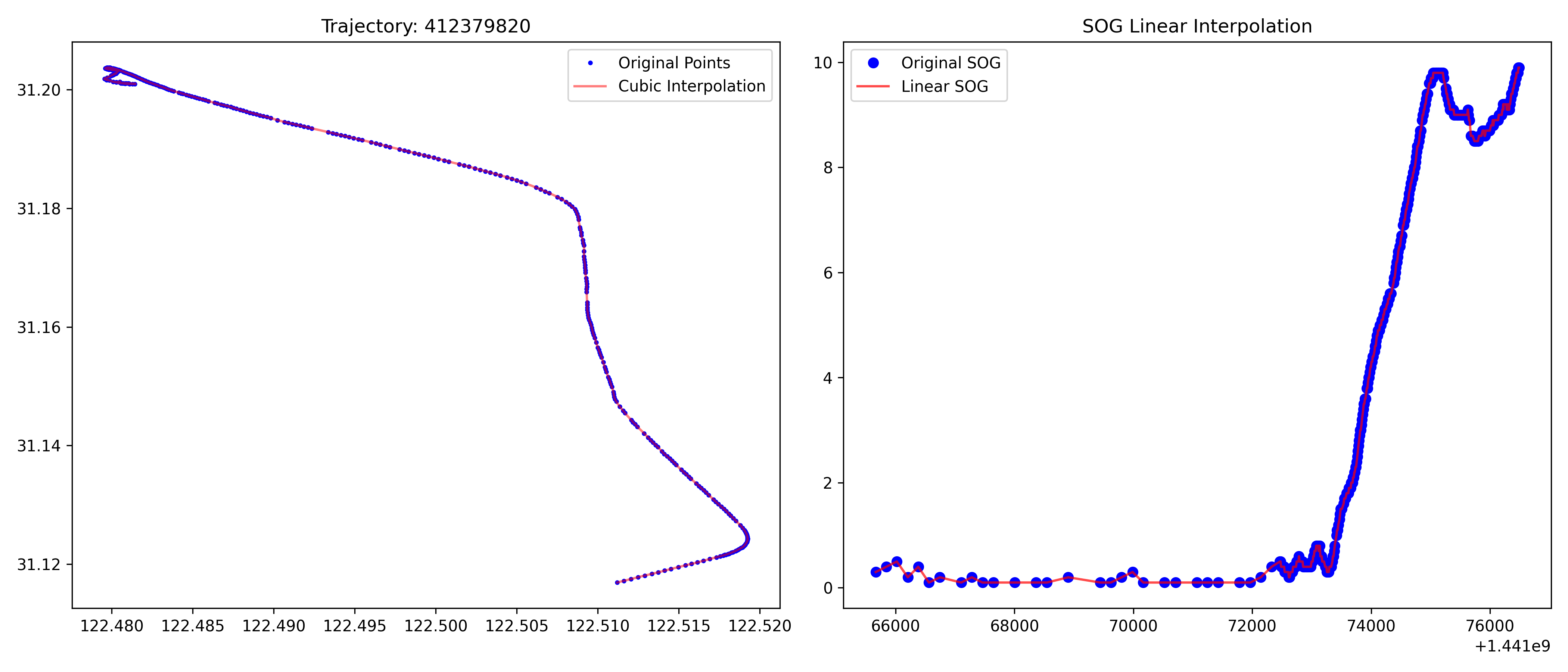

Supplement: S1 File — (ZIP) [file pone.0342781.s001.zip › data/interpolation/shipid_412379820_plot.png]

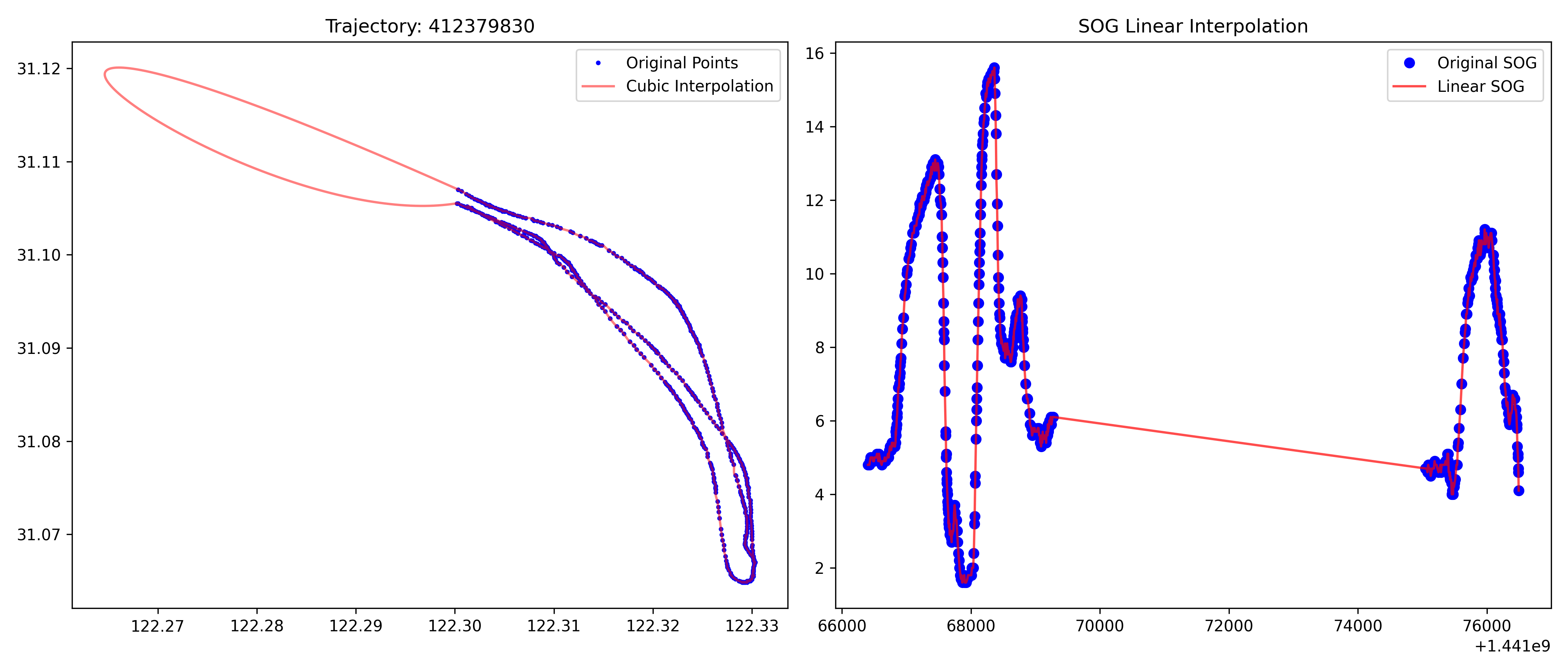

Supplement: S1 File — (ZIP) [file pone.0342781.s001.zip › data/interpolation/shipid_412379830_plot.png]

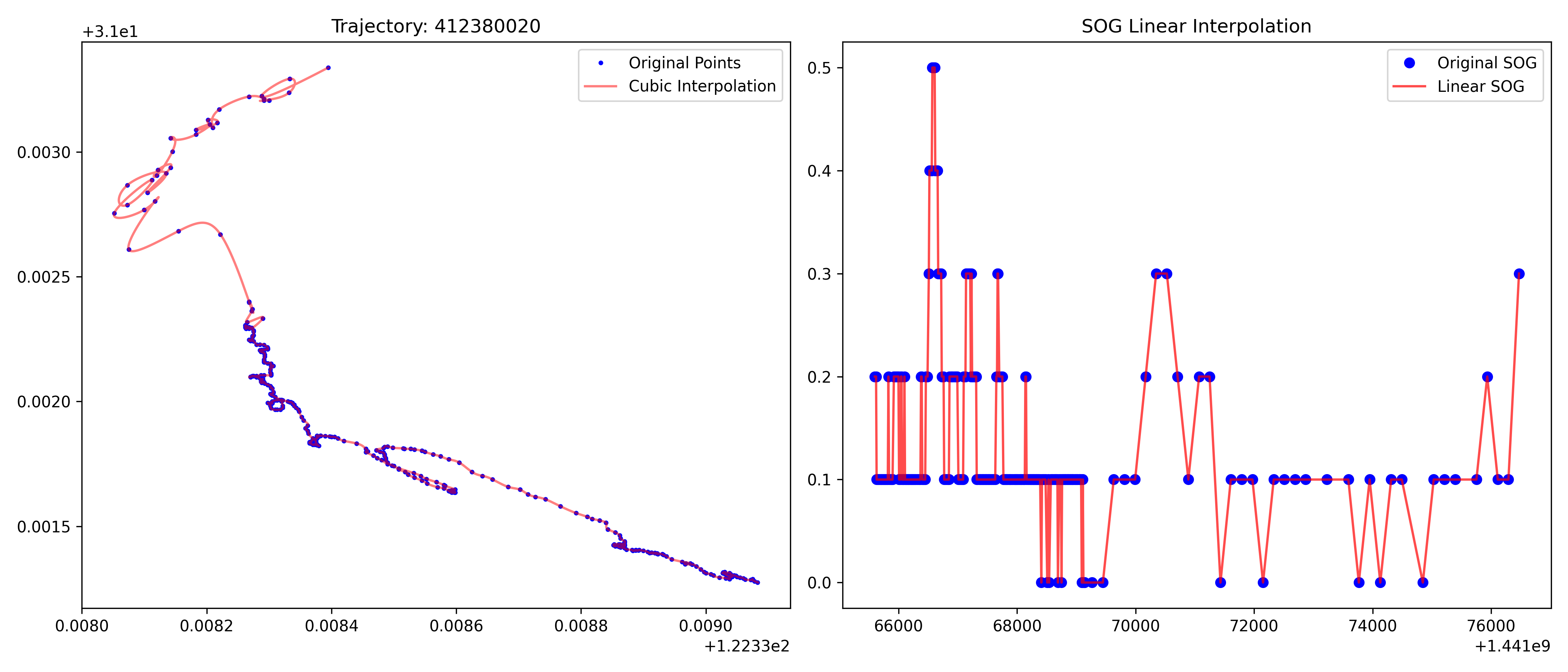

Supplement: S1 File — (ZIP) [file pone.0342781.s001.zip › data/interpolation/shipid_412380020_plot.png]

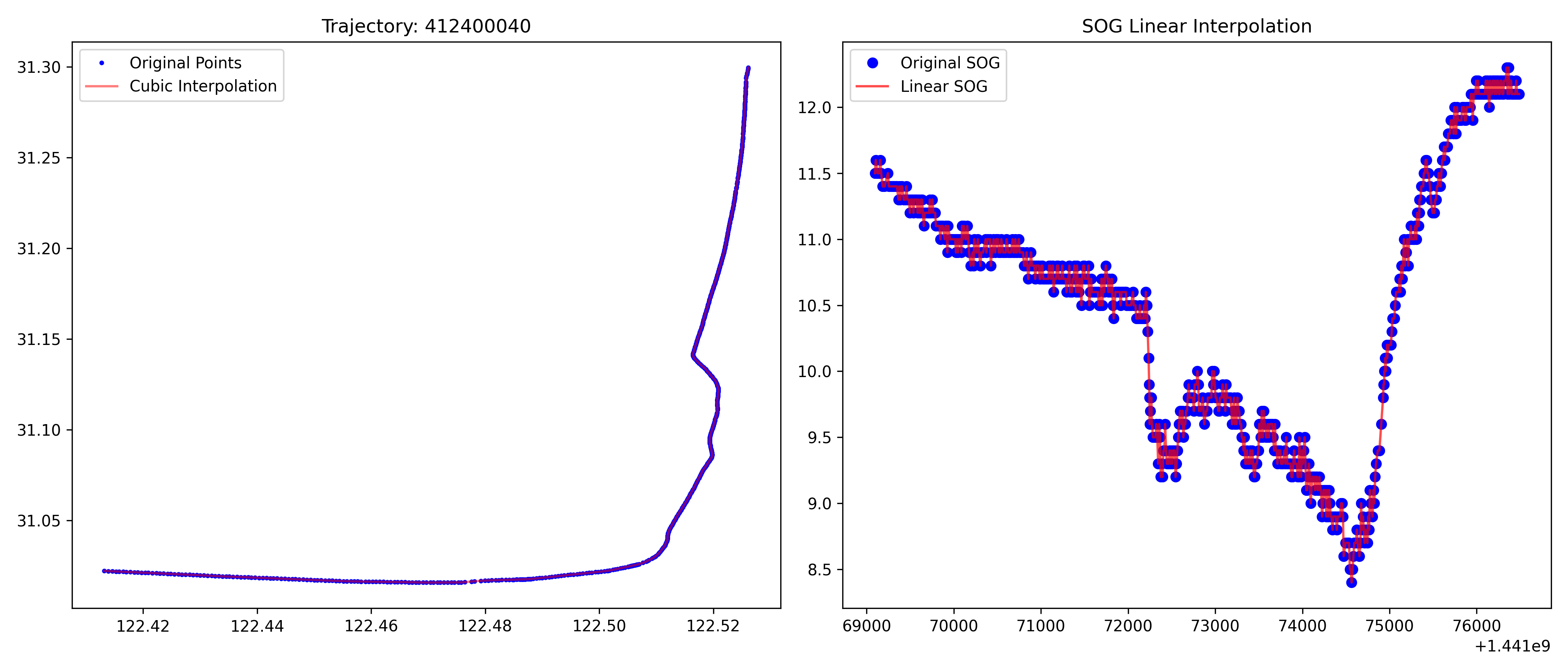

Supplement: S1 File — (ZIP) [file pone.0342781.s001.zip › data/interpolation/shipid_412400040_plot.png]

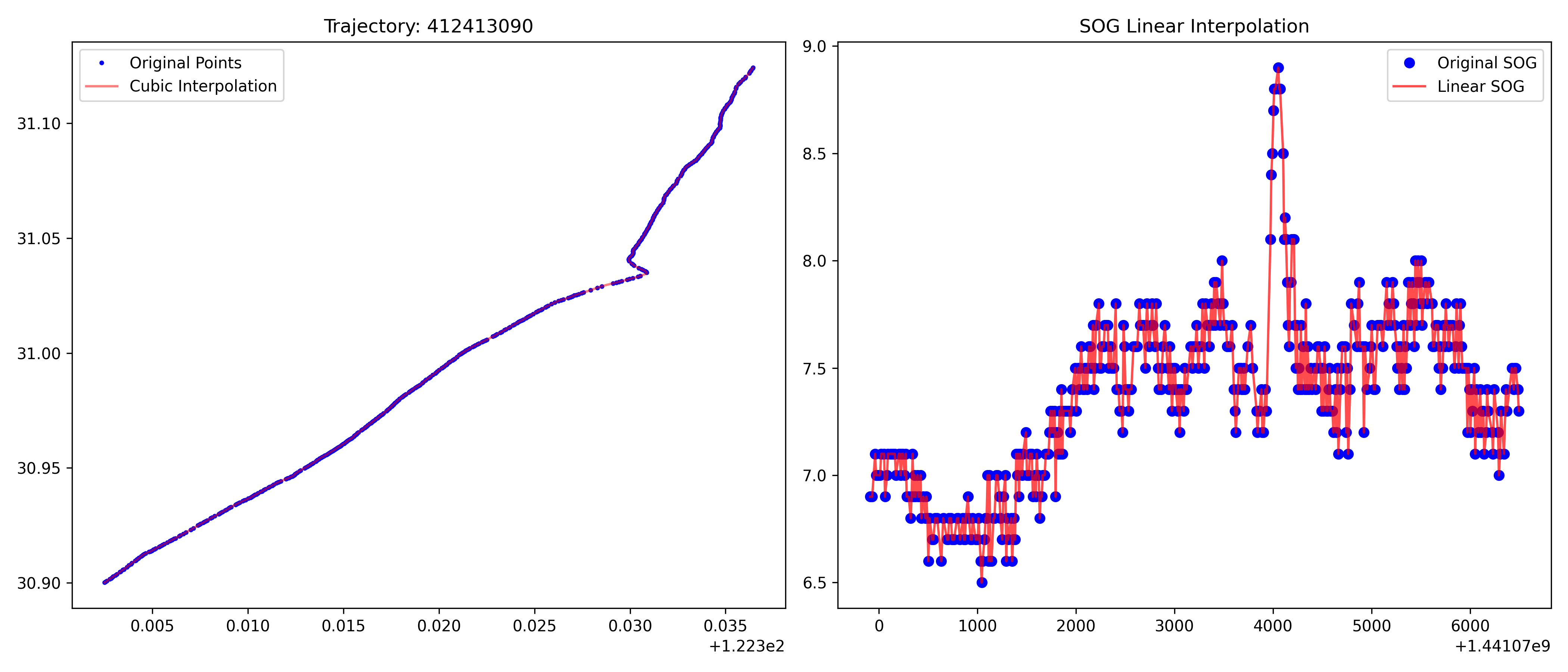

Supplement: S1 File — (ZIP) [file pone.0342781.s001.zip › data/interpolation/shipid_412413090_plot.png]

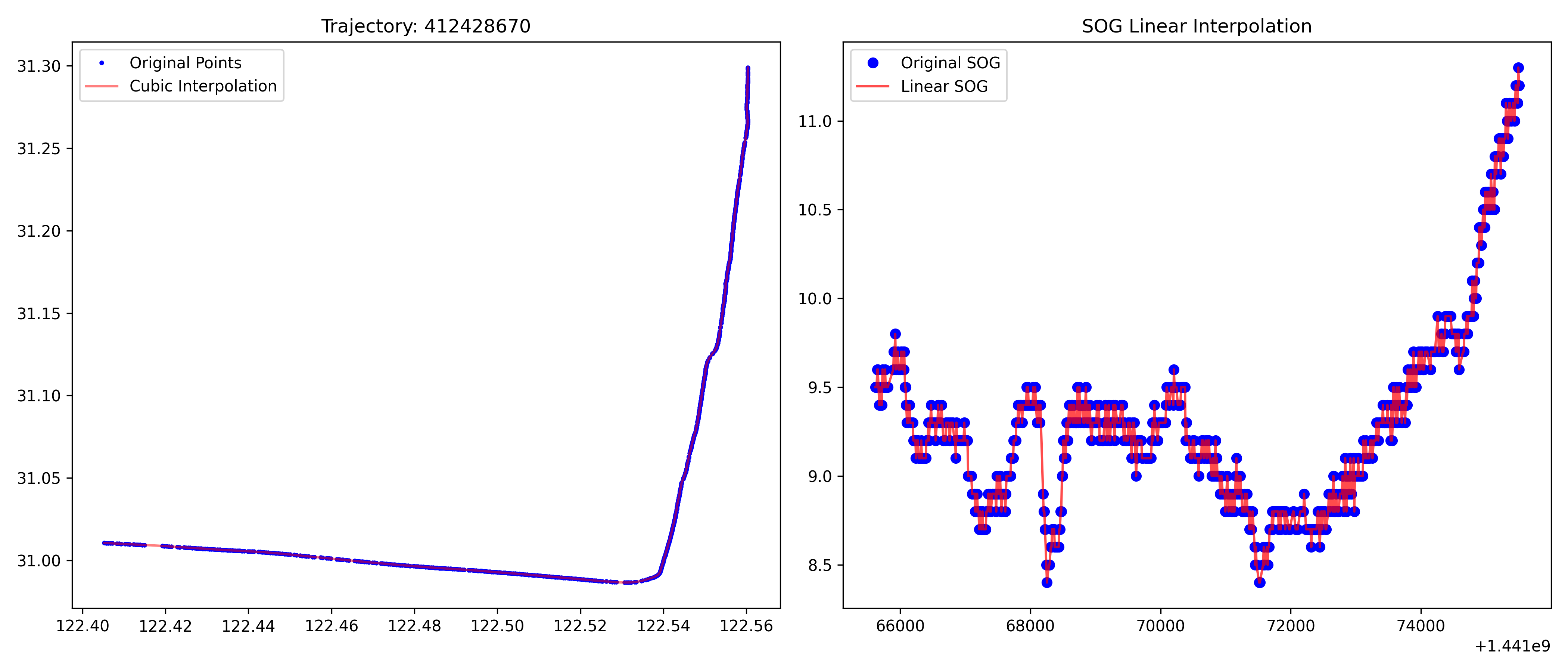

Supplement: S1 File — (ZIP) [file pone.0342781.s001.zip › data/interpolation/shipid_412428670_plot.png]

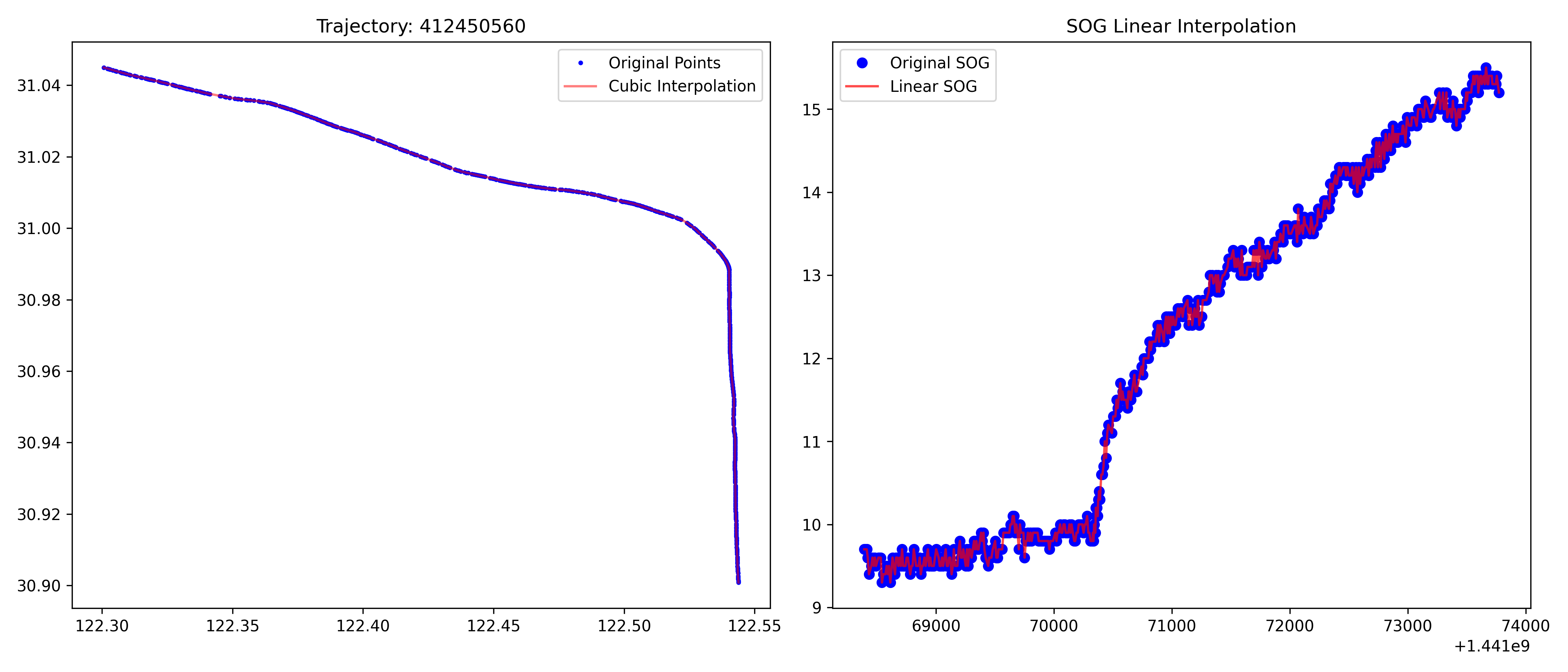

Supplement: S1 File — (ZIP) [file pone.0342781.s001.zip › data/interpolation/shipid_412450560_plot.png]

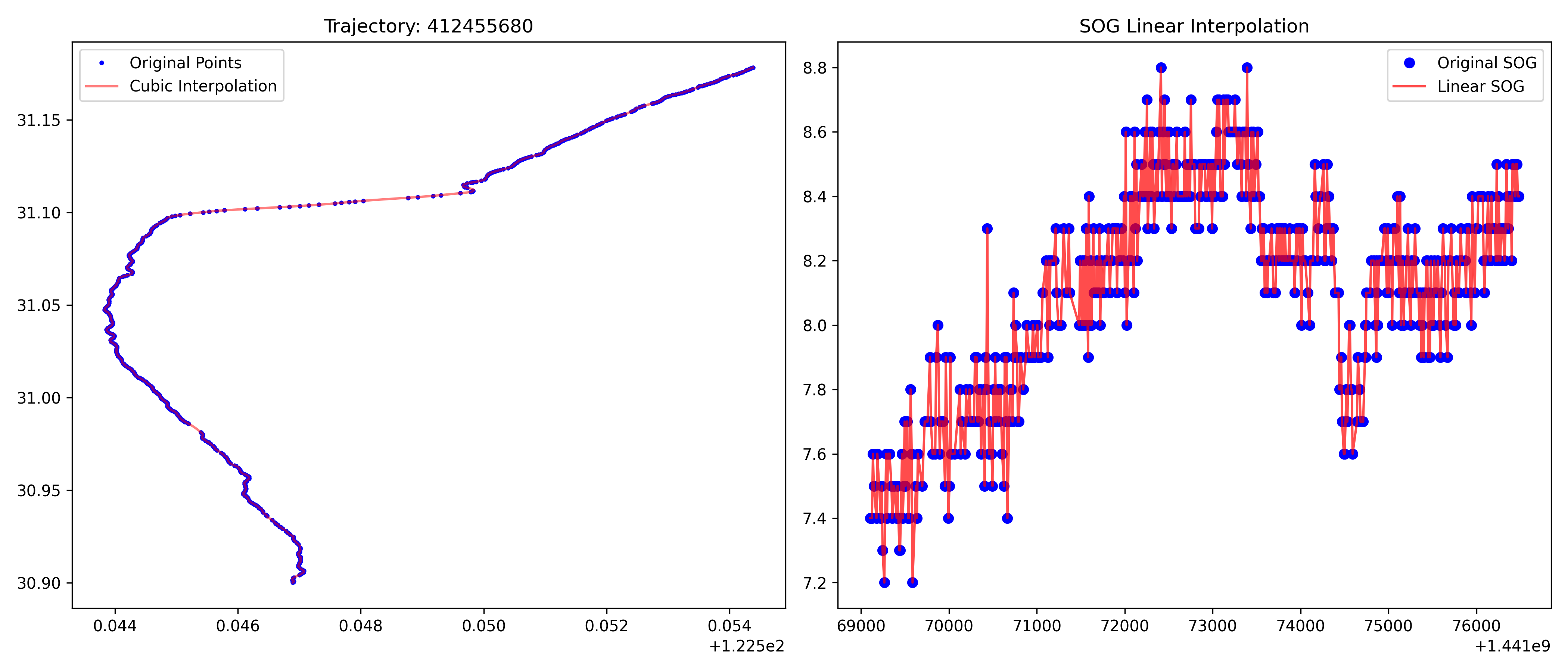

Supplement: S1 File — (ZIP) [file pone.0342781.s001.zip › data/interpolation/shipid_412455680_plot.png]

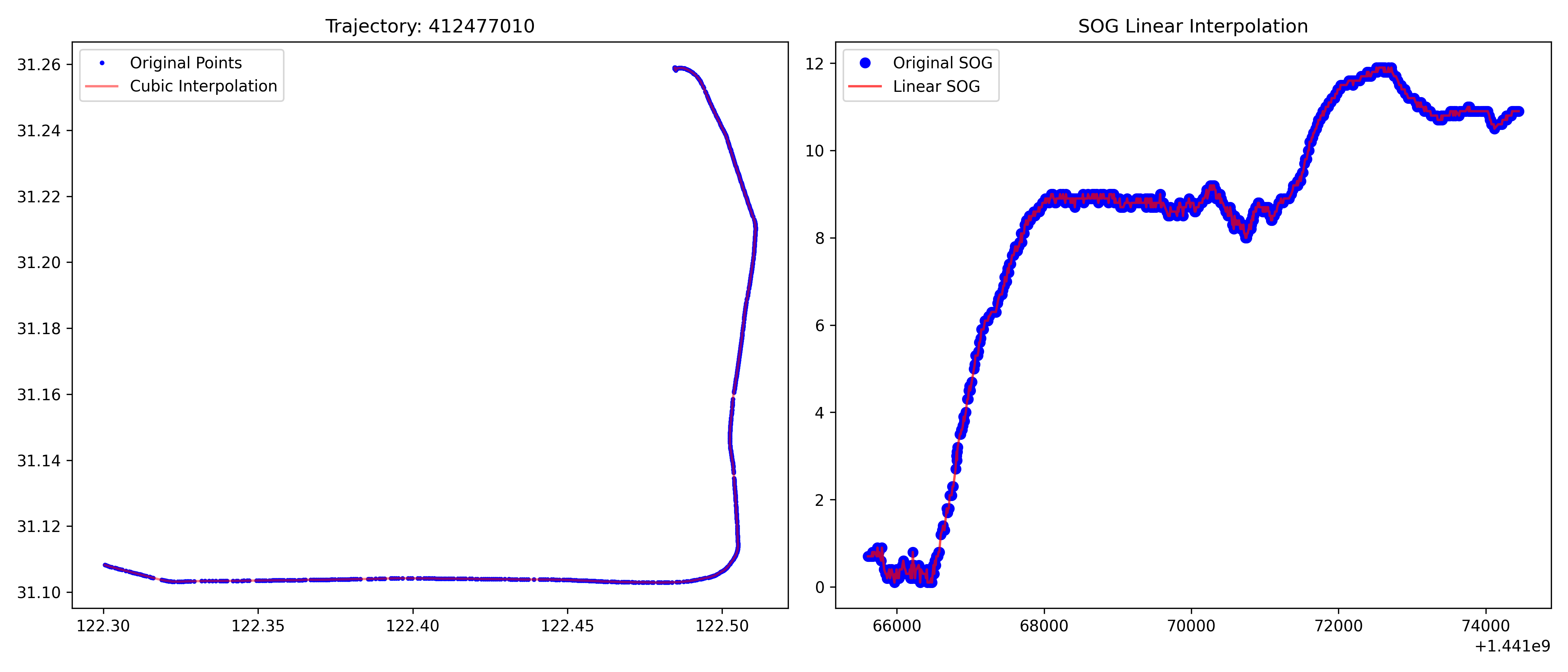

Supplement: S1 File — (ZIP) [file pone.0342781.s001.zip › data/interpolation/shipid_412477010_plot.png]

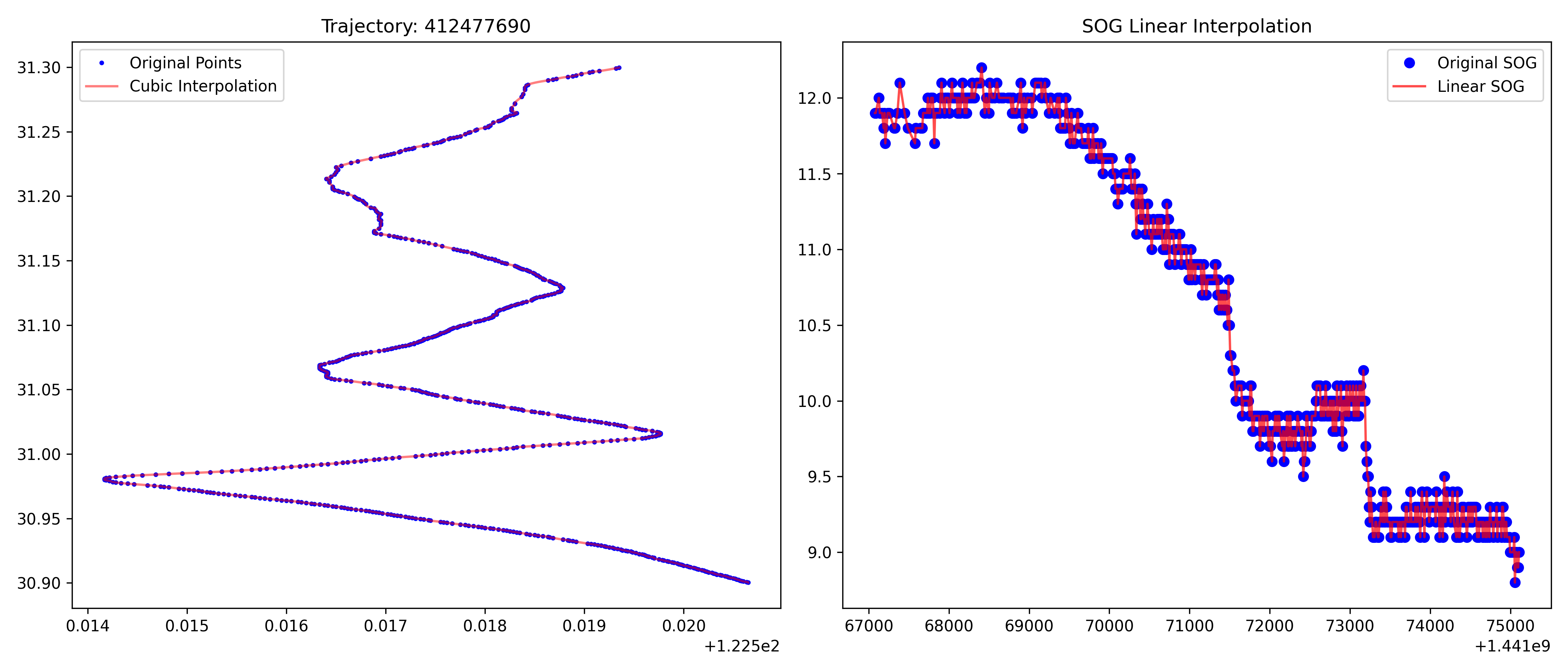

Supplement: S1 File — (ZIP) [file pone.0342781.s001.zip › data/interpolation/shipid_412477690_plot.png]

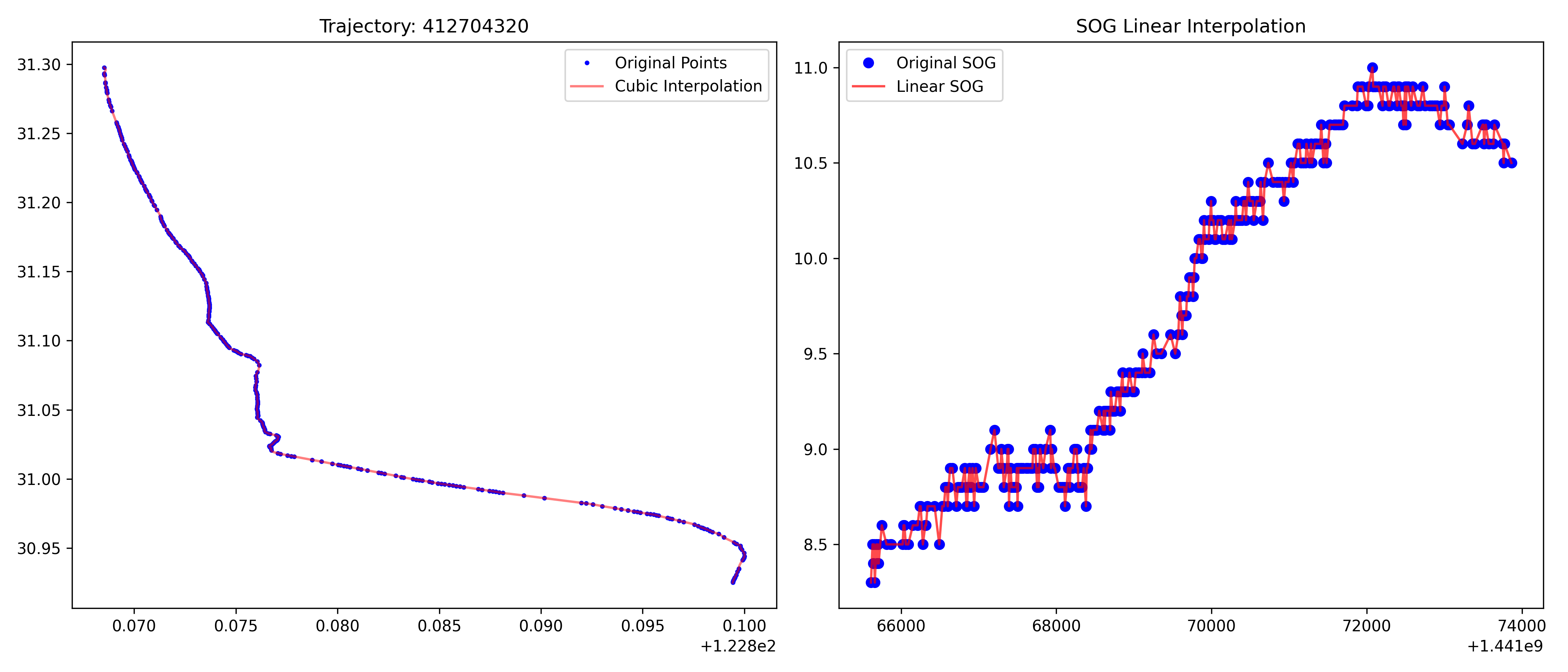

Supplement: S1 File — (ZIP) [file pone.0342781.s001.zip › data/interpolation/shipid_412704320_plot.png]

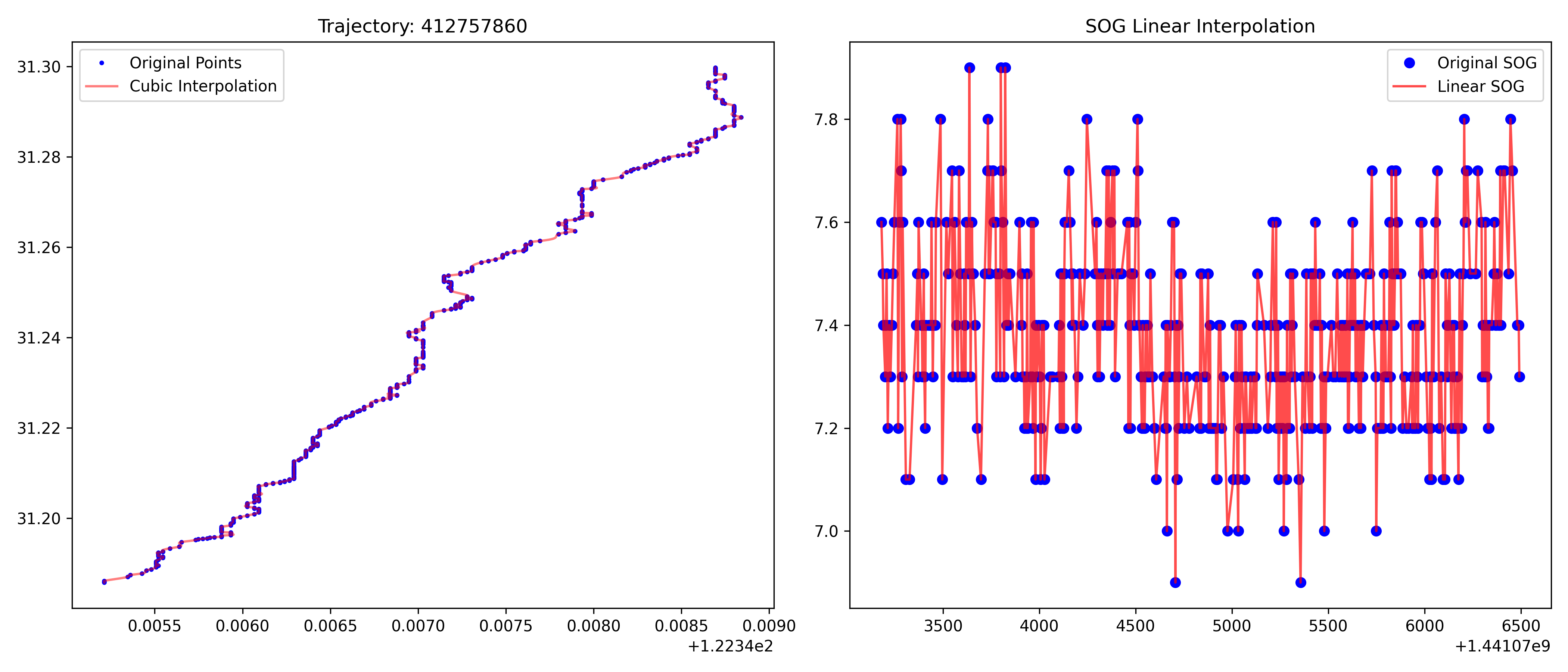

Supplement: S1 File — (ZIP) [file pone.0342781.s001.zip › data/interpolation/shipid_412757860_plot.png]

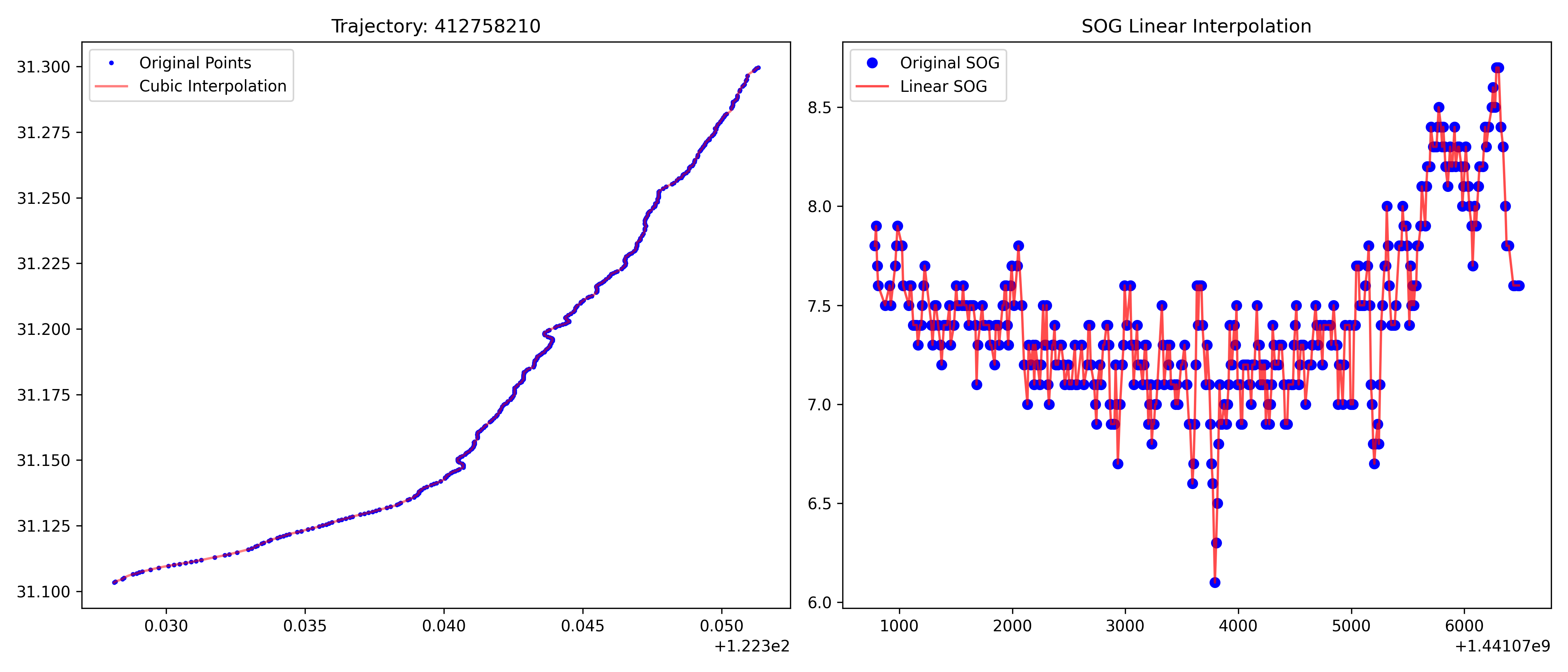

Supplement: S1 File — (ZIP) [file pone.0342781.s001.zip › data/interpolation/shipid_412758210_plot.png]

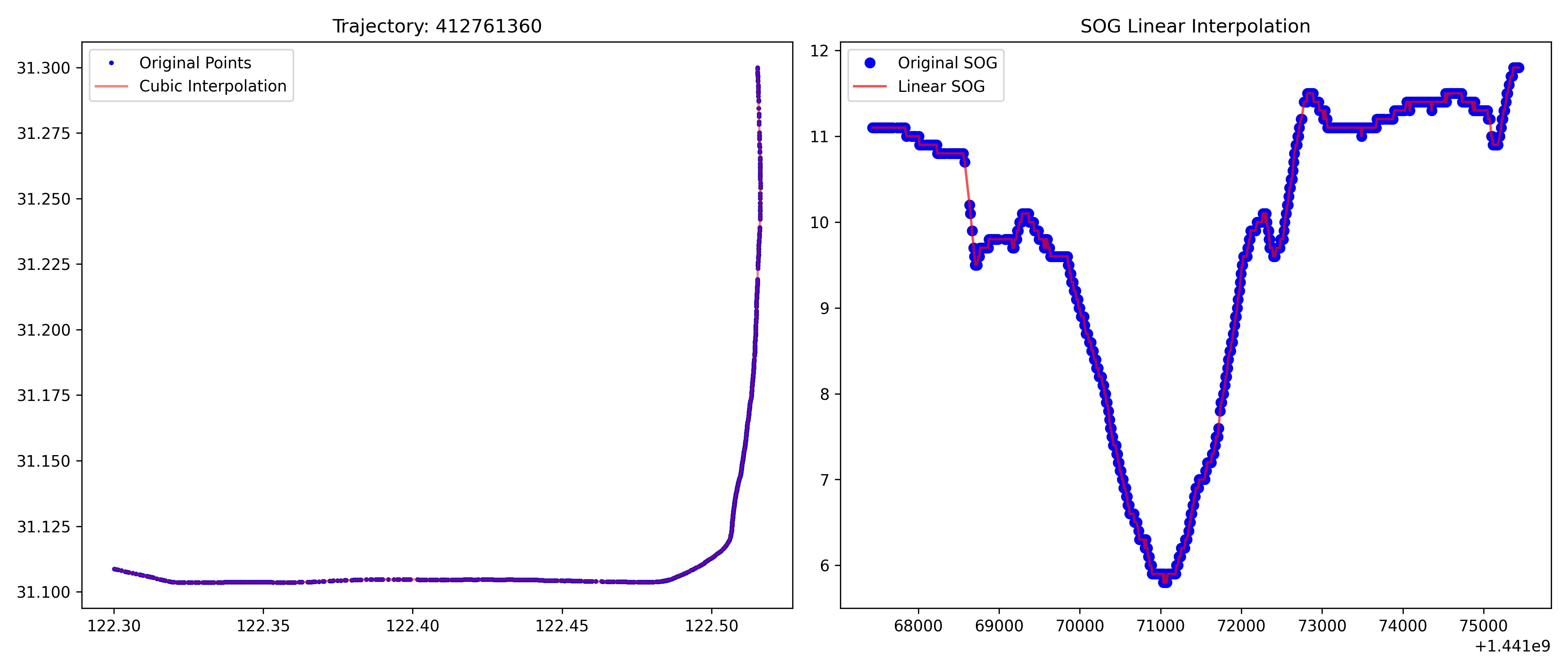

Supplement: S1 File — (ZIP) [file pone.0342781.s001.zip › data/interpolation/shipid_412761360_plot.png]

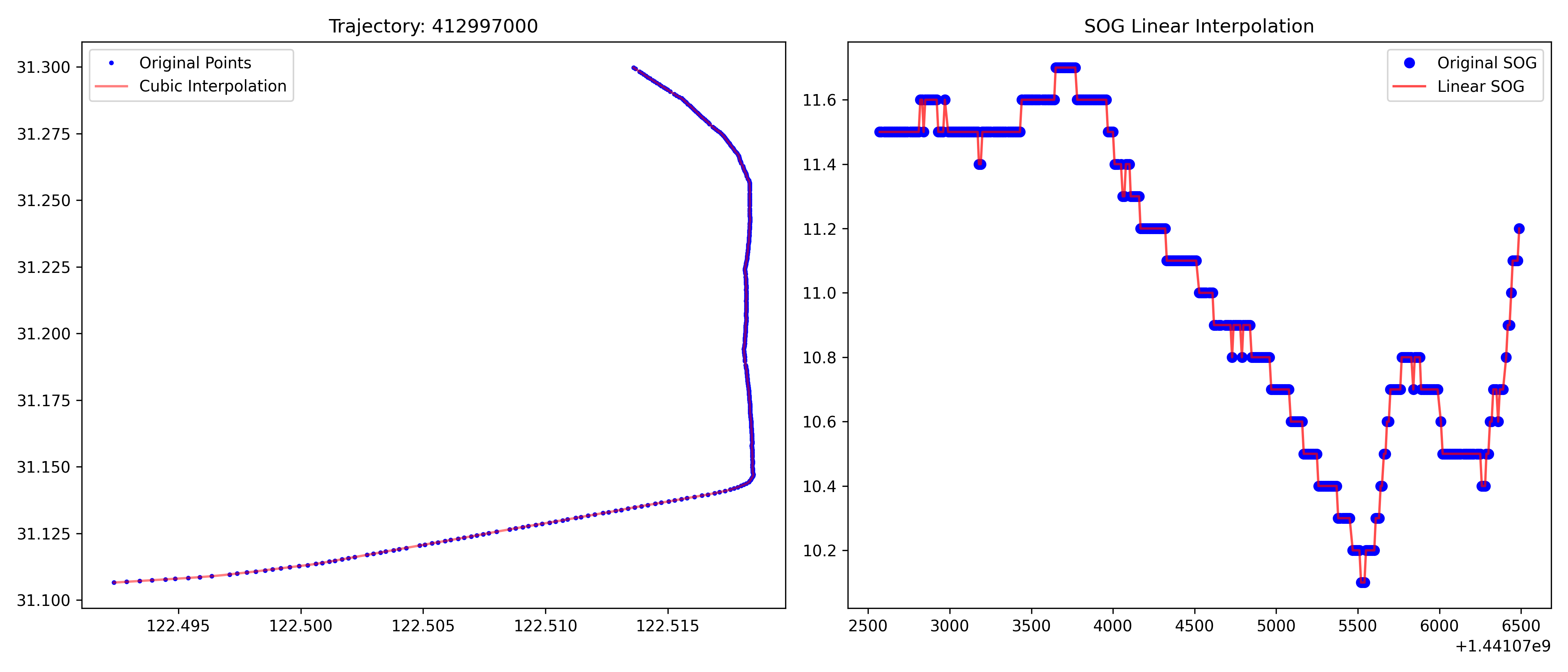

Supplement: S1 File — (ZIP) [file pone.0342781.s001.zip › data/interpolation/shipid_412997000_plot.png]

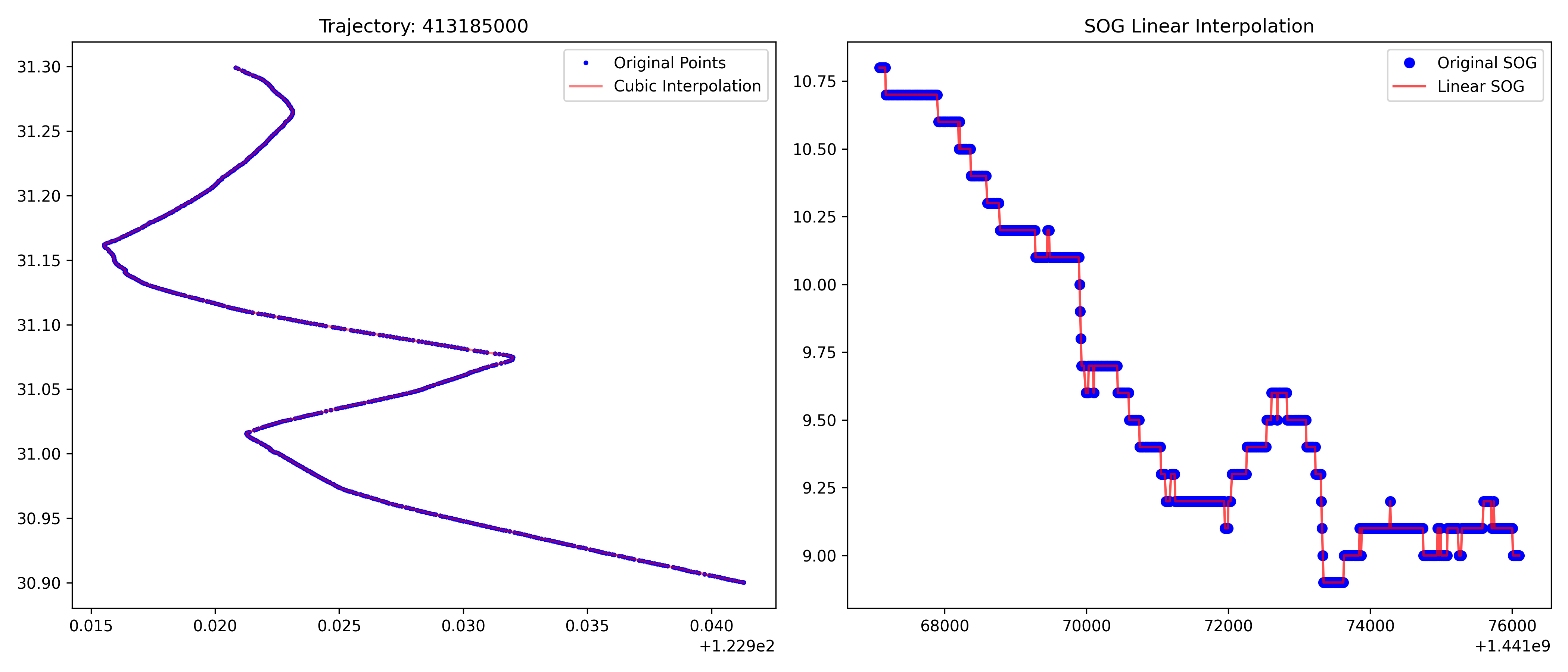

Supplement: S1 File — (ZIP) [file pone.0342781.s001.zip › data/interpolation/shipid_413185000_plot.png]

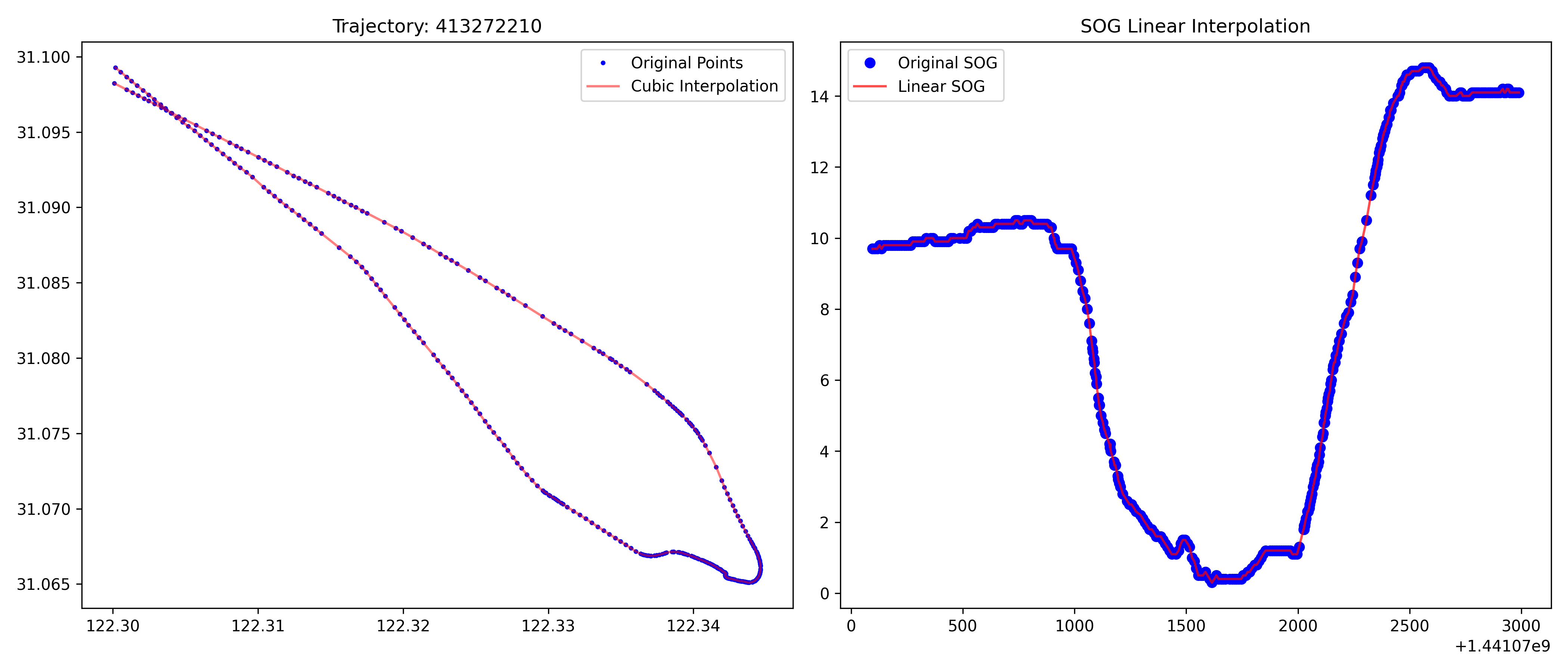

Supplement: S1 File — (ZIP) [file pone.0342781.s001.zip › data/interpolation/shipid_413272210_plot.png]

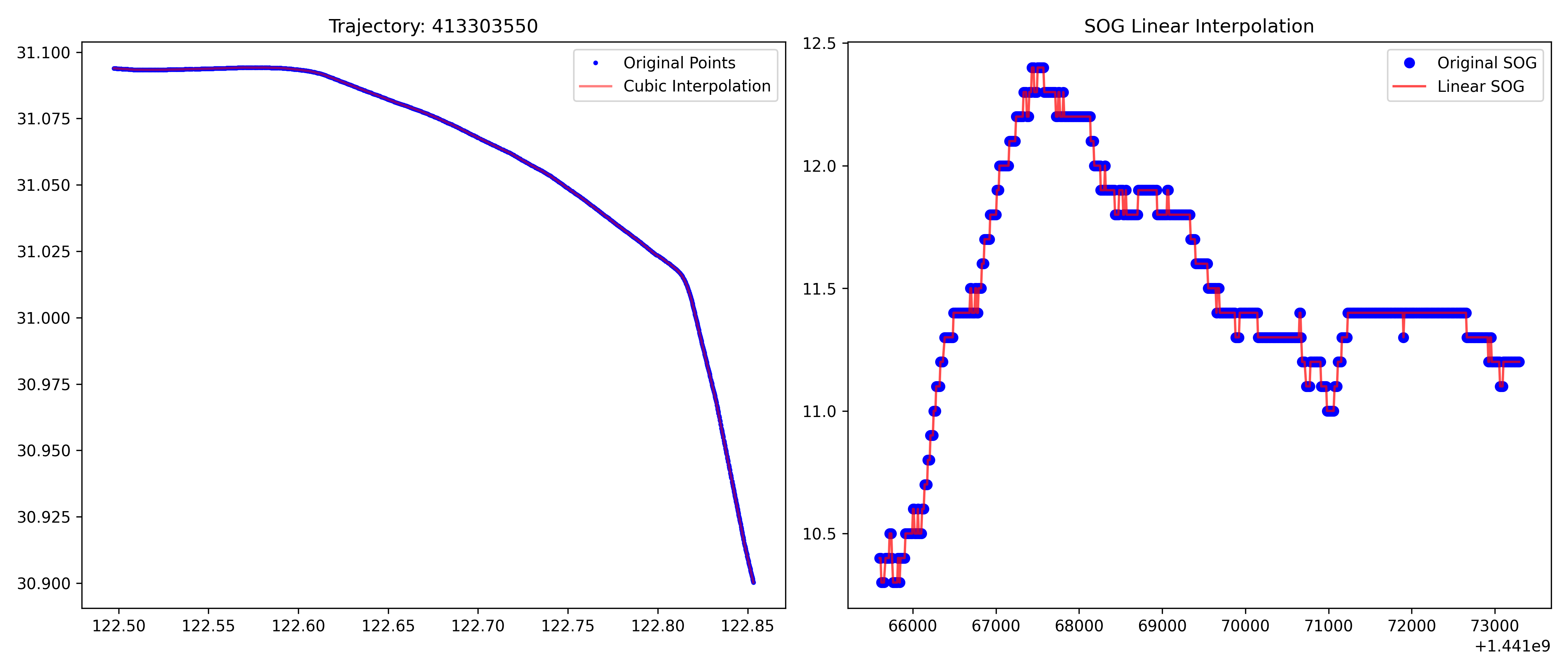

Supplement: S1 File — (ZIP) [file pone.0342781.s001.zip › data/interpolation/shipid_413303550_plot.png]

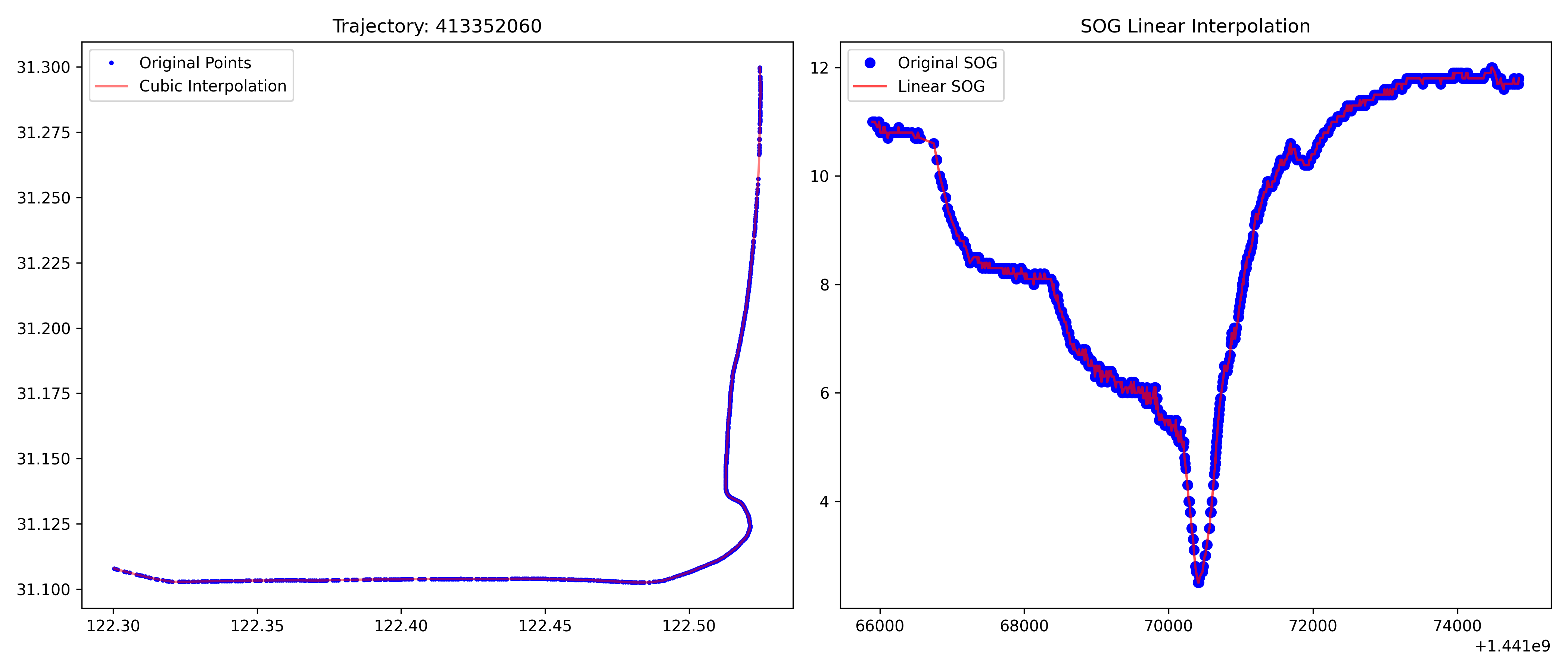

Supplement: S1 File — (ZIP) [file pone.0342781.s001.zip › data/interpolation/shipid_413352060_plot.png]

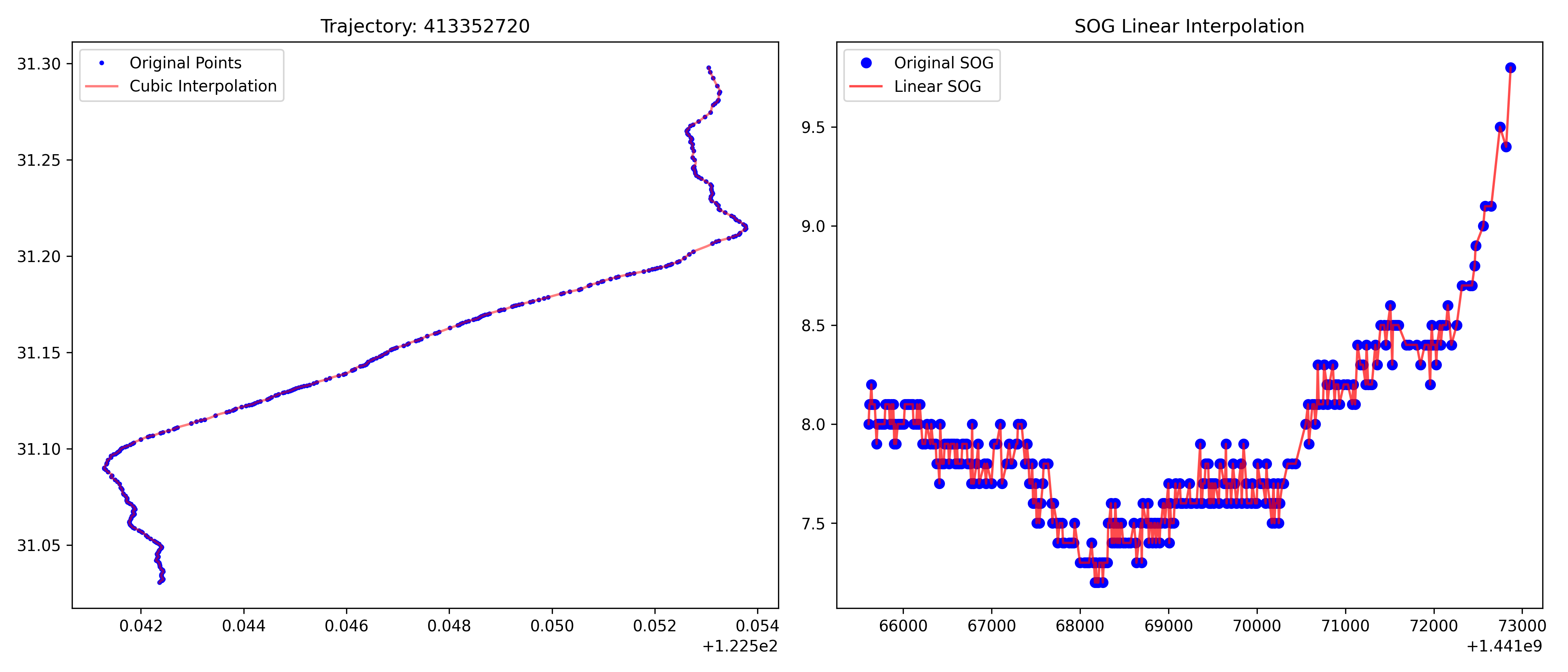

Supplement: S1 File — (ZIP) [file pone.0342781.s001.zip › data/interpolation/shipid_413352720_plot.png]

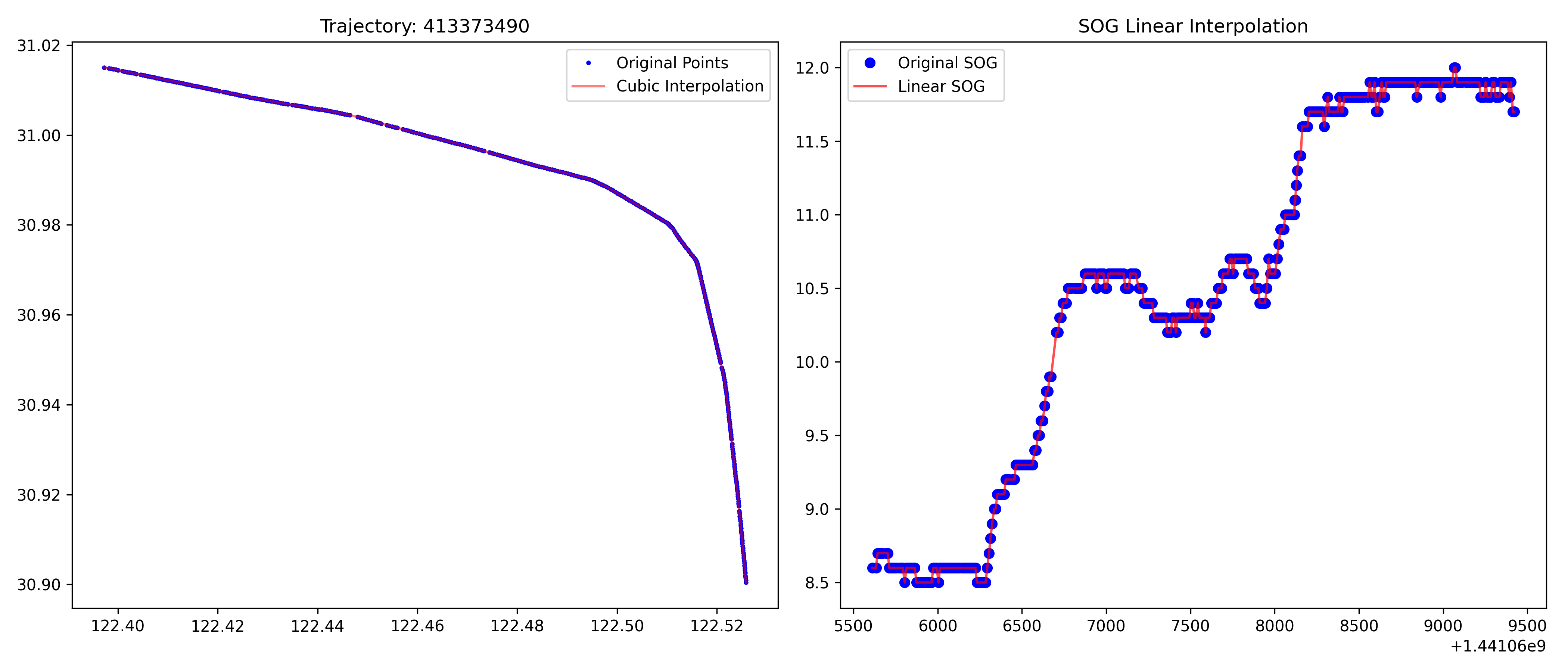

Supplement: S1 File — (ZIP) [file pone.0342781.s001.zip › data/interpolation/shipid_413373490_plot.png]

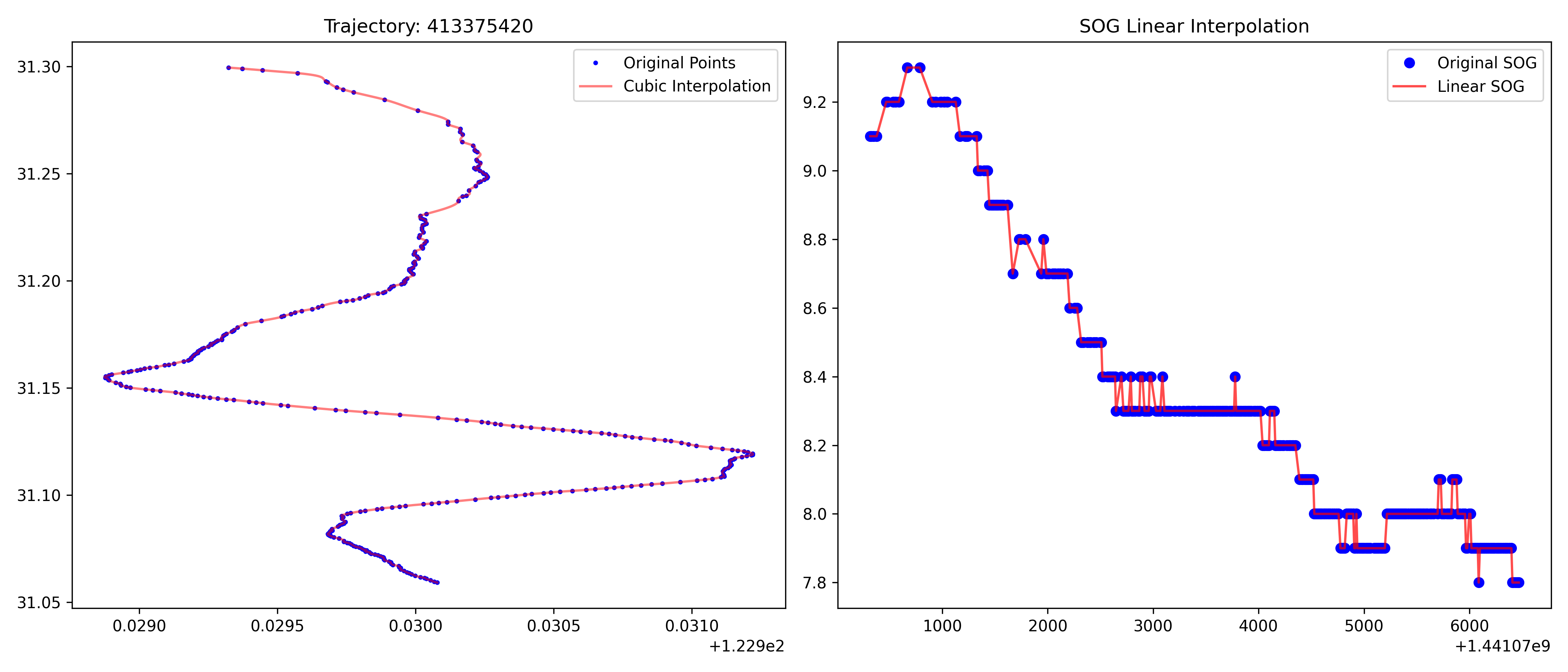

Supplement: S1 File — (ZIP) [file pone.0342781.s001.zip › data/interpolation/shipid_413375420_plot.png]

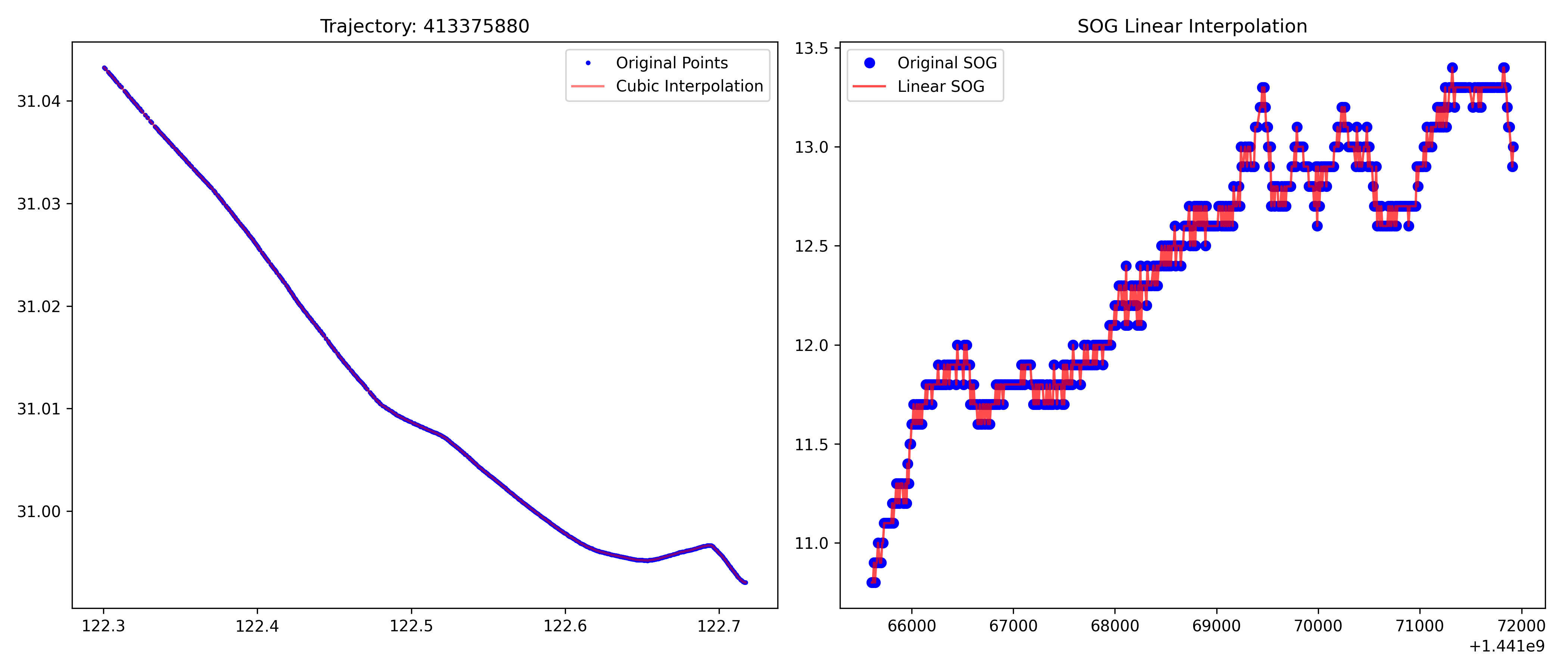

Supplement: S1 File — (ZIP) [file pone.0342781.s001.zip › data/interpolation/shipid_413375880_plot.png]

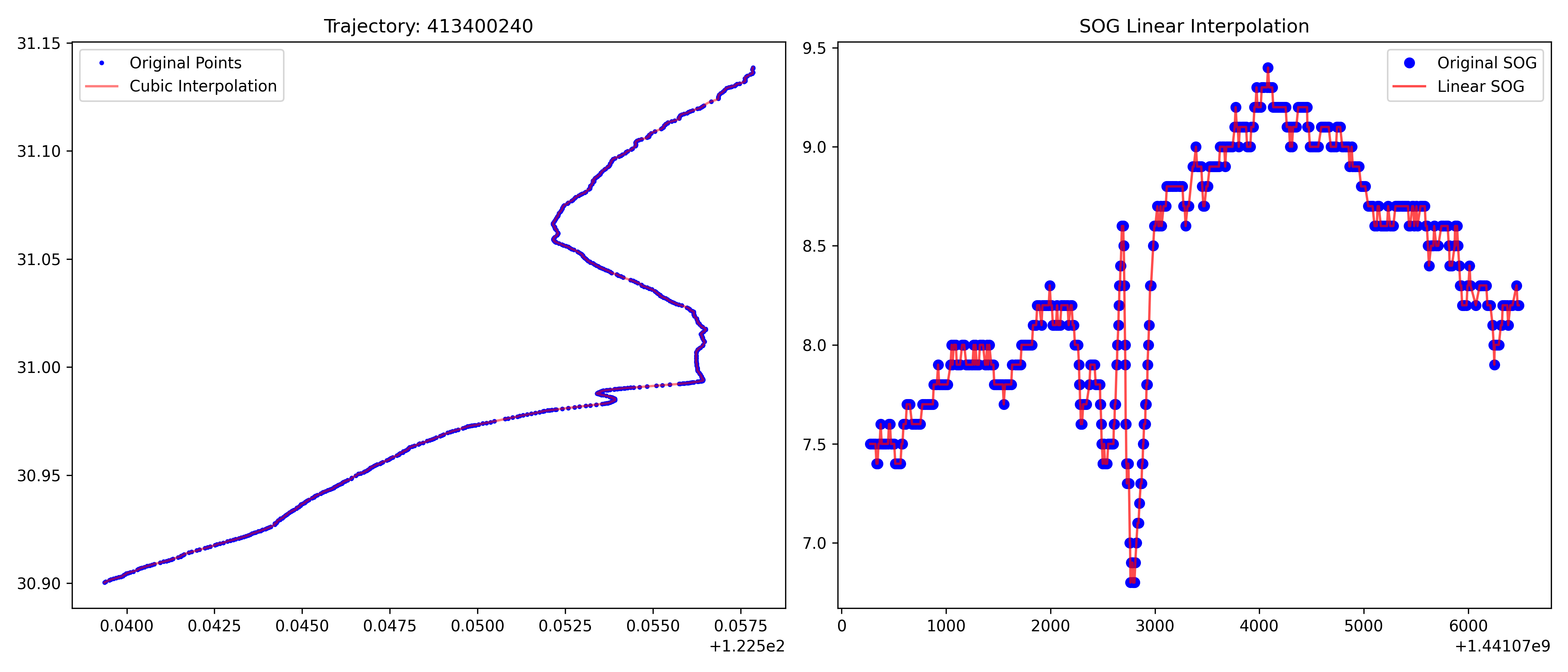

Supplement: S1 File — (ZIP) [file pone.0342781.s001.zip › data/interpolation/shipid_413400240_plot.png]

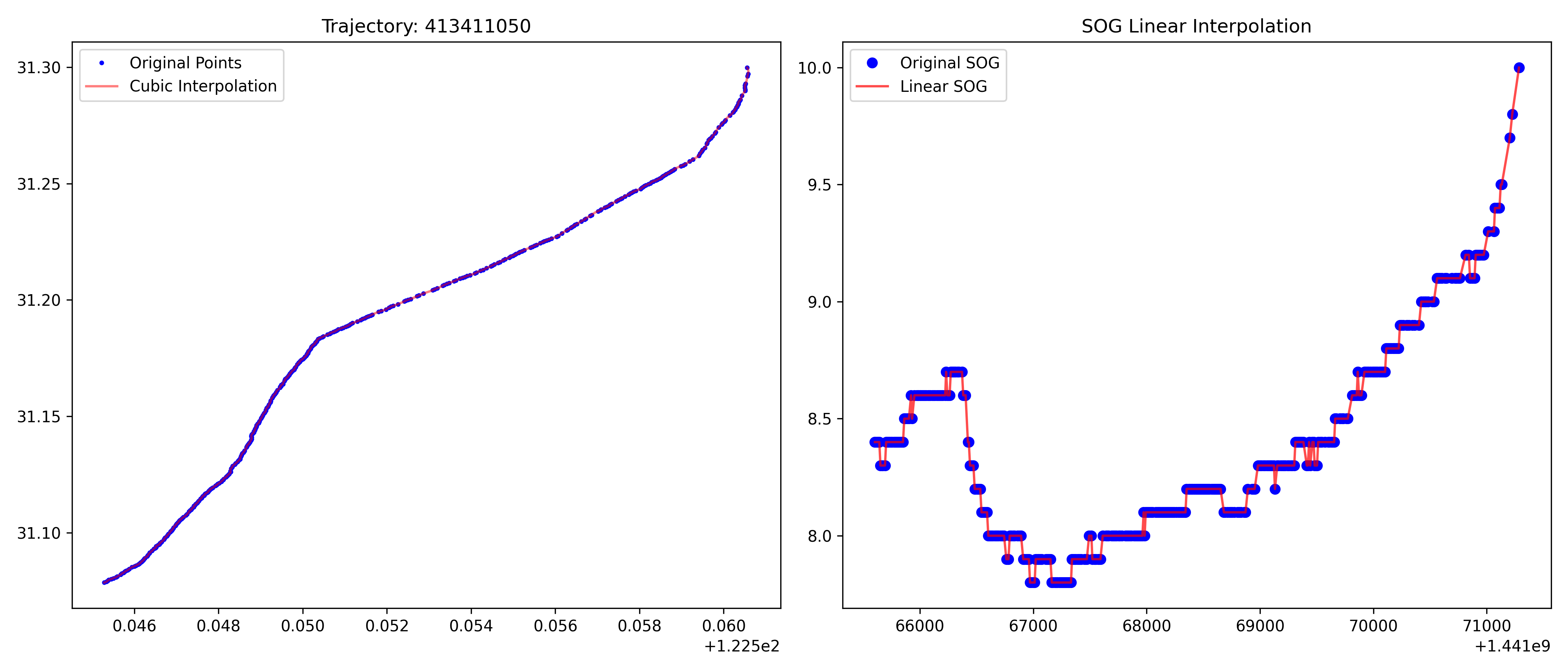

Supplement: S1 File — (ZIP) [file pone.0342781.s001.zip › data/interpolation/shipid_413411050_plot.png]

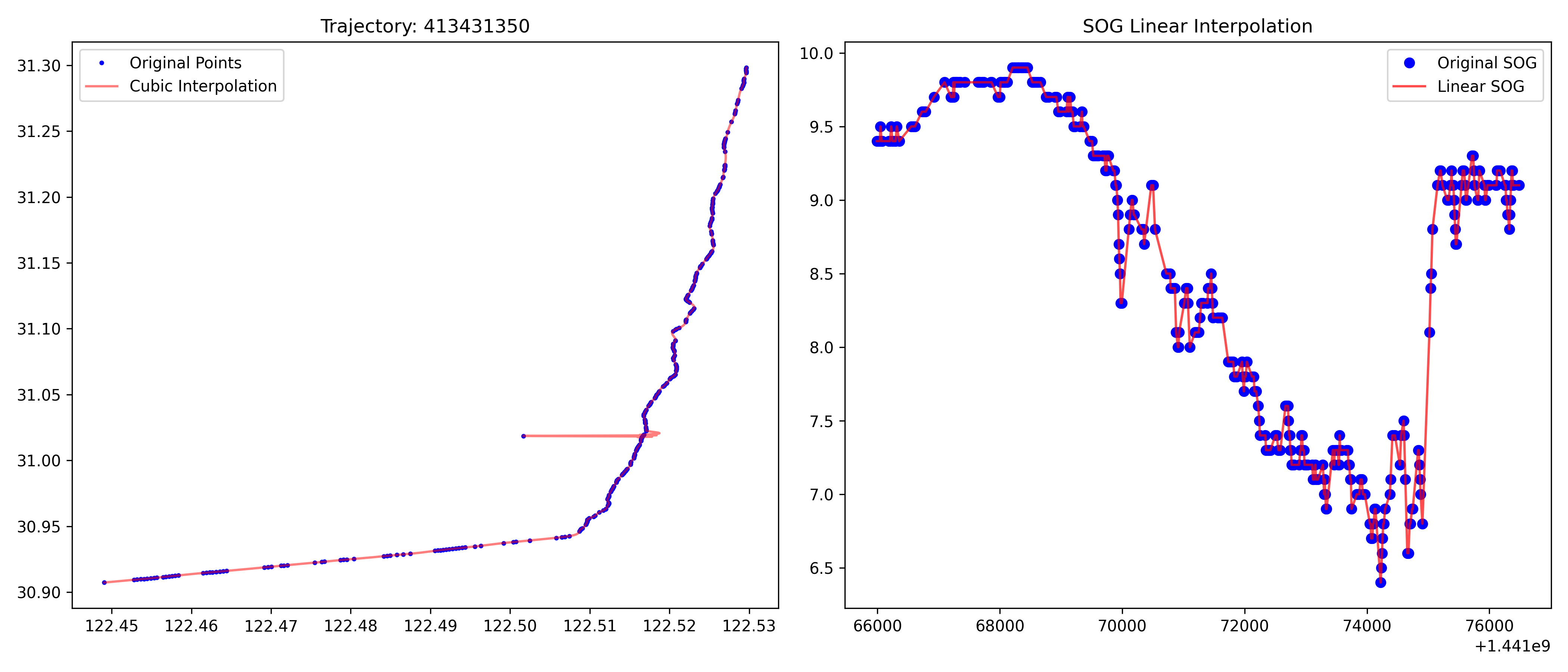

Supplement: S1 File — (ZIP) [file pone.0342781.s001.zip › data/interpolation/shipid_413431350_plot.png]

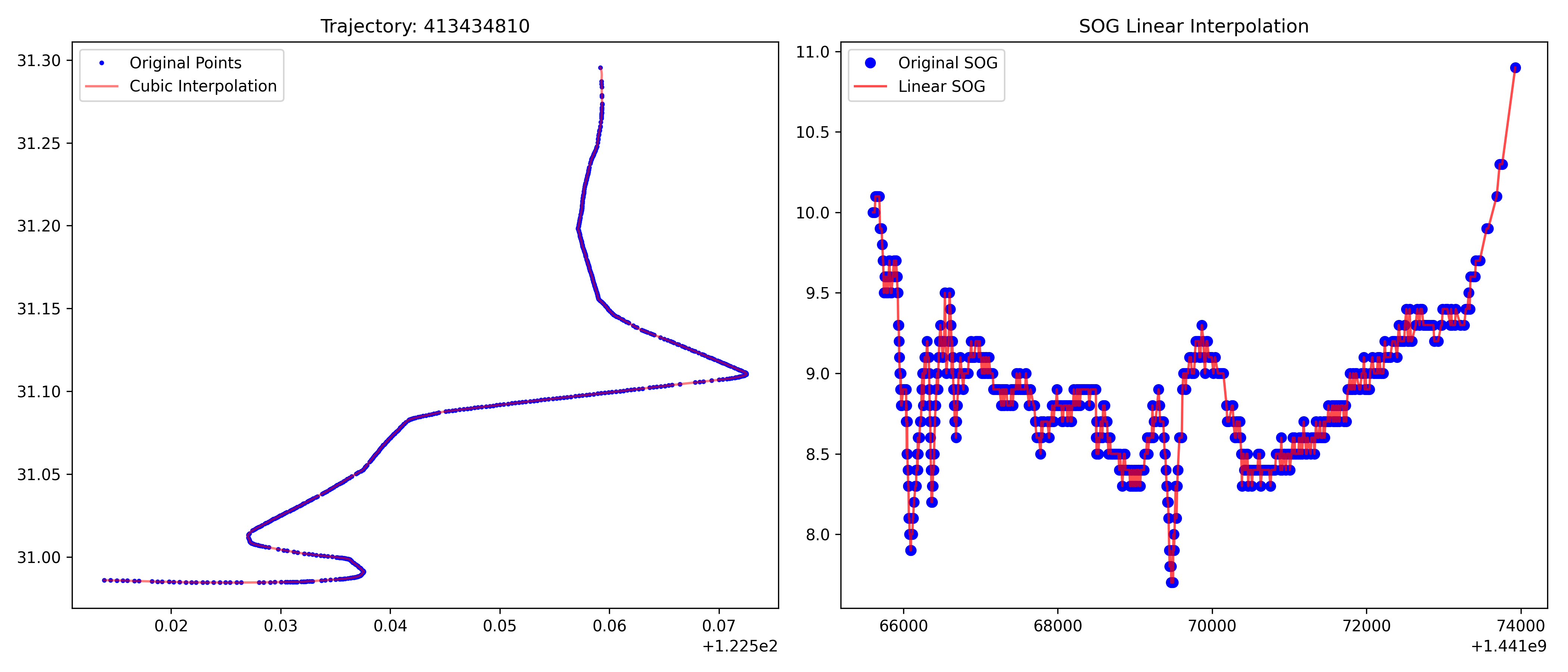

Supplement: S1 File — (ZIP) [file pone.0342781.s001.zip › data/interpolation/shipid_413434810_plot.png]

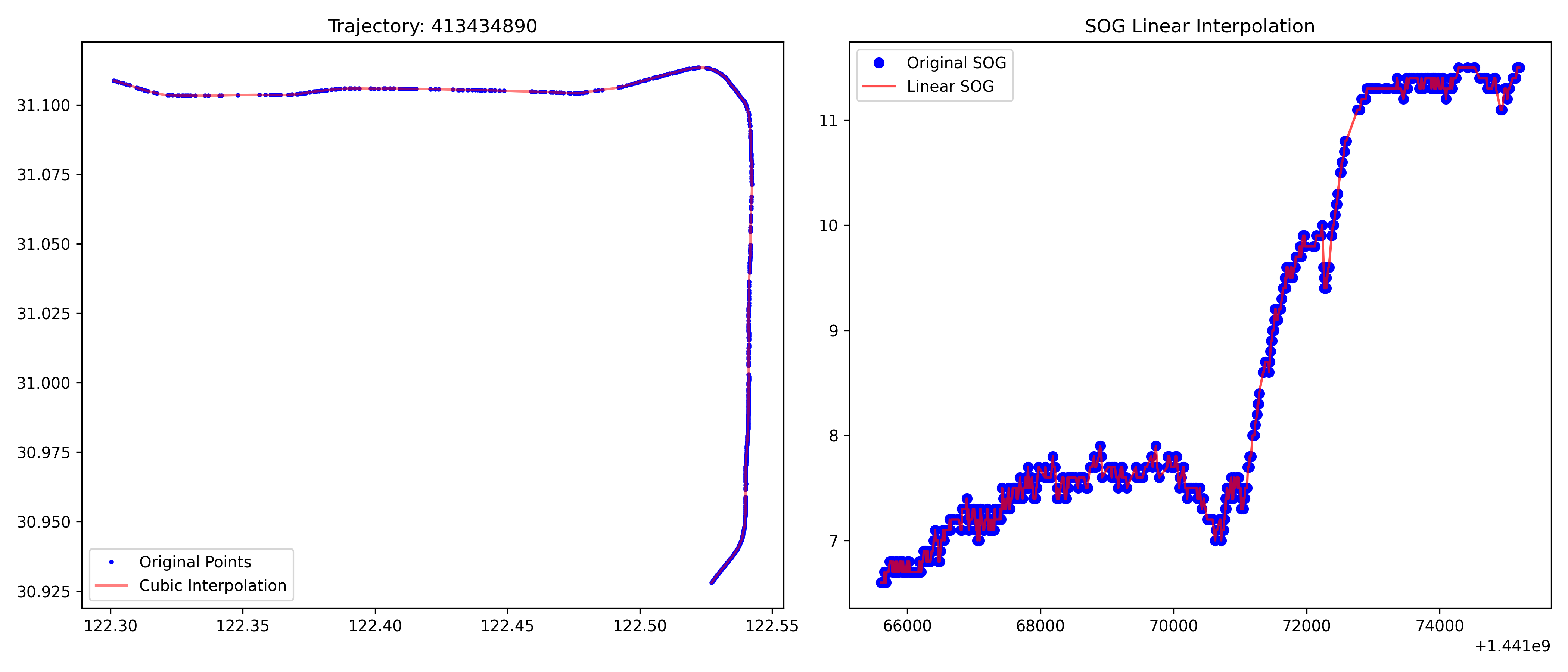

Supplement: S1 File — (ZIP) [file pone.0342781.s001.zip › data/interpolation/shipid_413434890_plot.png]

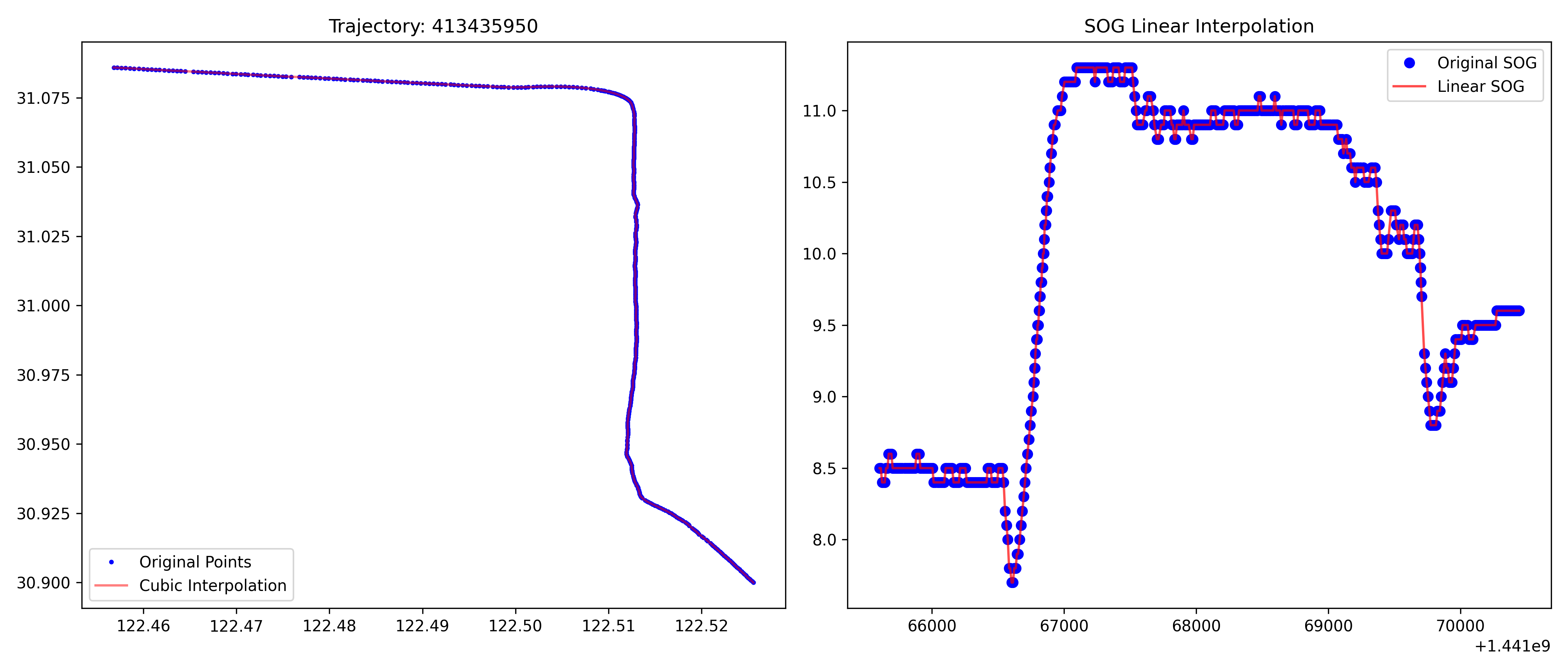

Supplement: S1 File — (ZIP) [file pone.0342781.s001.zip › data/interpolation/shipid_413435950_plot.png]

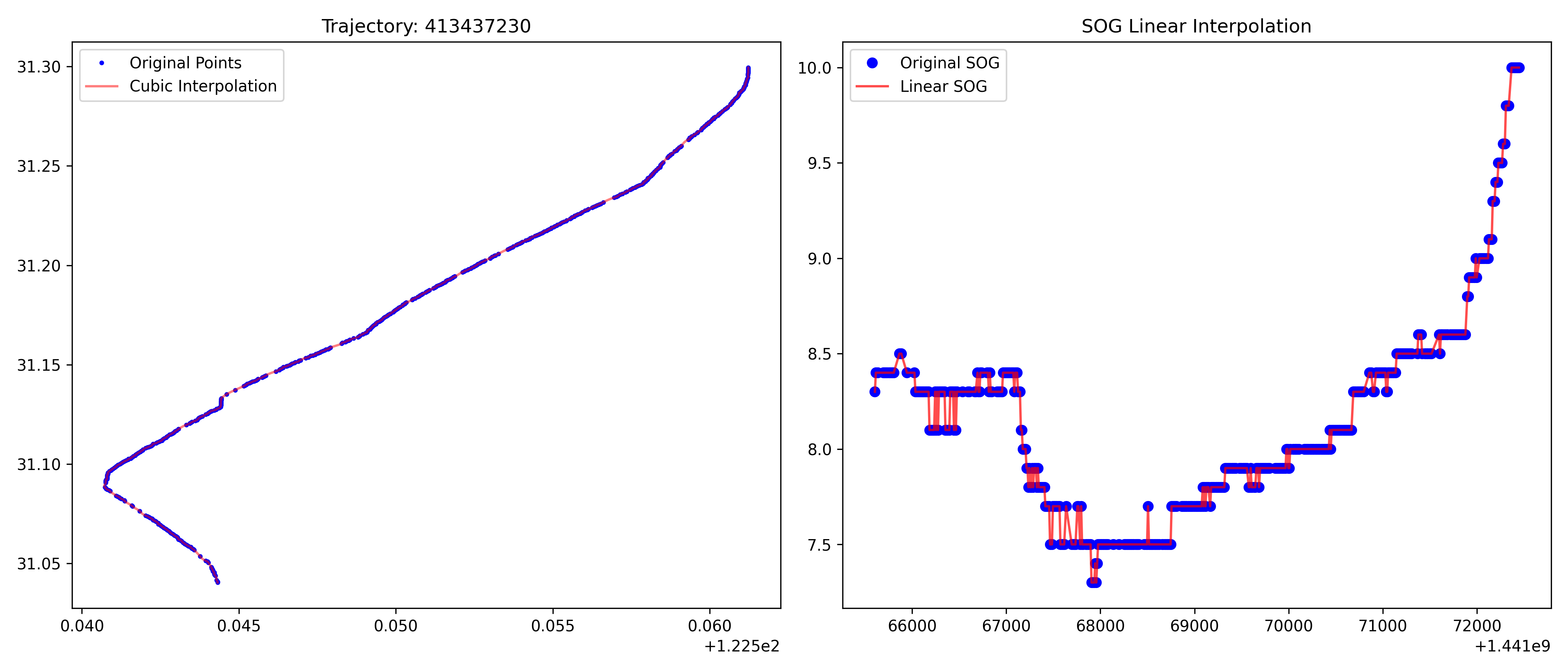

Supplement: S1 File — (ZIP) [file pone.0342781.s001.zip › data/interpolation/shipid_413437230_plot.png]

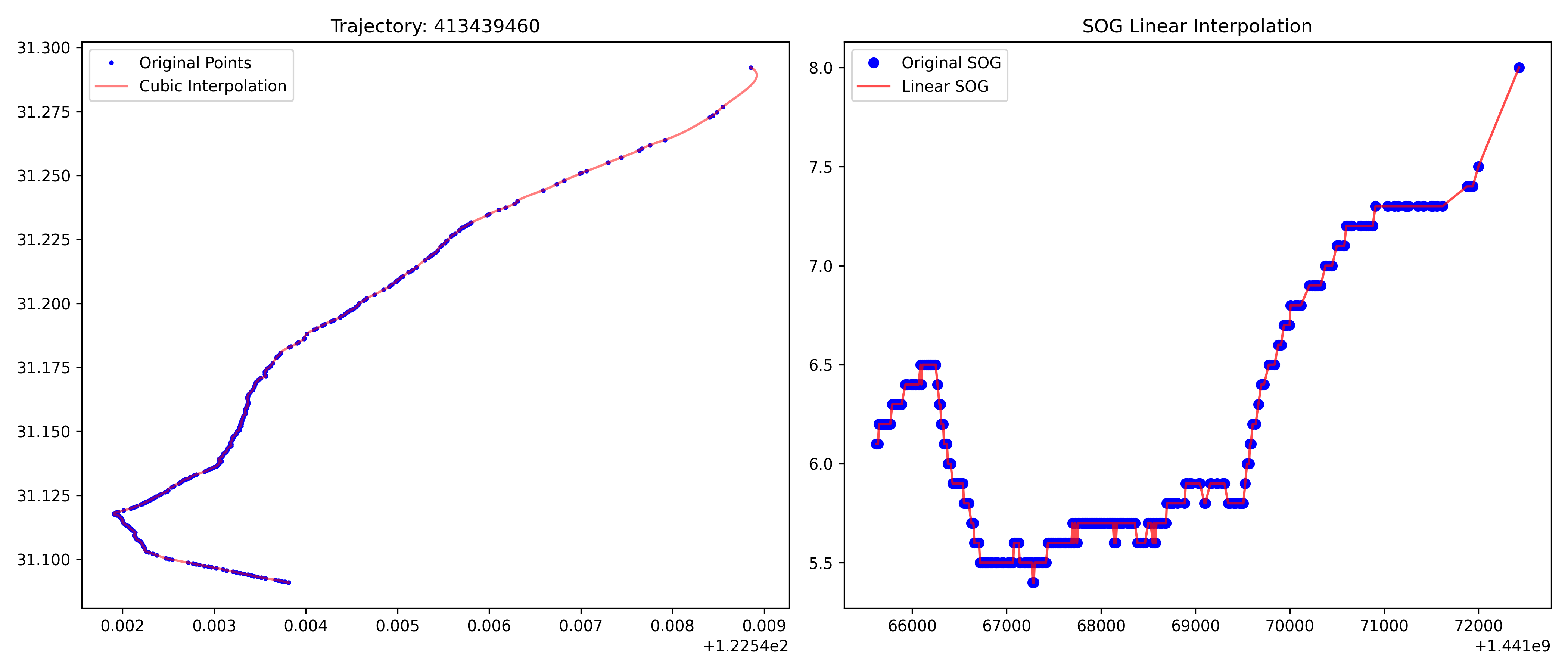

Supplement: S1 File — (ZIP) [file pone.0342781.s001.zip › data/interpolation/shipid_413439460_plot.png]

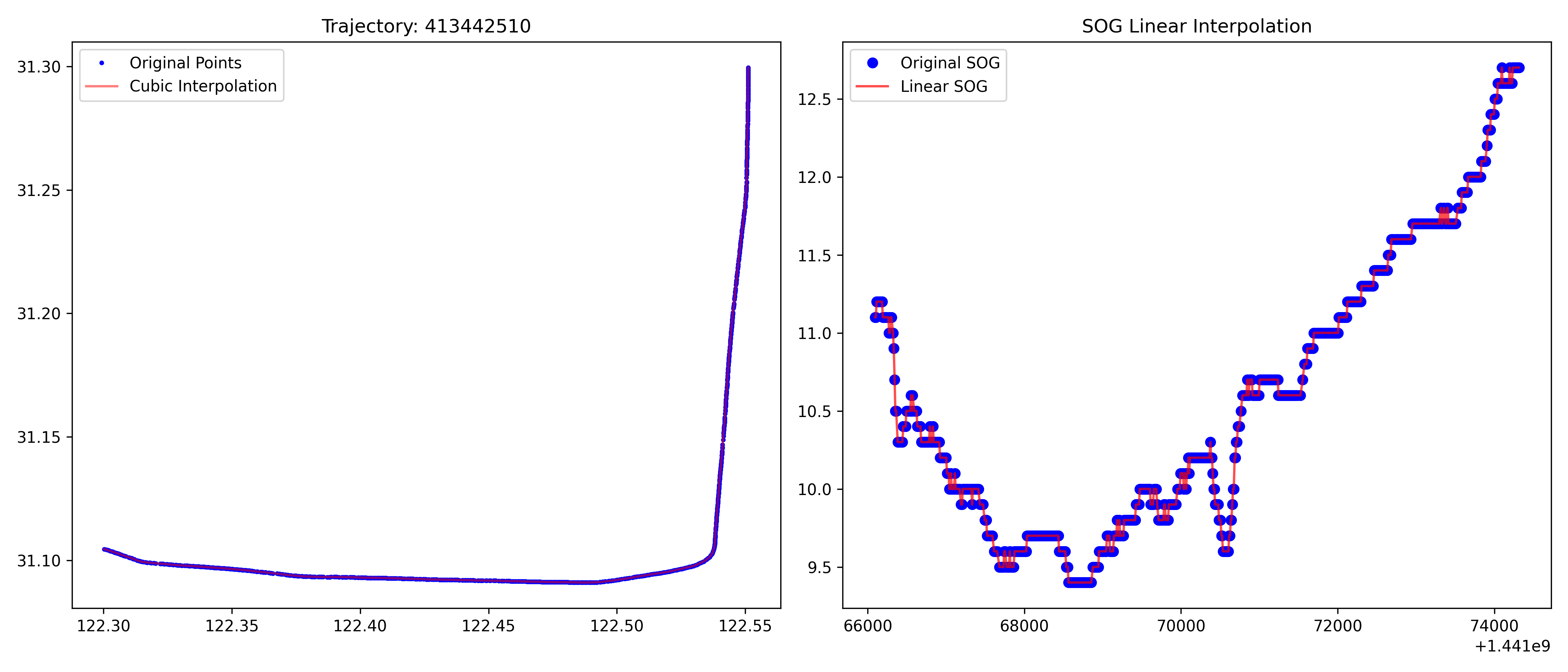

Supplement: S1 File — (ZIP) [file pone.0342781.s001.zip › data/interpolation/shipid_413442510_plot.png]

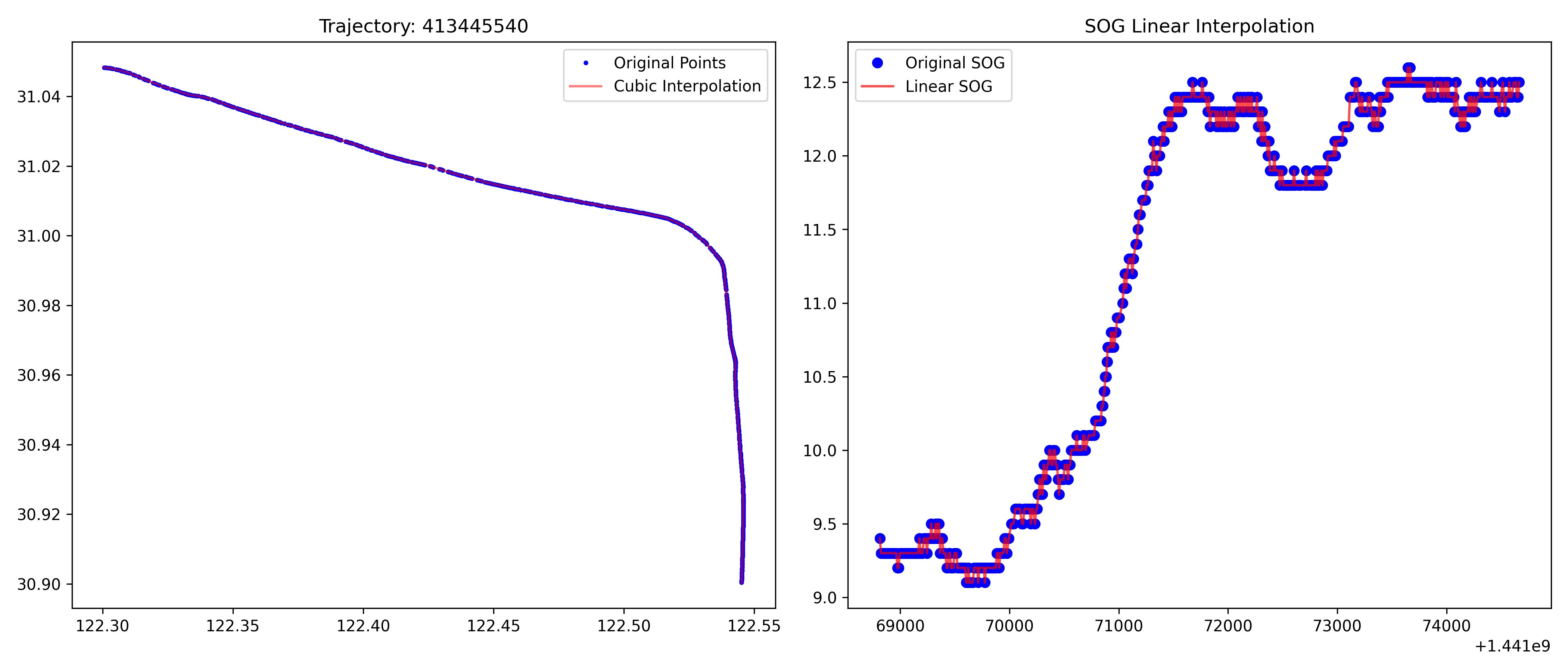

Supplement: S1 File — (ZIP) [file pone.0342781.s001.zip › data/interpolation/shipid_413445540_plot.png]

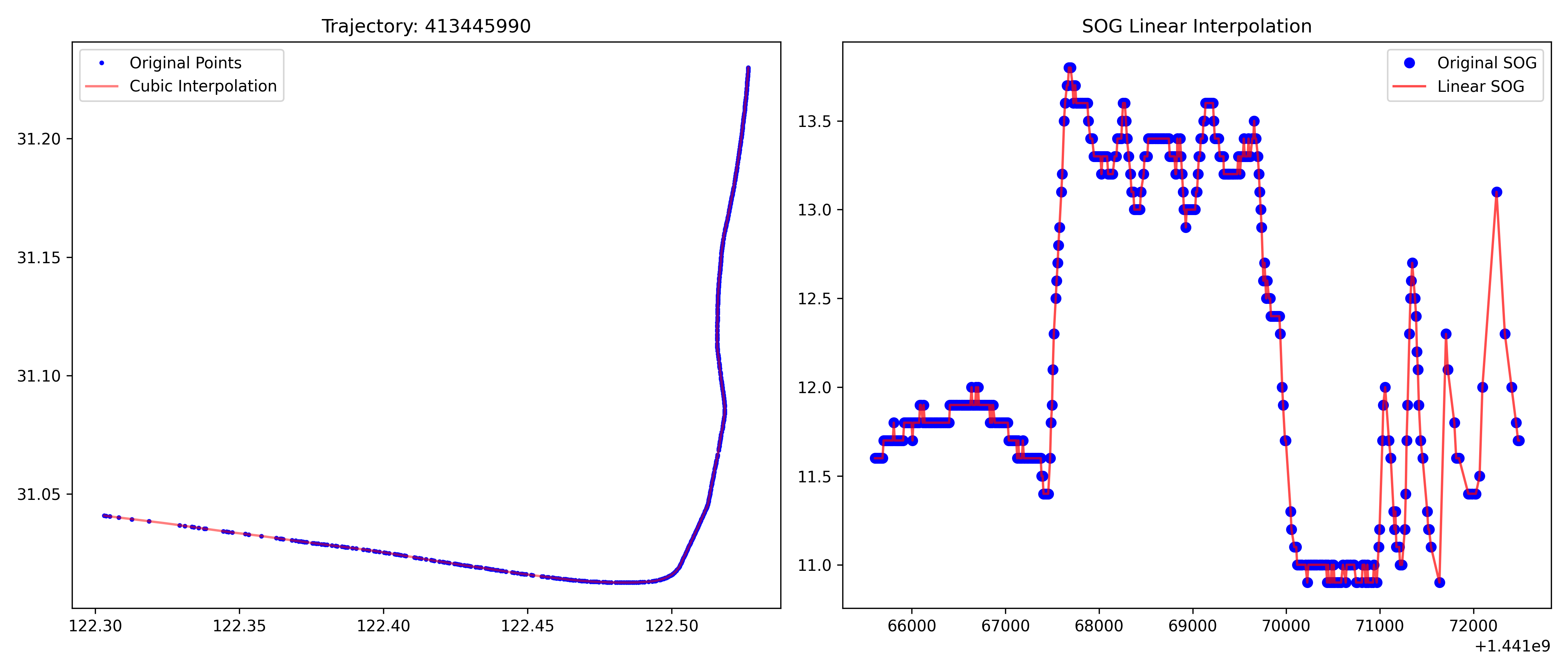

Supplement: S1 File — (ZIP) [file pone.0342781.s001.zip › data/interpolation/shipid_413445990_plot.png]

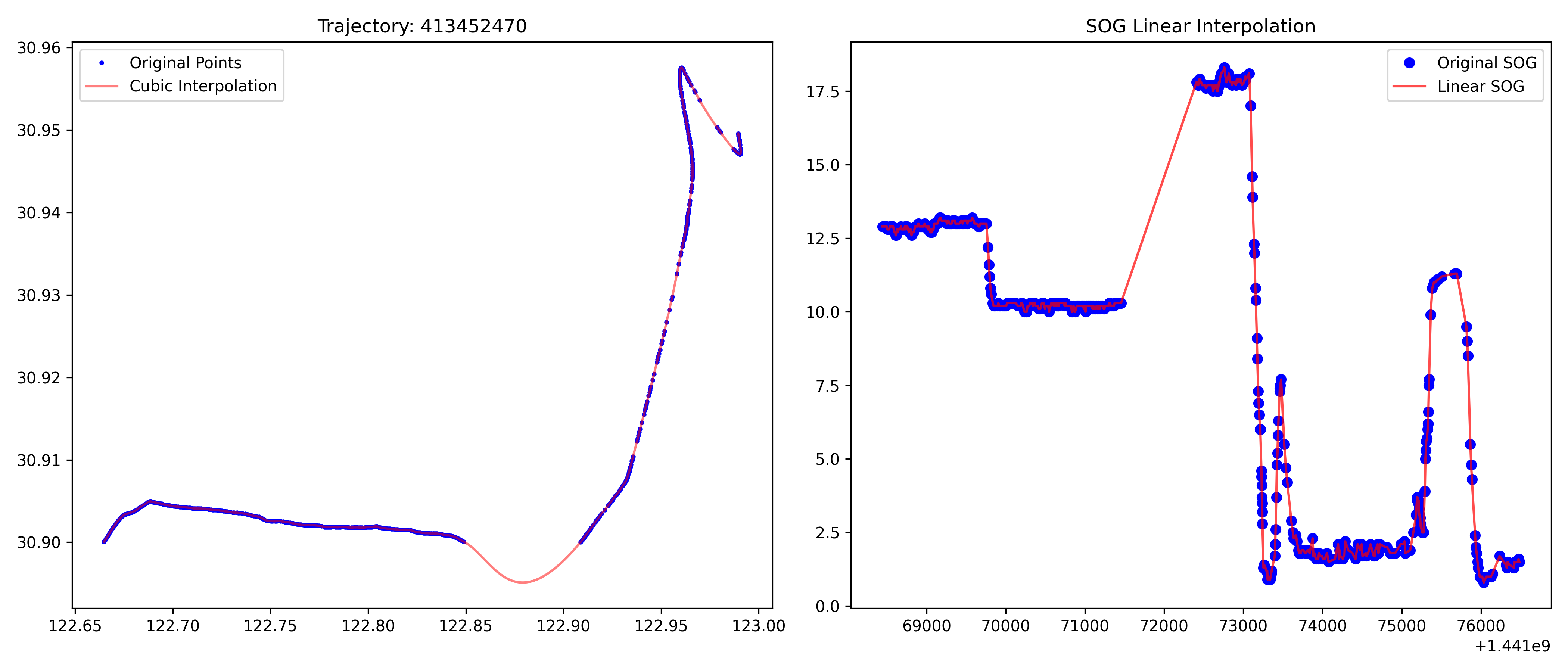

Supplement: S1 File — (ZIP) [file pone.0342781.s001.zip › data/interpolation/shipid_413452470_plot.png]

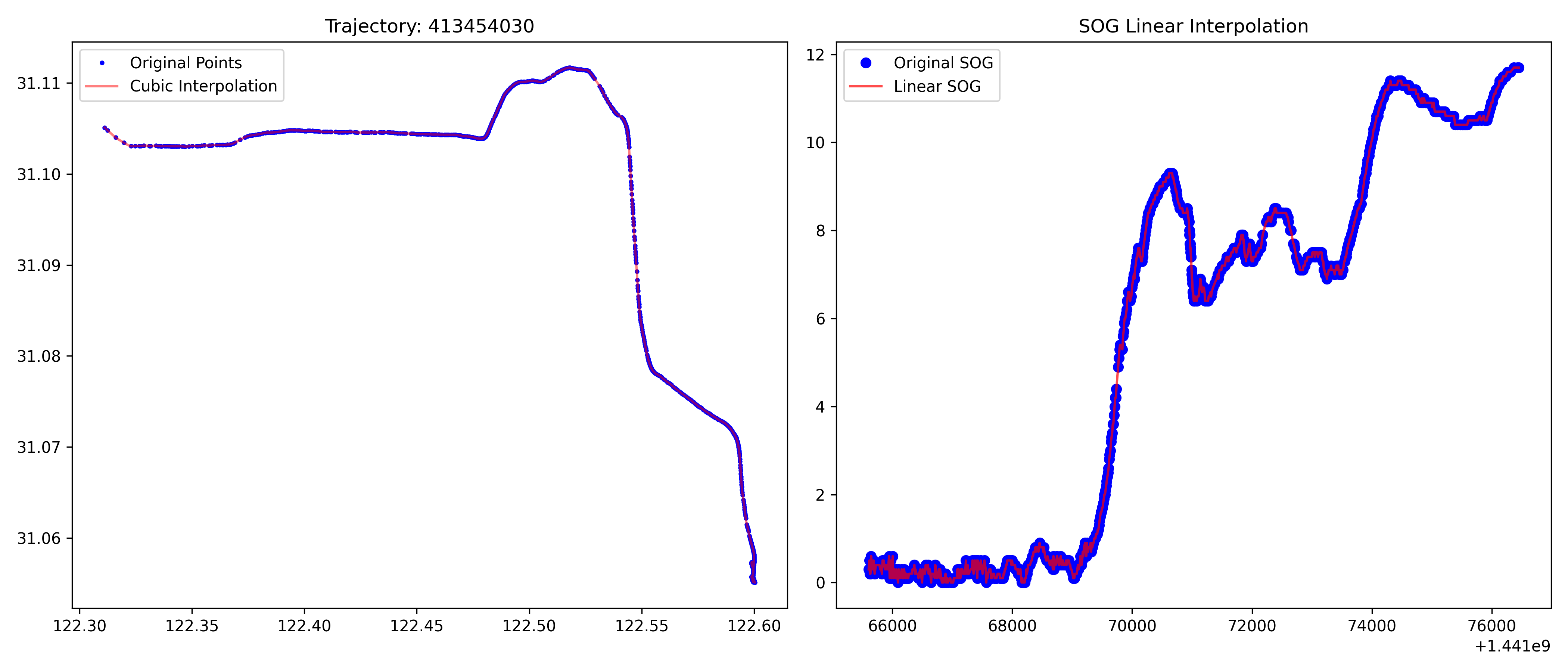

Supplement: S1 File — (ZIP) [file pone.0342781.s001.zip › data/interpolation/shipid_413454030_plot.png]

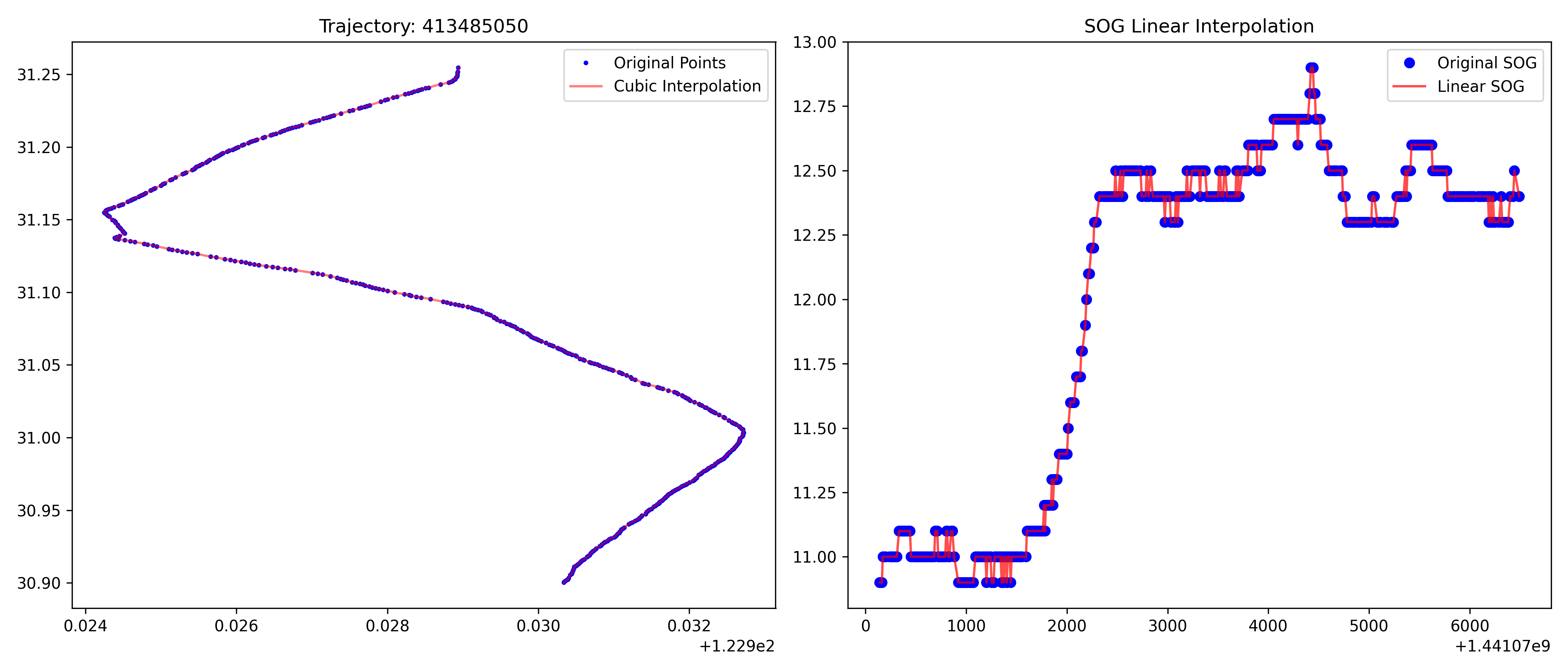

Supplement: S1 File — (ZIP) [file pone.0342781.s001.zip › data/interpolation/shipid_413485050_plot.png]

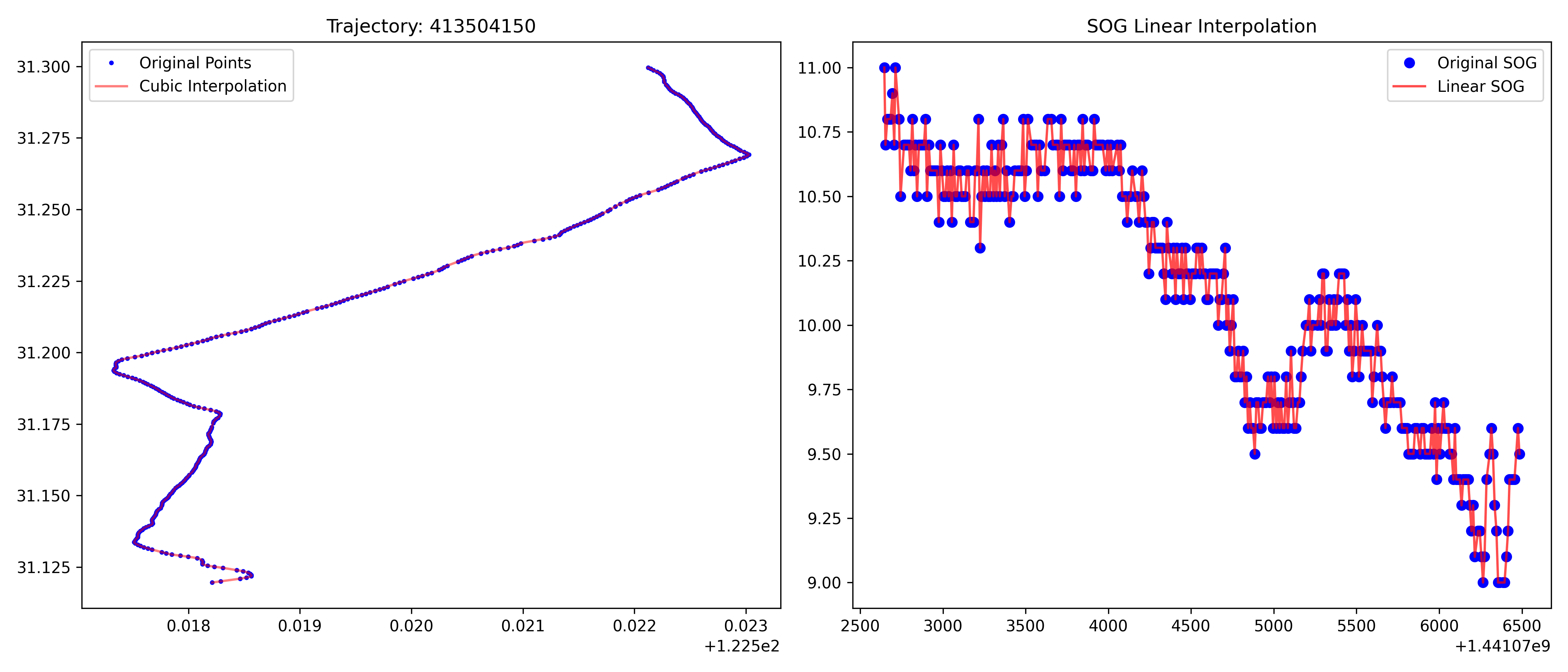

Supplement: S1 File — (ZIP) [file pone.0342781.s001.zip › data/interpolation/shipid_413504150_plot.png]

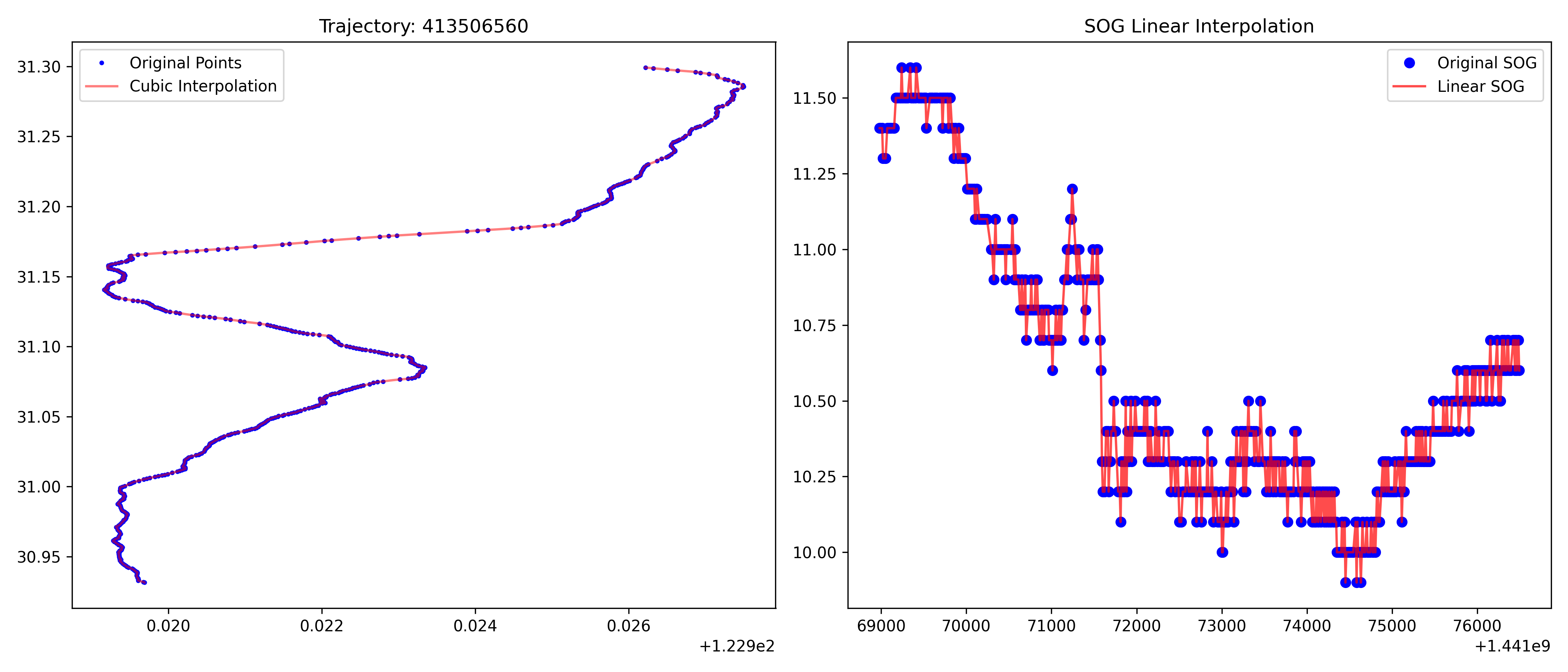

Supplement: S1 File — (ZIP) [file pone.0342781.s001.zip › data/interpolation/shipid_413506560_plot.png]

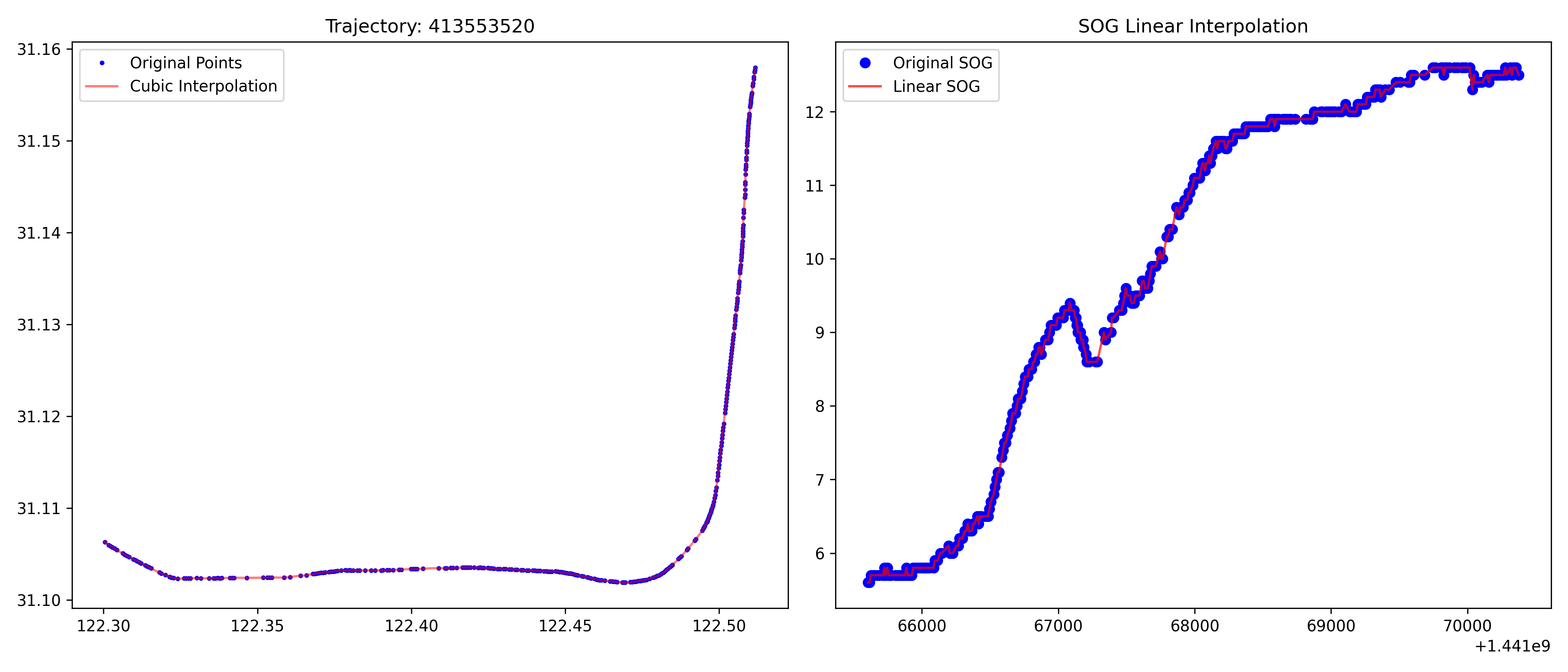

Supplement: S1 File — (ZIP) [file pone.0342781.s001.zip › data/interpolation/shipid_413553520_plot.png]

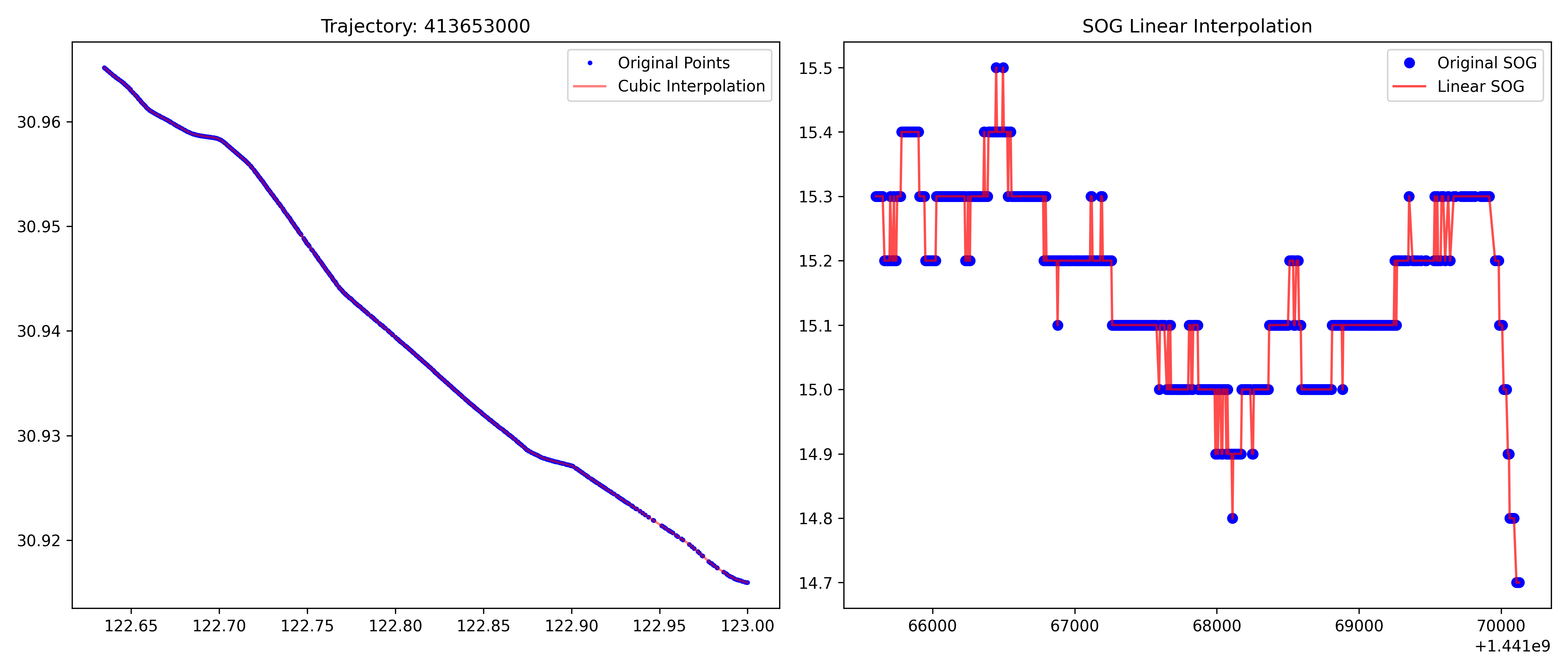

Supplement: S1 File — (ZIP) [file pone.0342781.s001.zip › data/interpolation/shipid_413653000_plot.png]

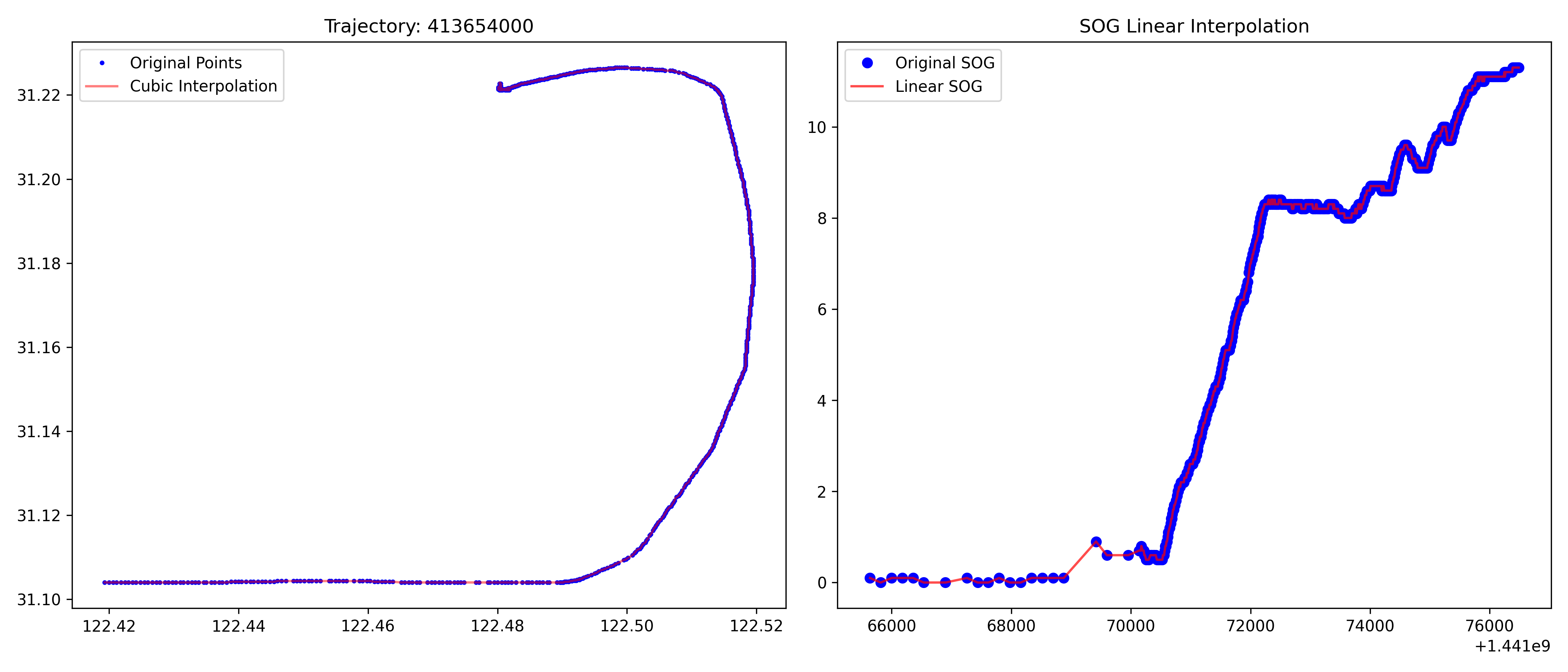

Supplement: S1 File — (ZIP) [file pone.0342781.s001.zip › data/interpolation/shipid_413654000_plot.png]

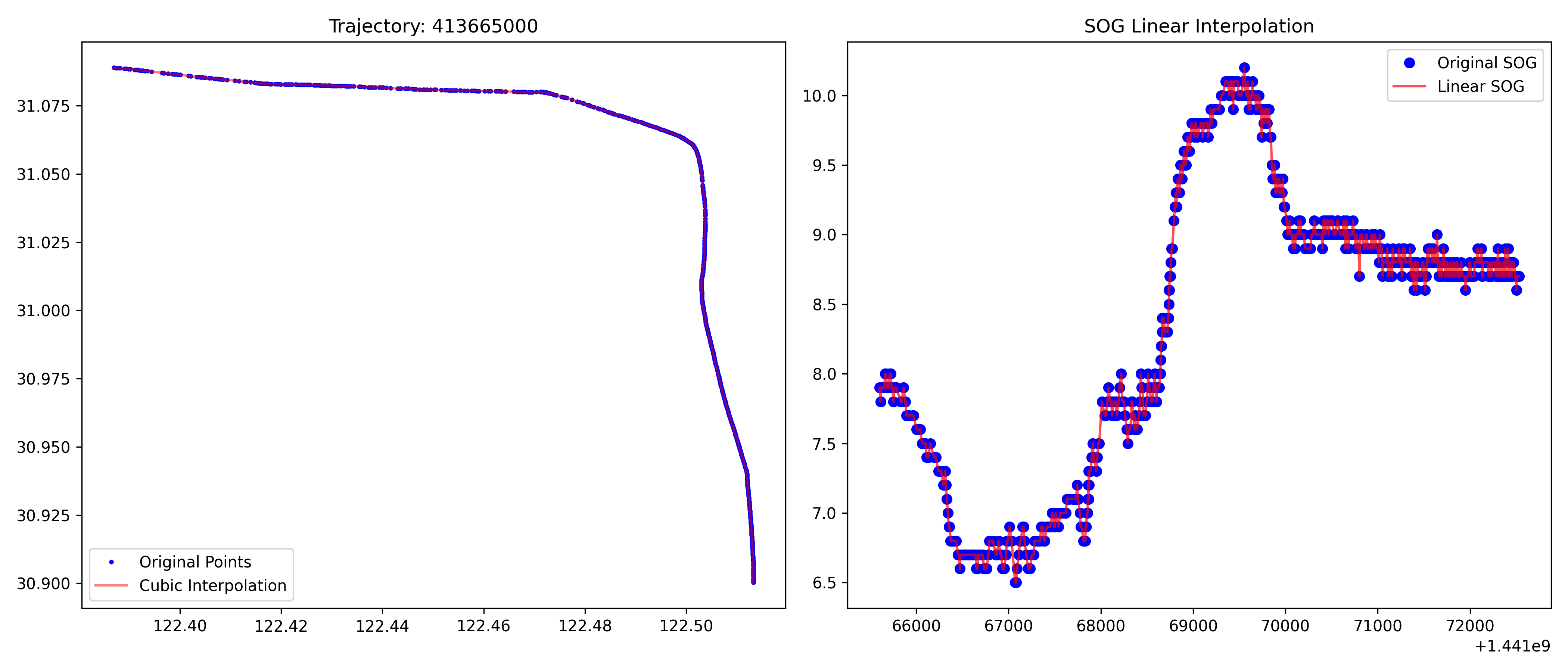

Supplement: S1 File — (ZIP) [file pone.0342781.s001.zip › data/interpolation/shipid_413665000_plot.png]

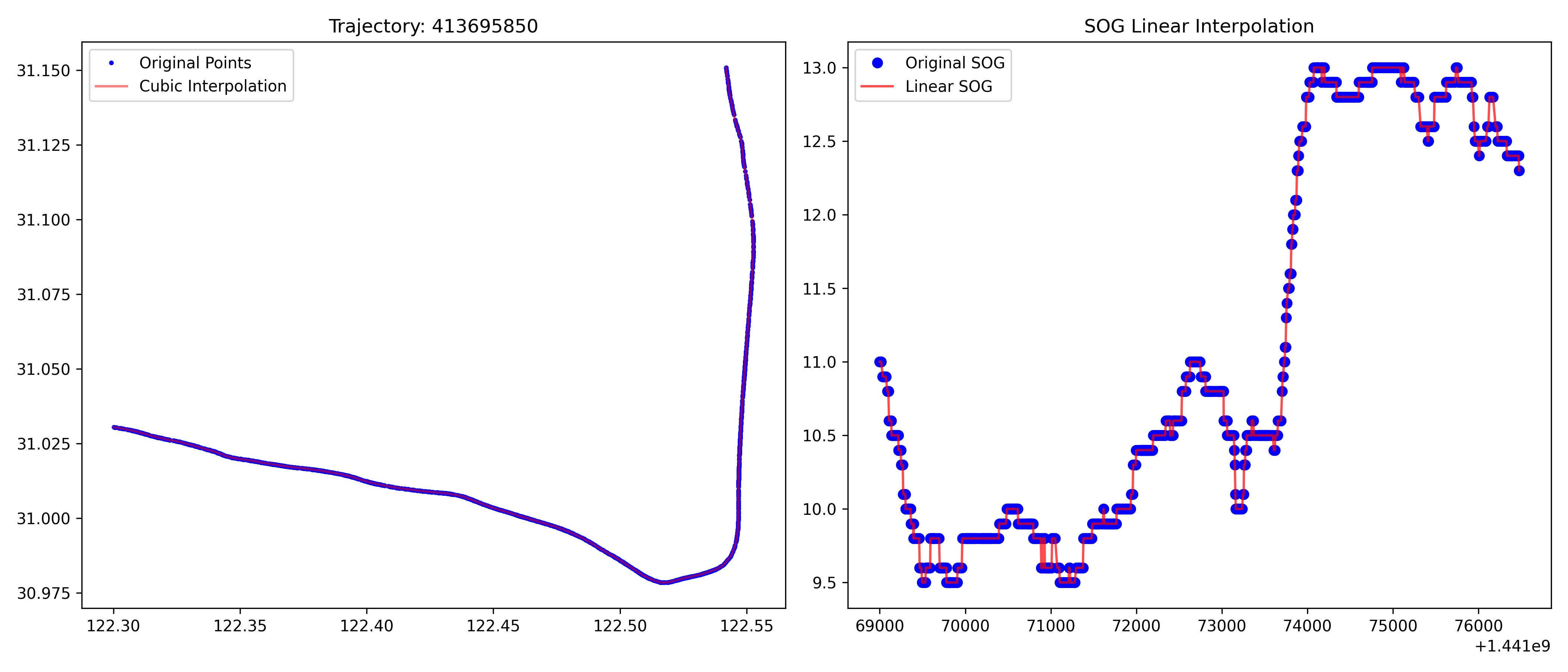

Supplement: S1 File — (ZIP) [file pone.0342781.s001.zip › data/interpolation/shipid_413695850_plot.png]

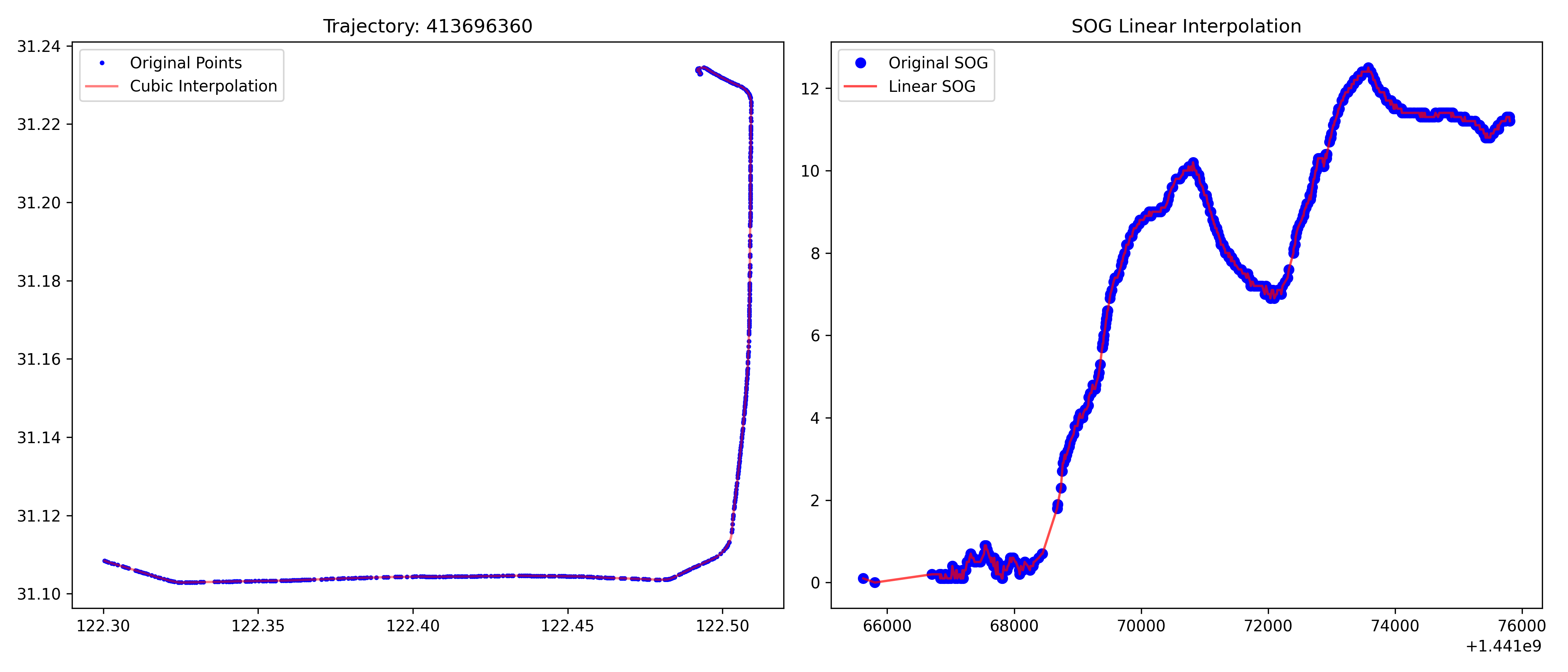

Supplement: S1 File — (ZIP) [file pone.0342781.s001.zip › data/interpolation/shipid_413696360_plot.png]

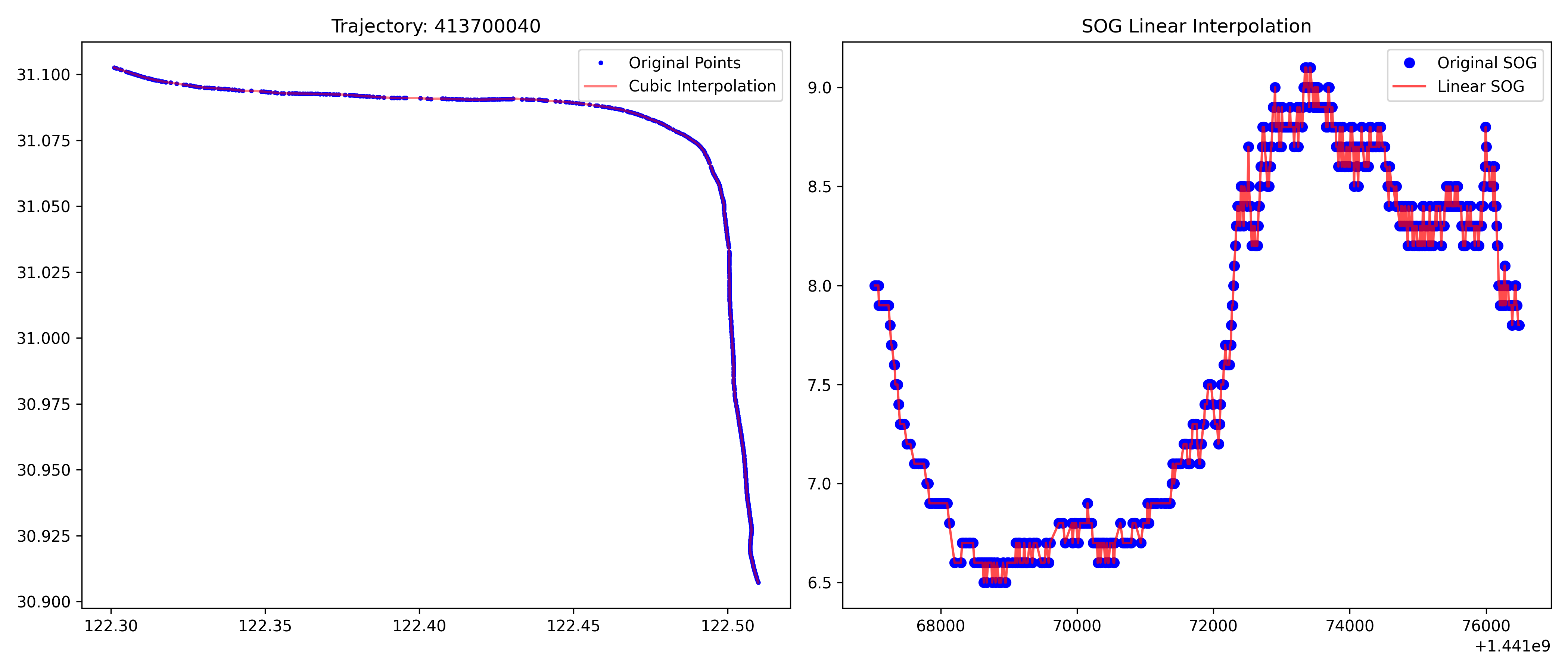

Supplement: S1 File — (ZIP) [file pone.0342781.s001.zip › data/interpolation/shipid_413700040_plot.png]

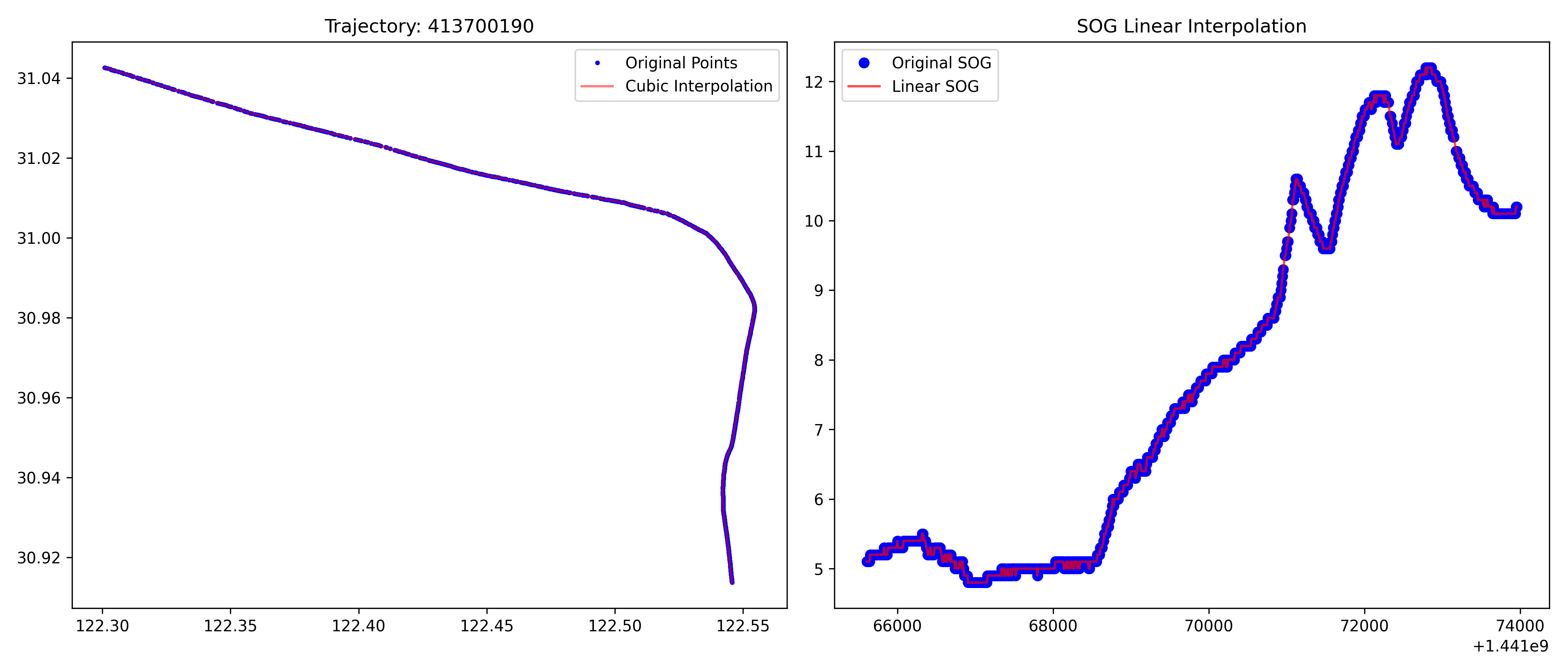

Supplement: S1 File — (ZIP) [file pone.0342781.s001.zip › data/interpolation/shipid_413700190_plot.png]

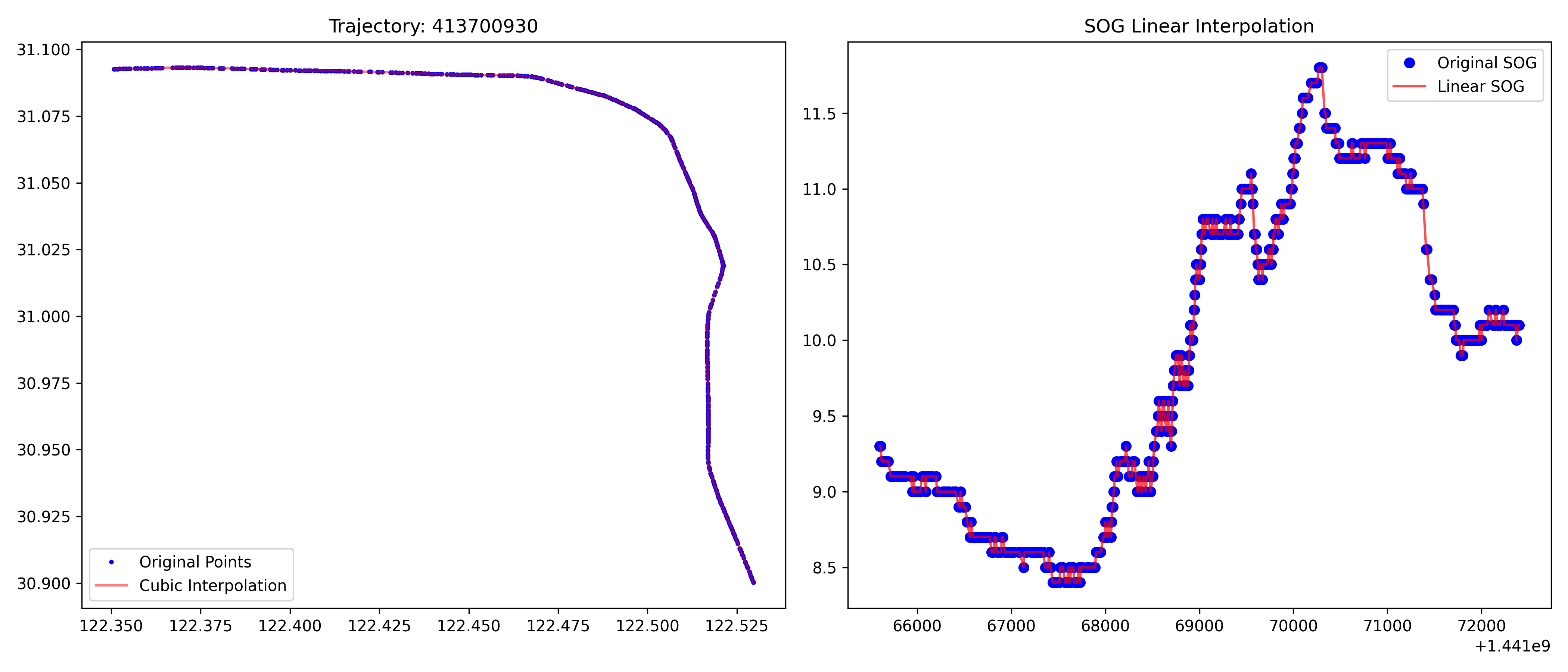

Supplement: S1 File — (ZIP) [file pone.0342781.s001.zip › data/interpolation/shipid_413700930_plot.png]

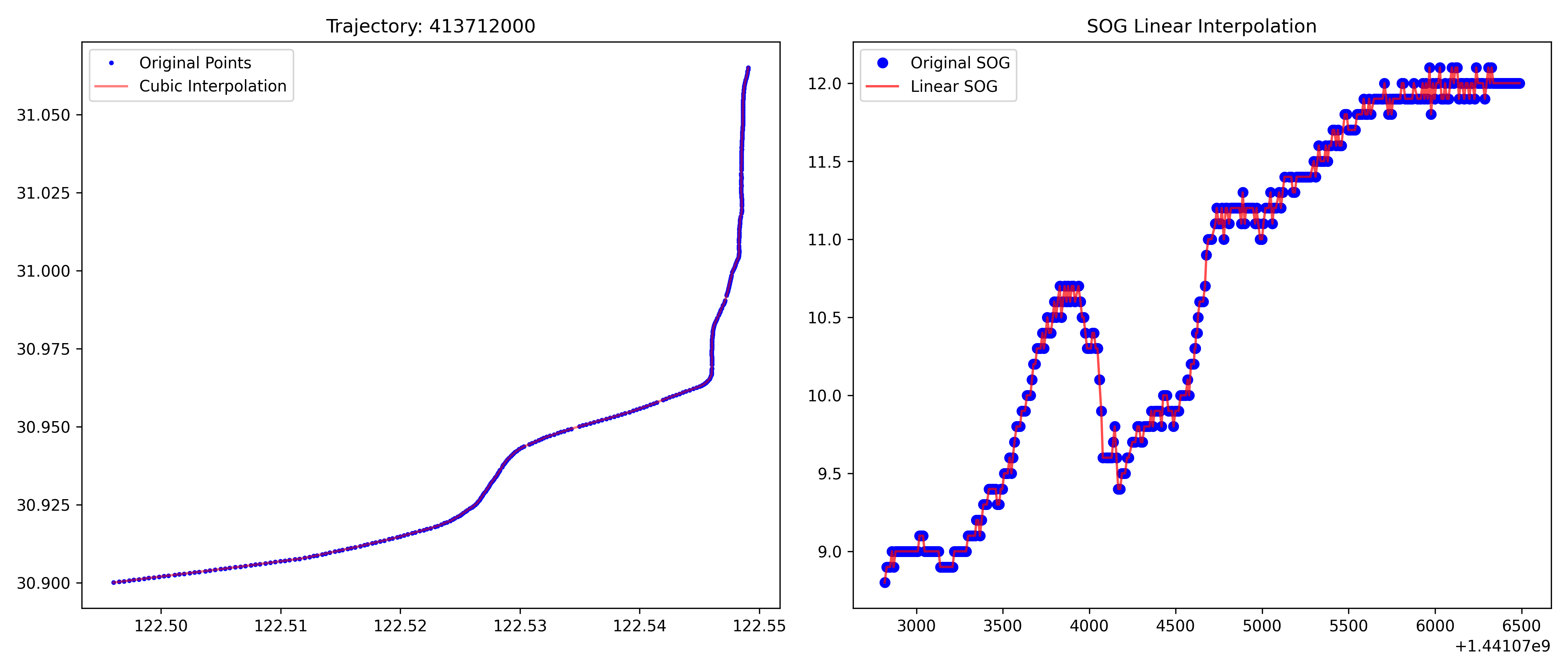

Supplement: S1 File — (ZIP) [file pone.0342781.s001.zip › data/interpolation/shipid_413712000_plot.png]

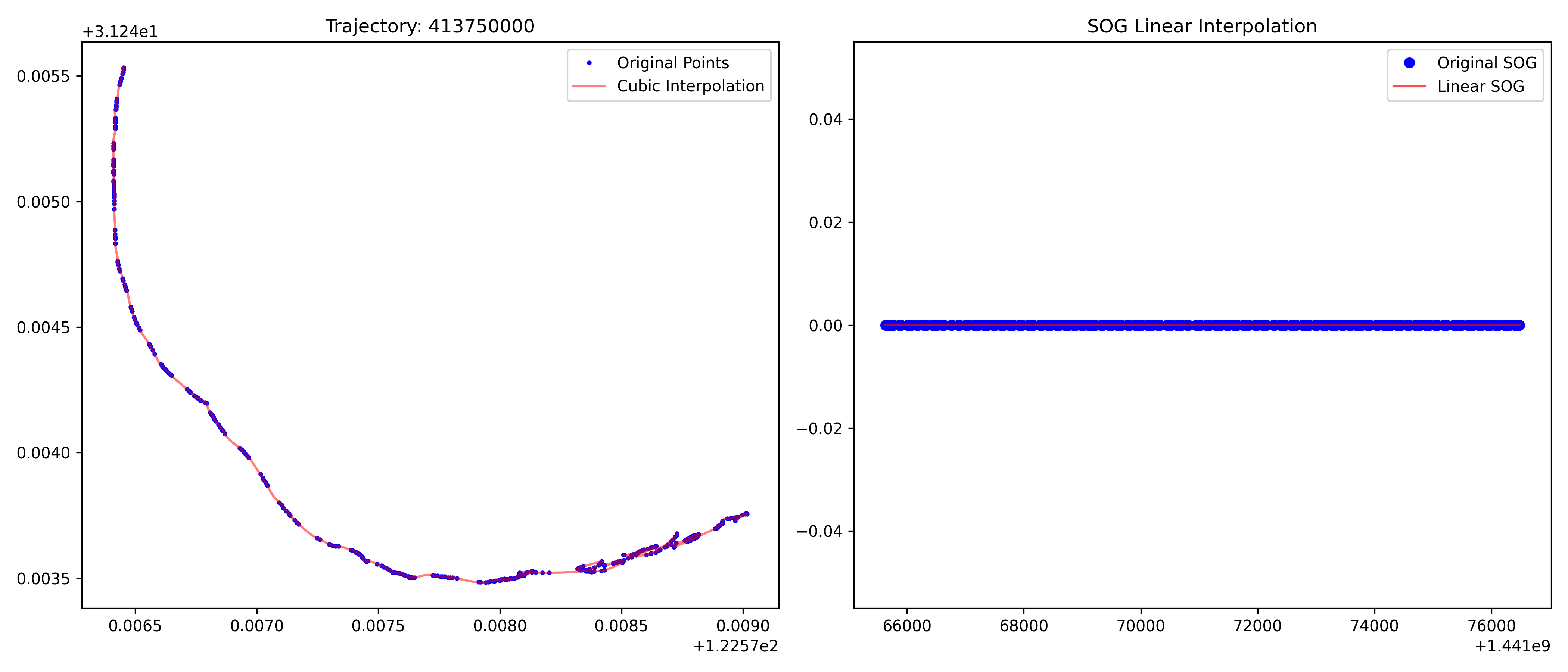

Supplement: S1 File — (ZIP) [file pone.0342781.s001.zip › data/interpolation/shipid_413750000_plot.png]

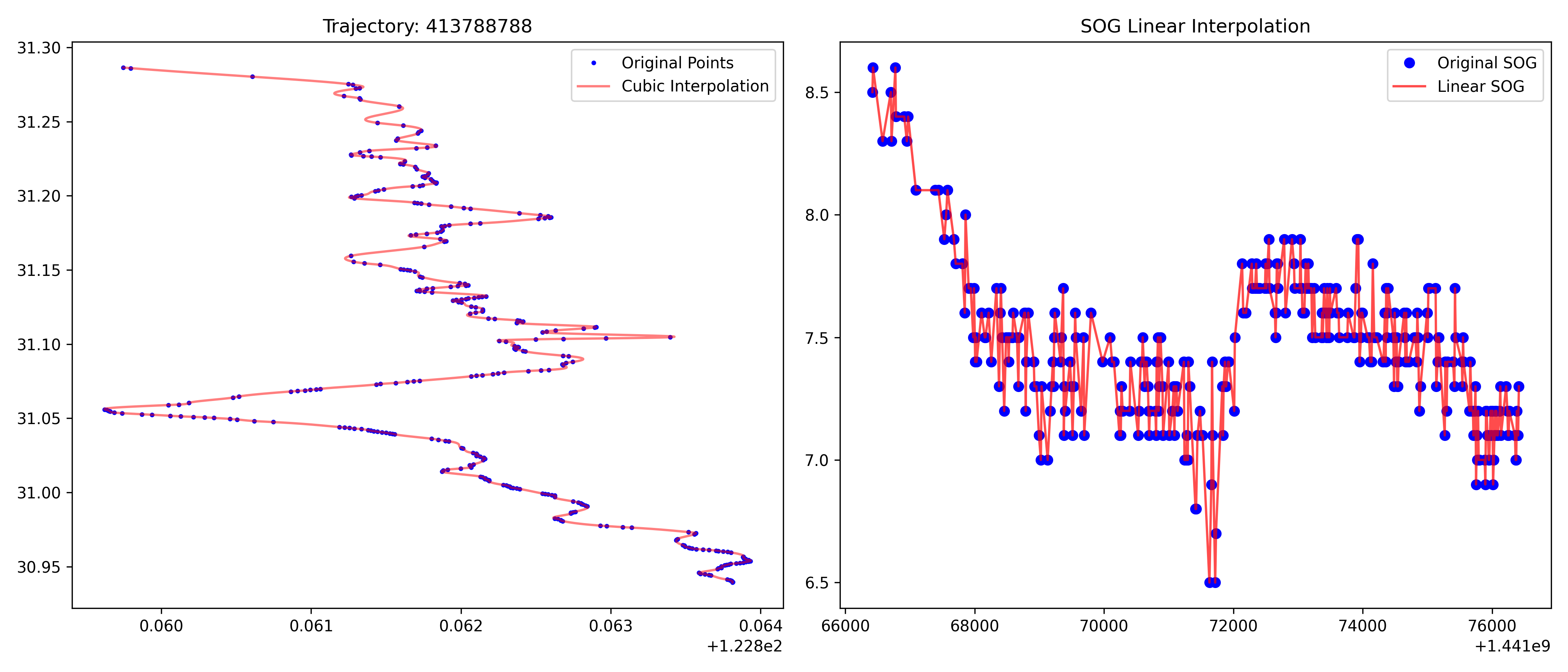

Supplement: S1 File — (ZIP) [file pone.0342781.s001.zip › data/interpolation/shipid_413788788_plot.png]

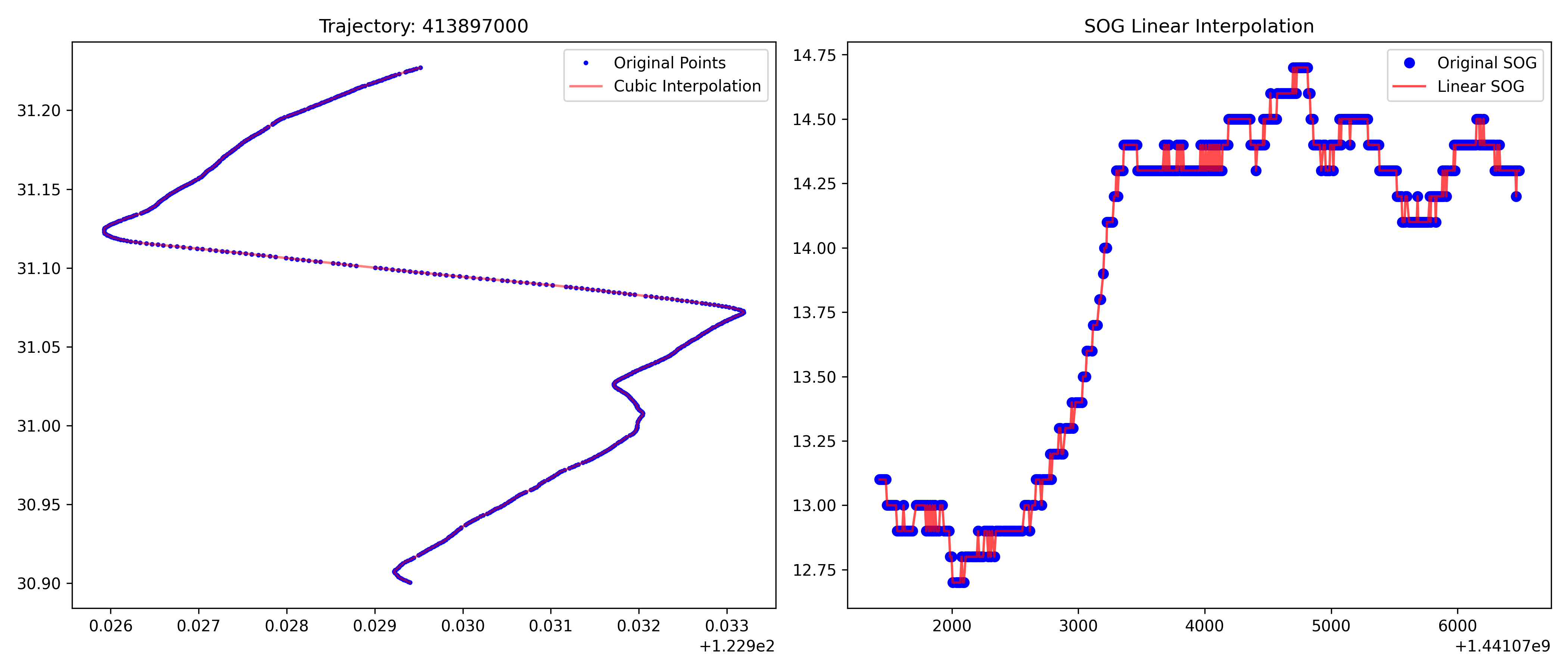

Supplement: S1 File — (ZIP) [file pone.0342781.s001.zip › data/interpolation/shipid_413897000_plot.png]

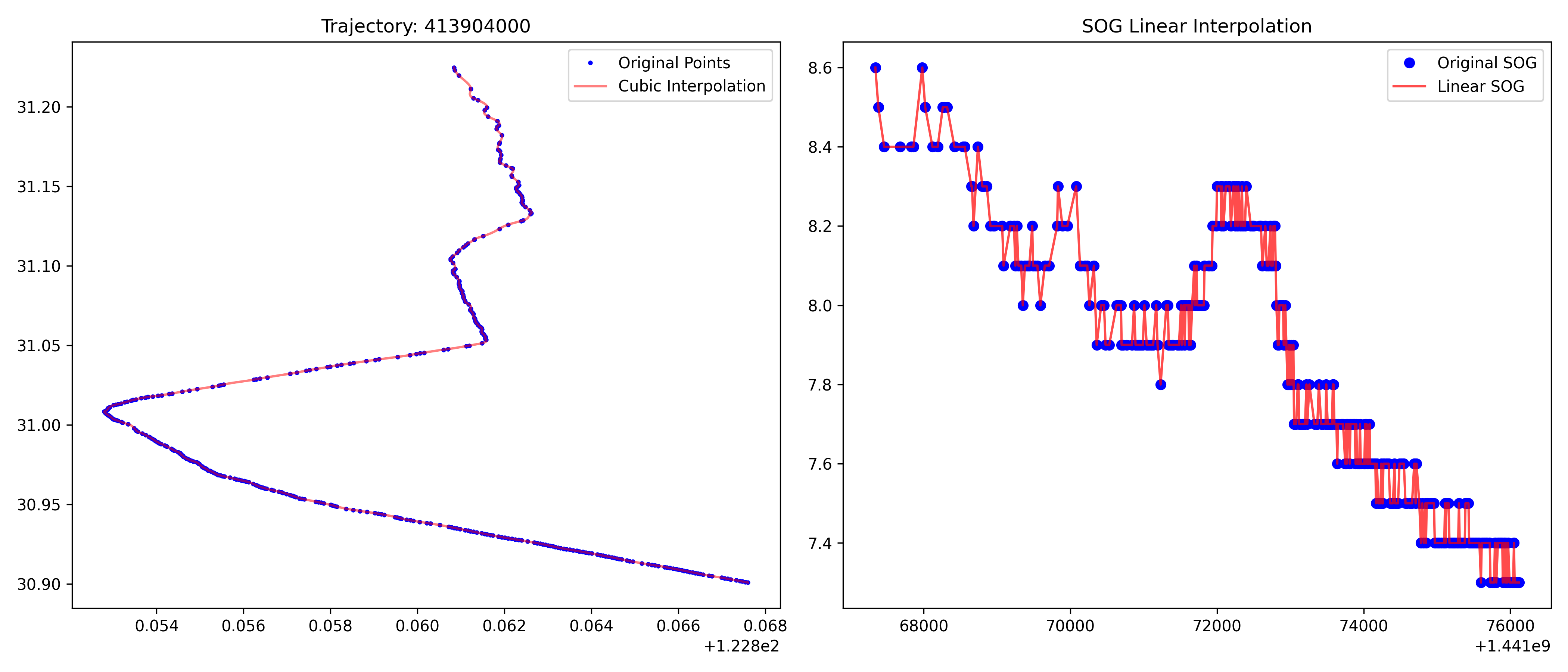

Supplement: S1 File — (ZIP) [file pone.0342781.s001.zip › data/interpolation/shipid_413904000_plot.png]

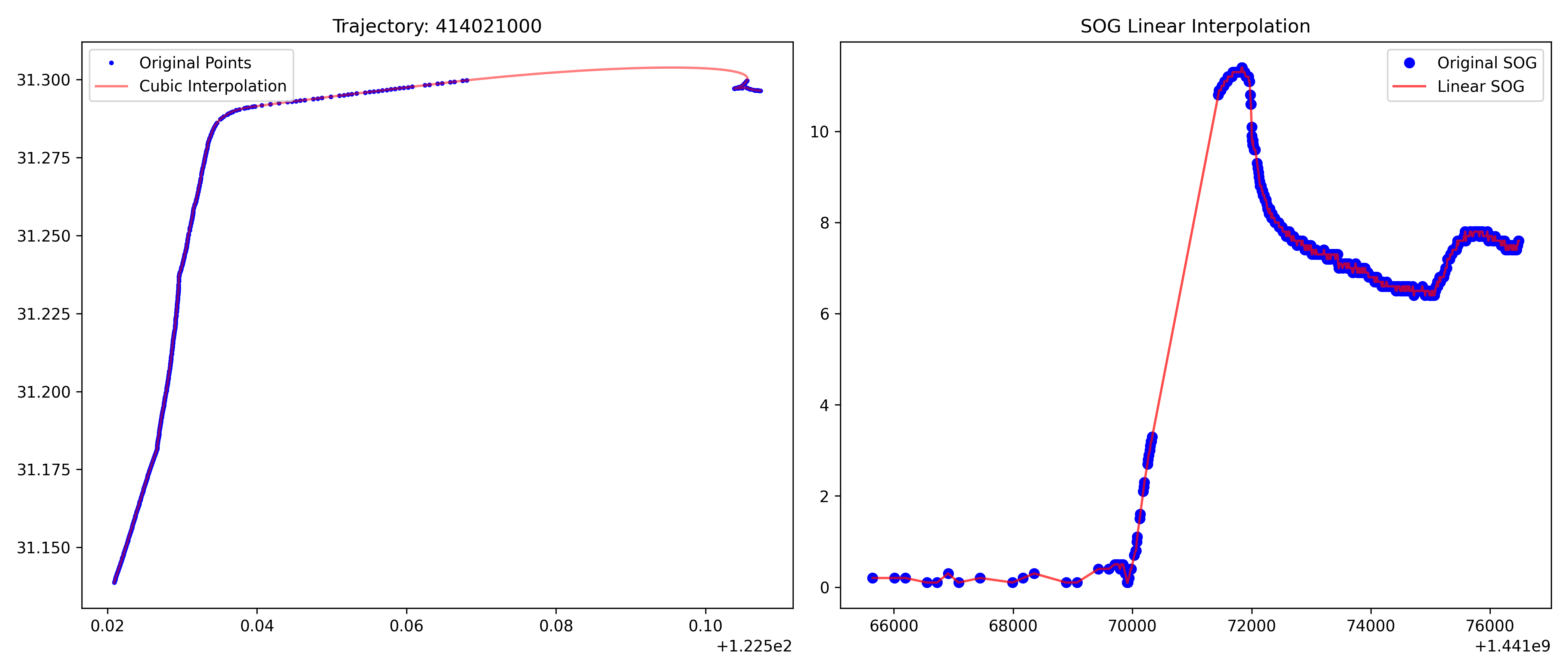

Supplement: S1 File — (ZIP) [file pone.0342781.s001.zip › data/interpolation/shipid_414021000_plot.png]

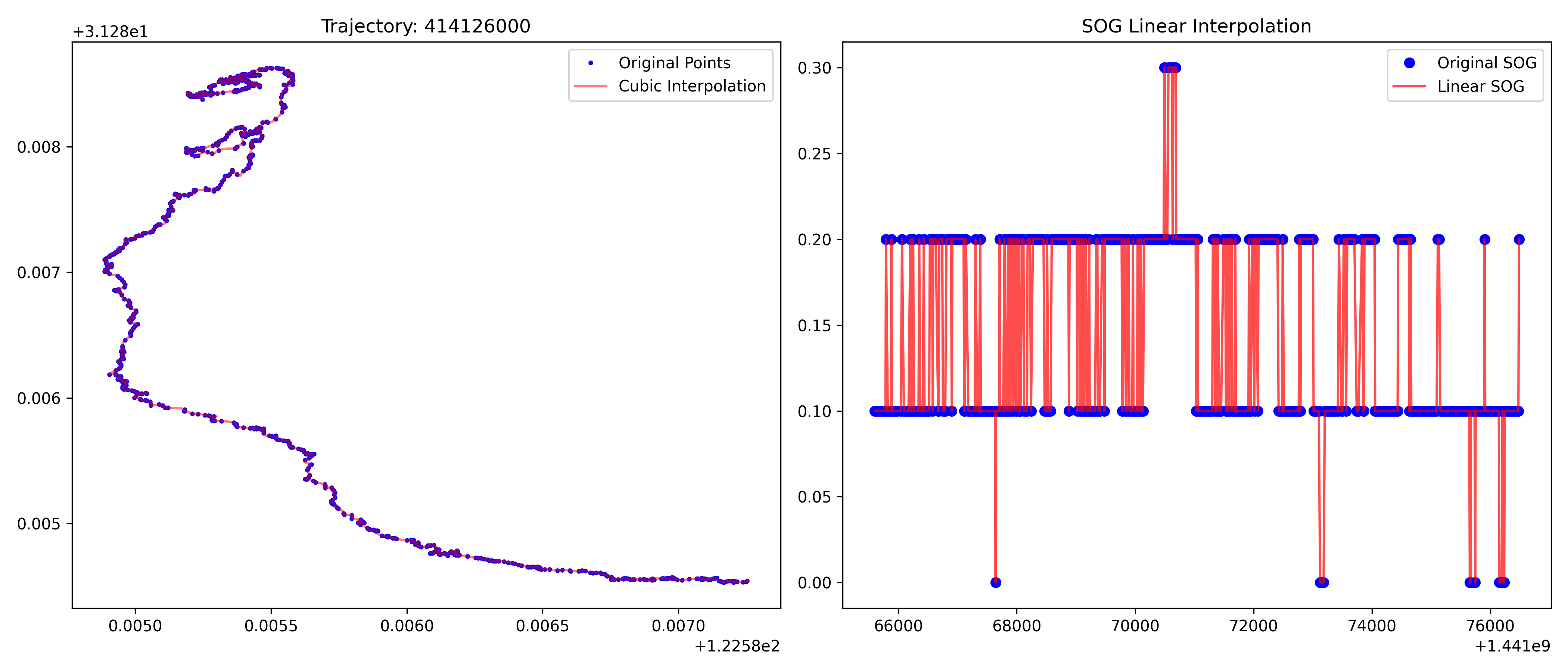

Supplement: S1 File — (ZIP) [file pone.0342781.s001.zip › data/interpolation/shipid_414126000_plot.png]

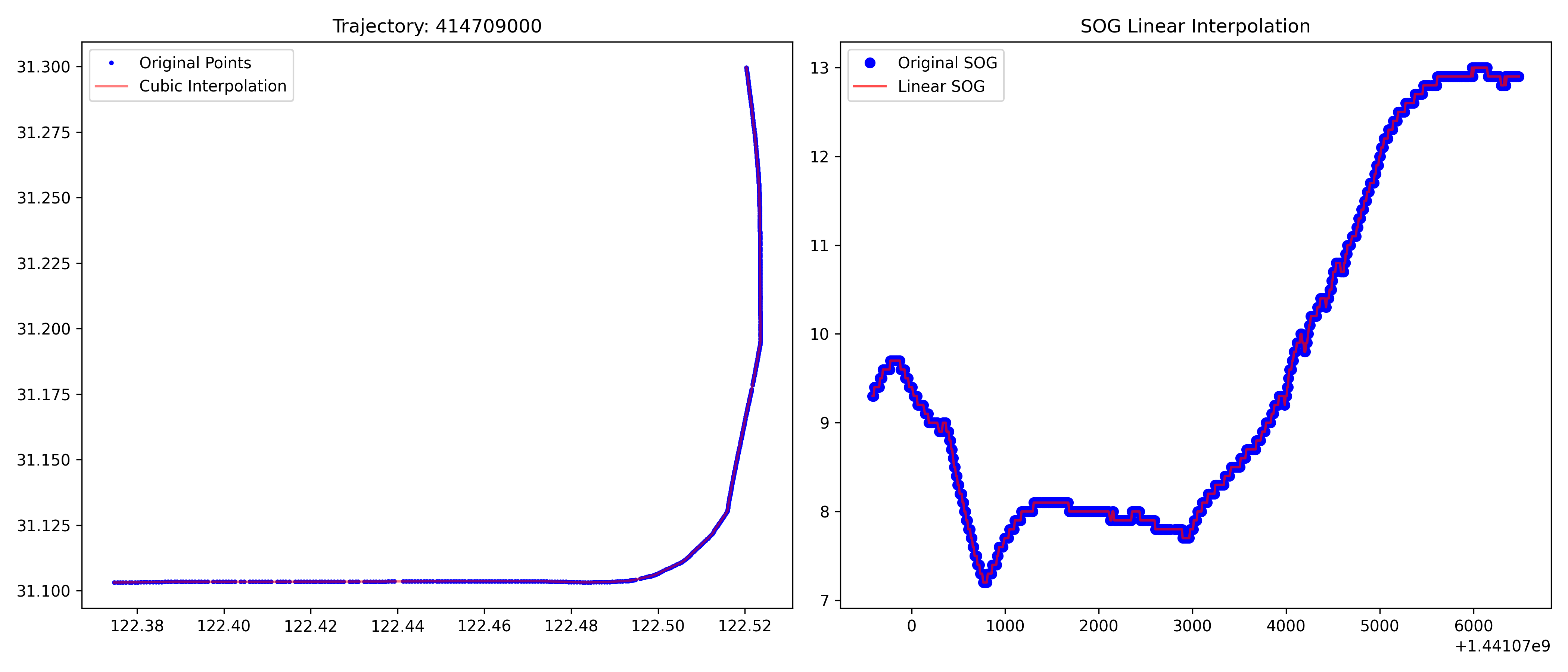

Supplement: S1 File — (ZIP) [file pone.0342781.s001.zip › data/interpolation/shipid_414709000_plot.png]

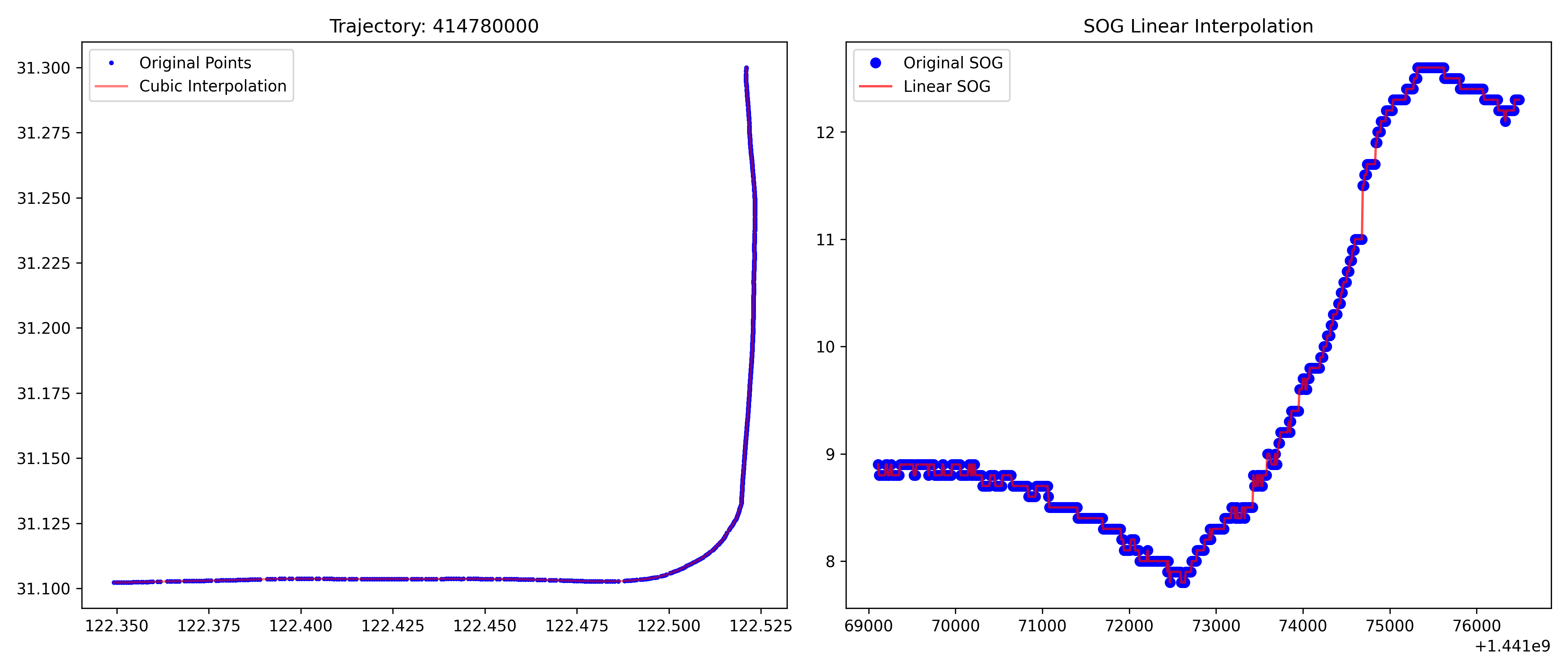

Supplement: S1 File — (ZIP) [file pone.0342781.s001.zip › data/interpolation/shipid_414780000_plot.png]

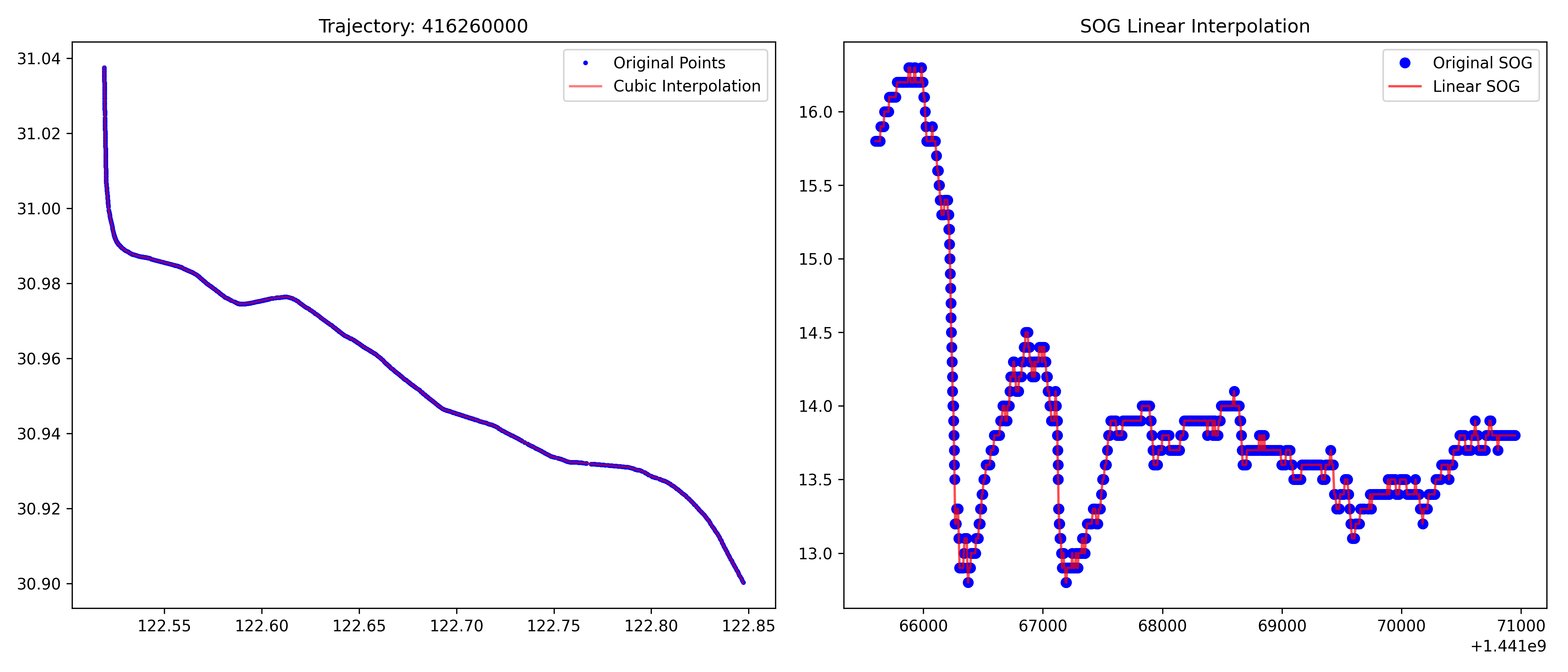

Supplement: S1 File — (ZIP) [file pone.0342781.s001.zip › data/interpolation/shipid_416260000_plot.png]

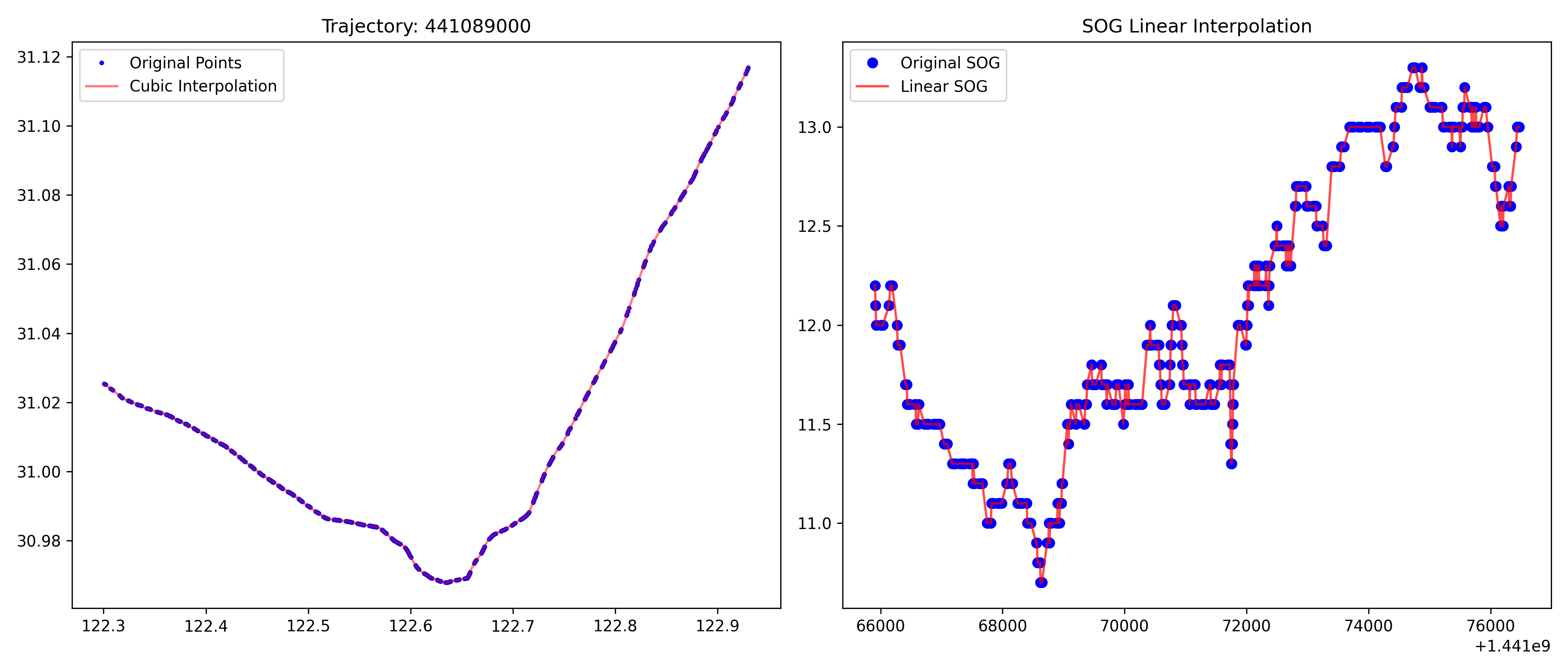

Supplement: S1 File — (ZIP) [file pone.0342781.s001.zip › data/interpolation/shipid_441089000_plot.png]

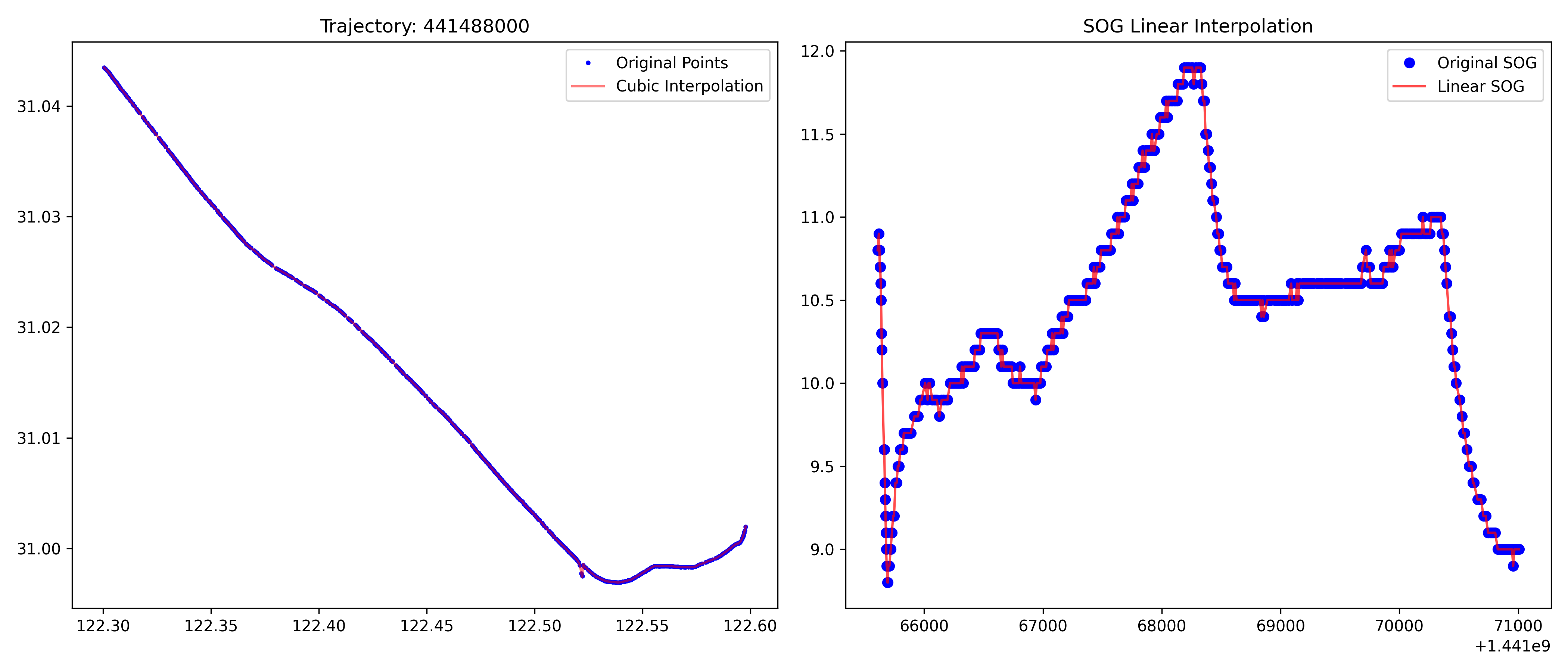

Supplement: S1 File — (ZIP) [file pone.0342781.s001.zip › data/interpolation/shipid_441488000_plot.png]

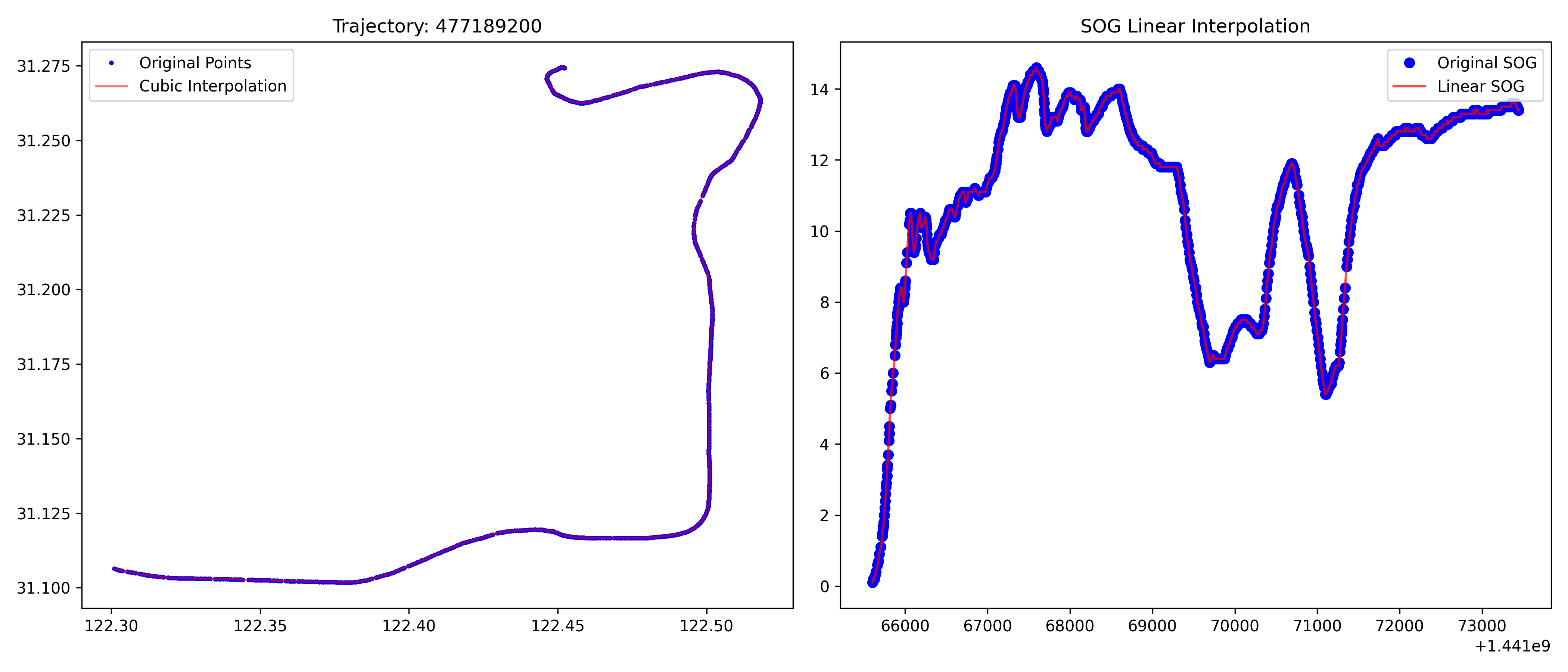

Supplement: S1 File — (ZIP) [file pone.0342781.s001.zip › data/interpolation/shipid_477189200_plot.png]

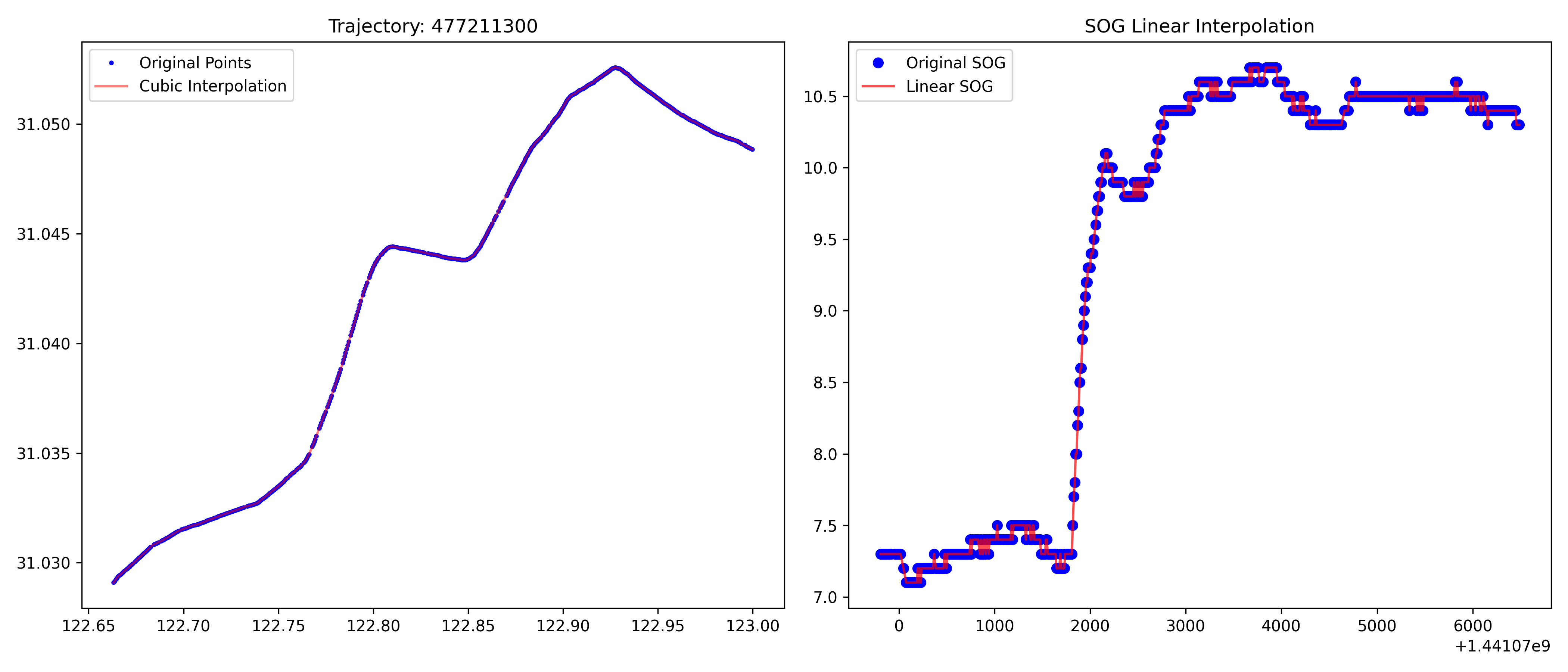

Supplement: S1 File — (ZIP) [file pone.0342781.s001.zip › data/interpolation/shipid_477211300_plot.png]

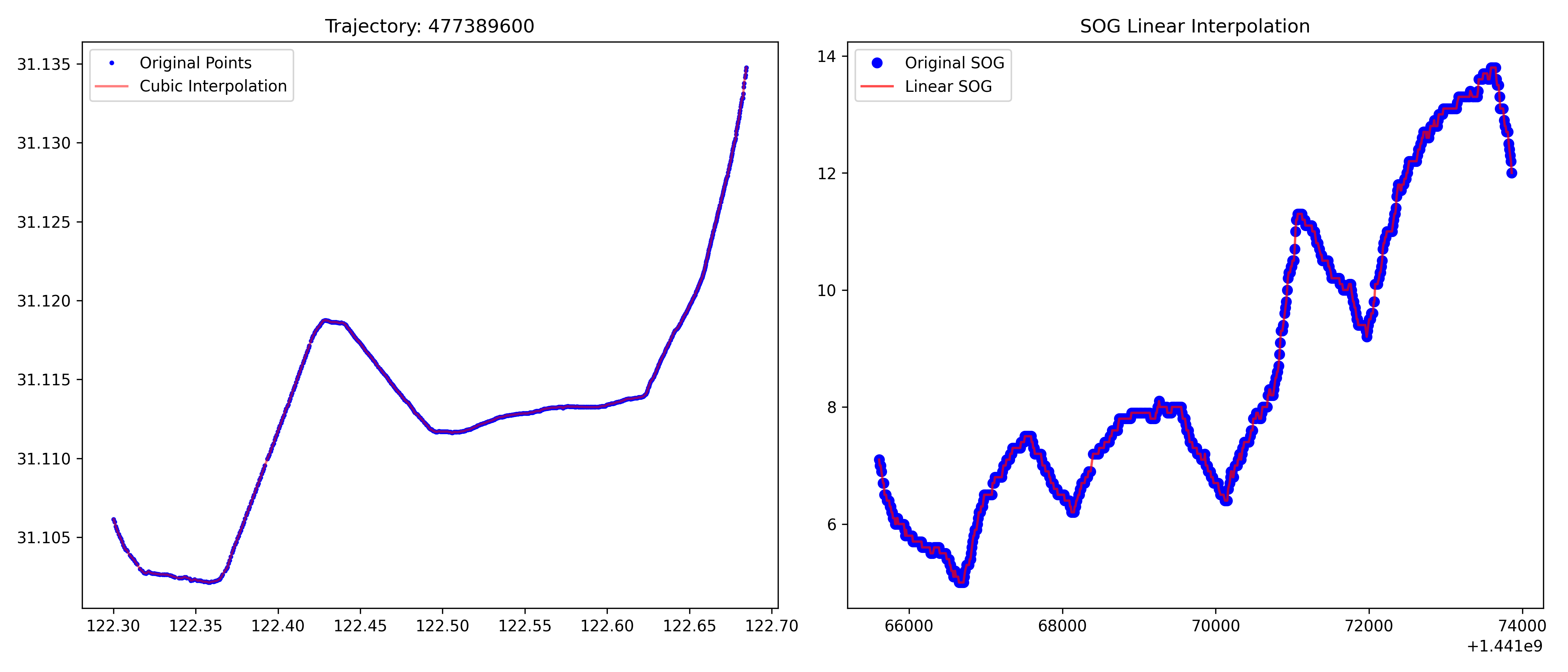

Supplement: S1 File — (ZIP) [file pone.0342781.s001.zip › data/interpolation/shipid_477389600_plot.png]

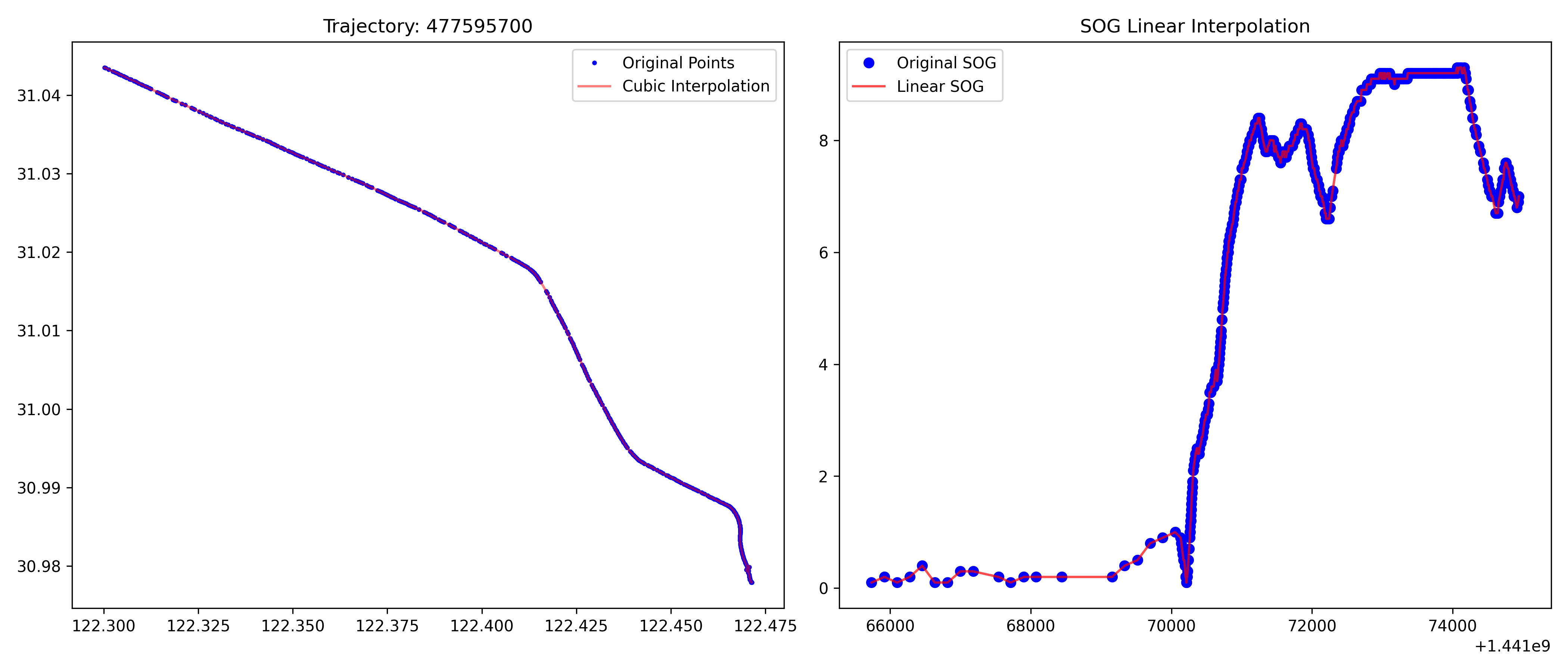

Supplement: S1 File — (ZIP) [file pone.0342781.s001.zip › data/interpolation/shipid_477595700_plot.png]

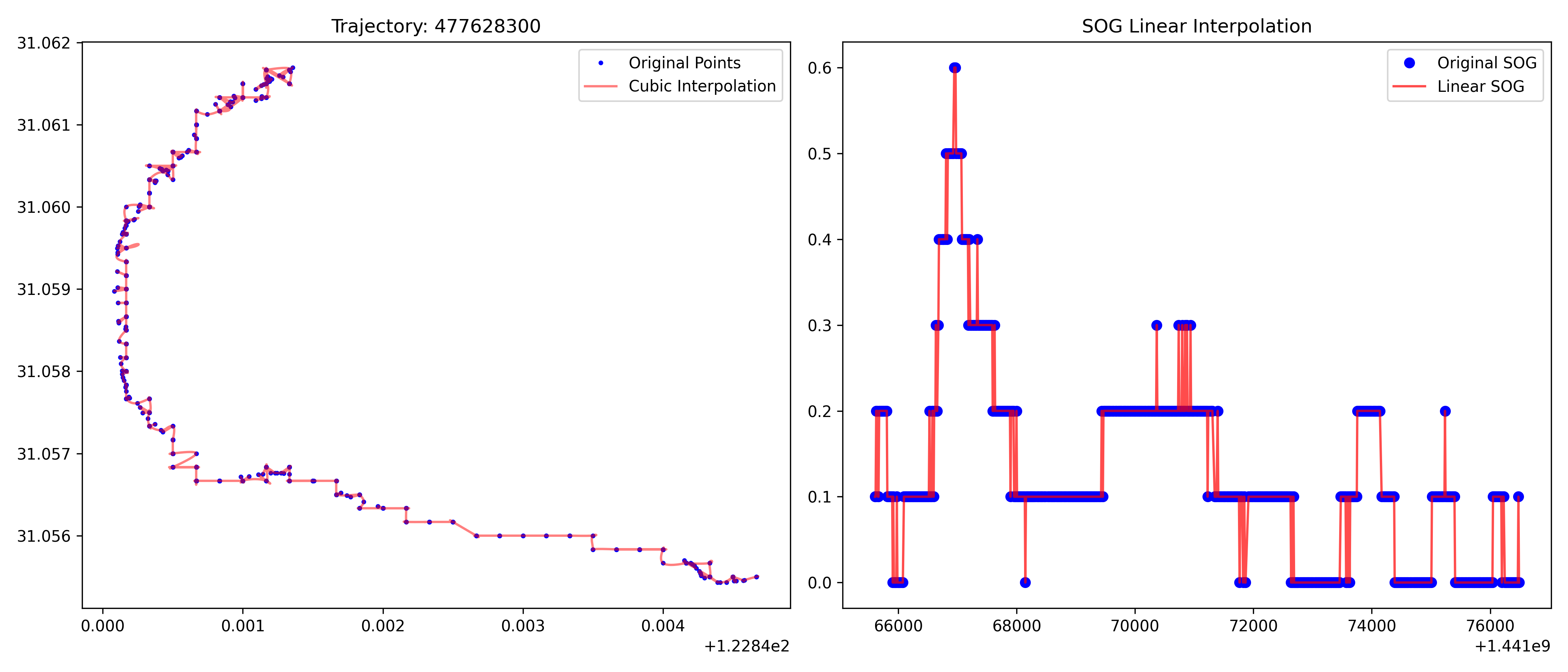

Supplement: S1 File — (ZIP) [file pone.0342781.s001.zip › data/interpolation/shipid_477628300_plot.png]

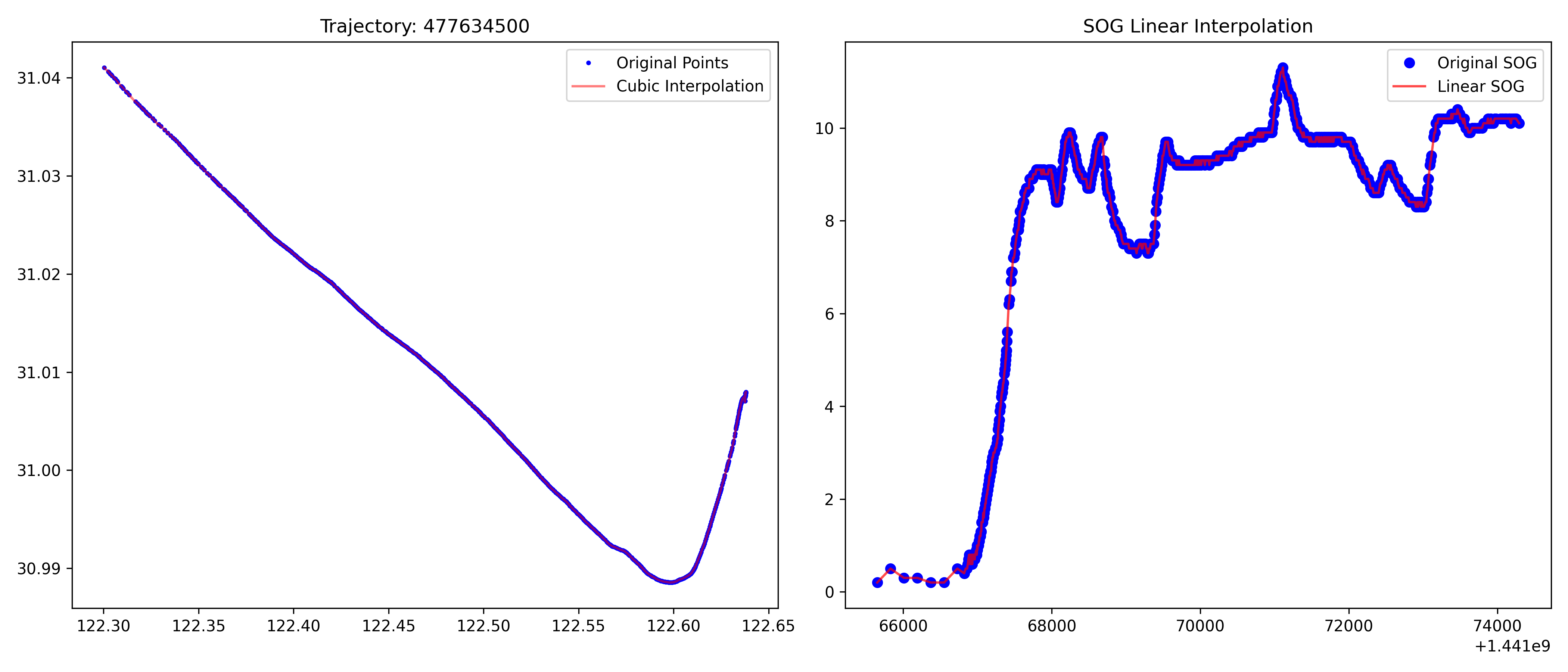

Supplement: S1 File — (ZIP) [file pone.0342781.s001.zip › data/interpolation/shipid_477634500_plot.png]

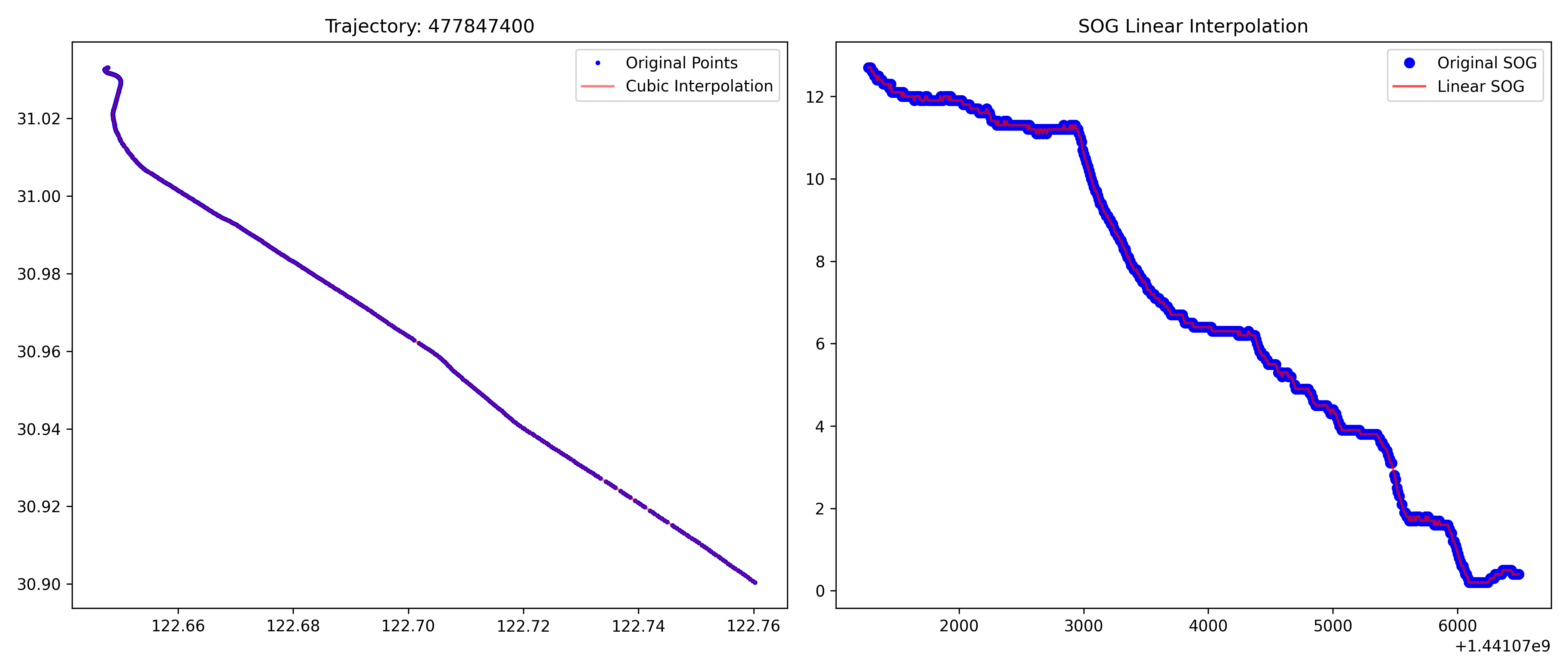

Supplement: S1 File — (ZIP) [file pone.0342781.s001.zip › data/interpolation/shipid_477847400_plot.png]

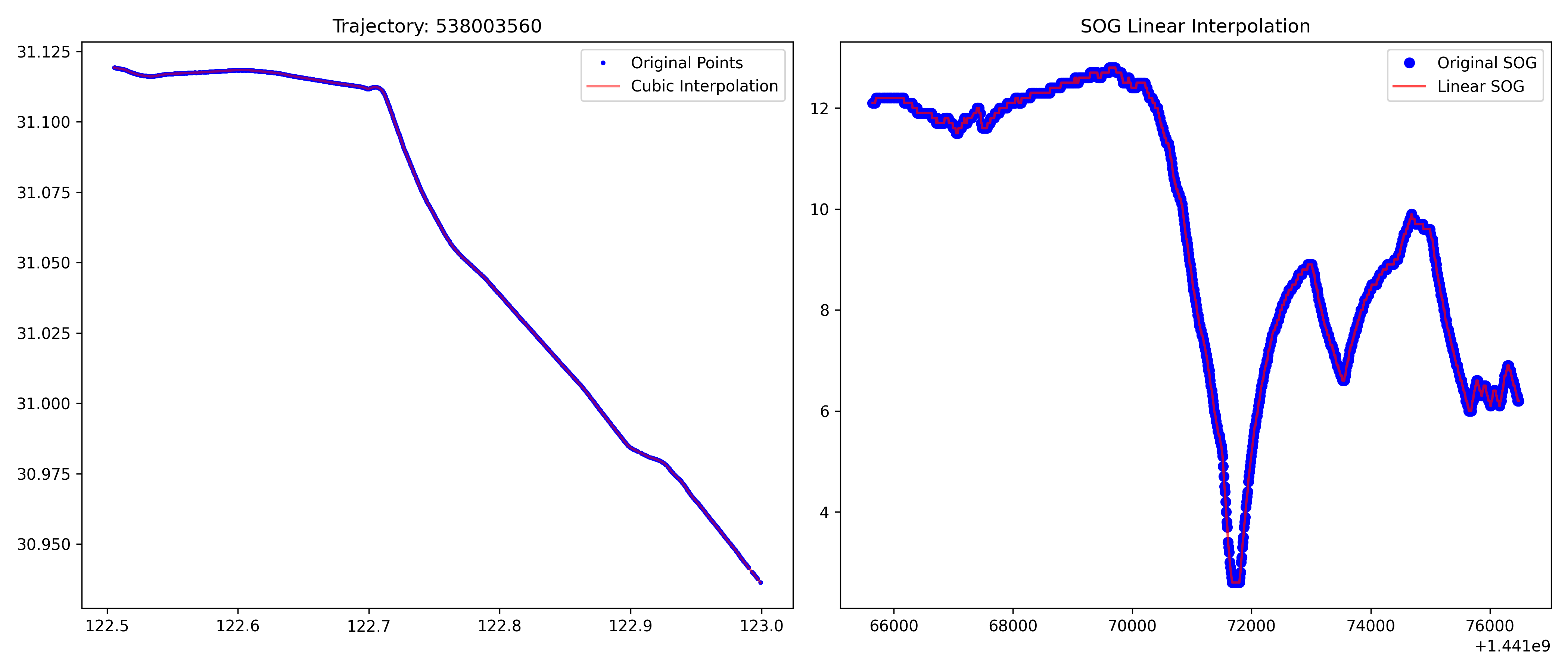

Supplement: S1 File — (ZIP) [file pone.0342781.s001.zip › data/interpolation/shipid_538003560_plot.png]

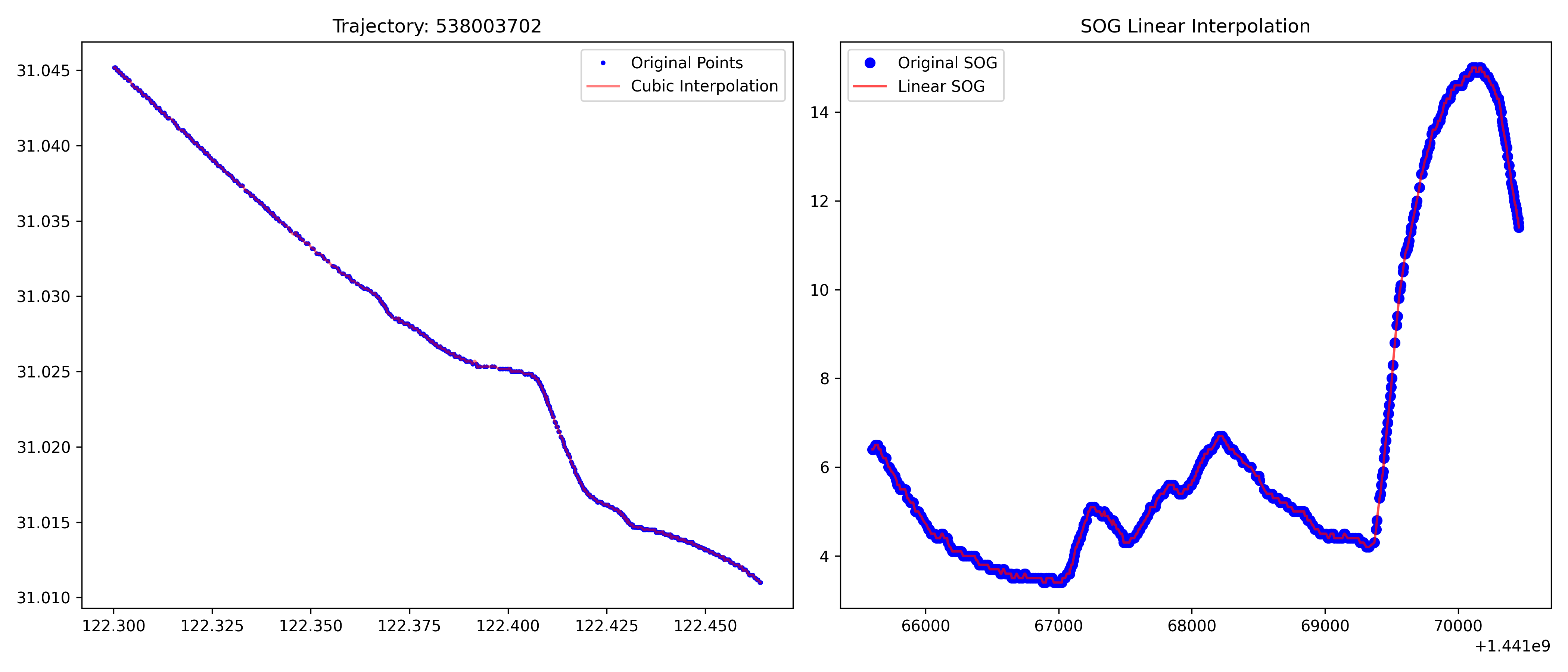

Supplement: S1 File — (ZIP) [file pone.0342781.s001.zip › data/interpolation/shipid_538003702_plot.png]

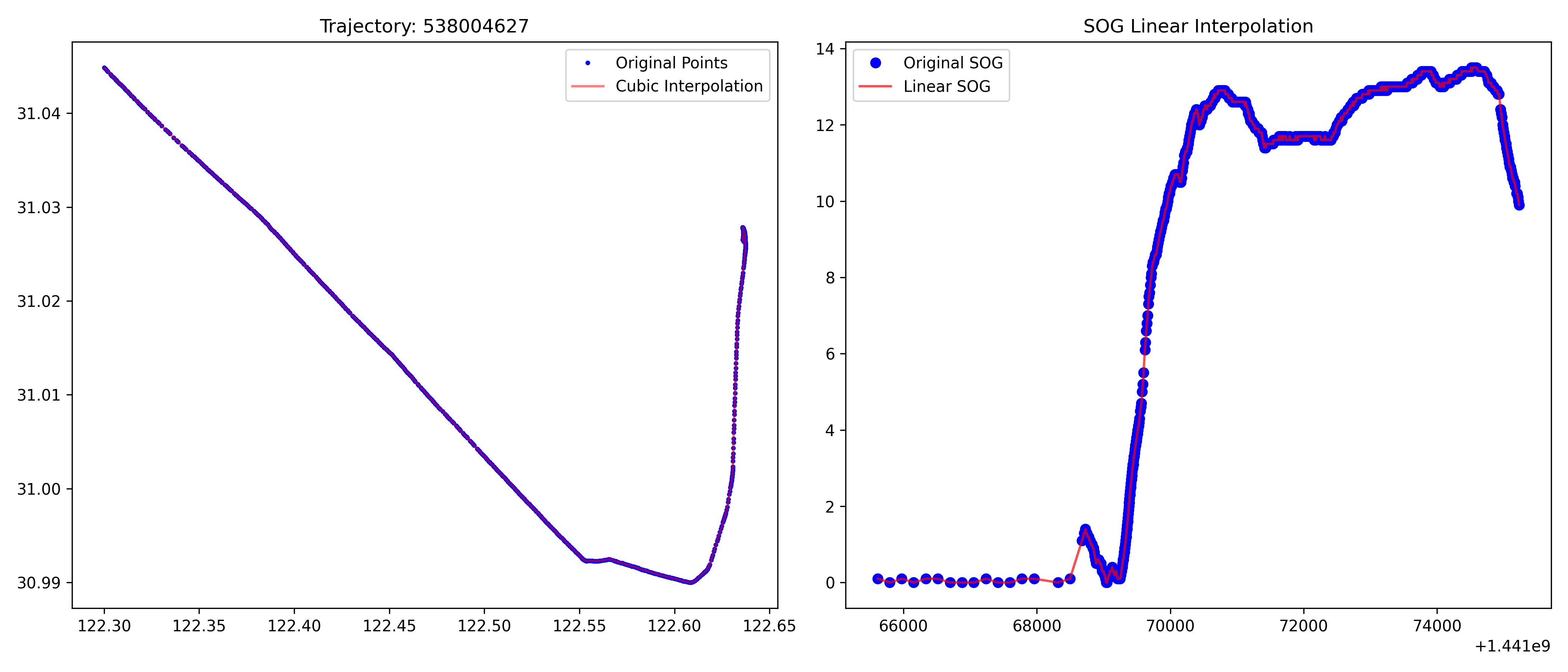

Supplement: S1 File — (ZIP) [file pone.0342781.s001.zip › data/interpolation/shipid_538004627_plot.png]

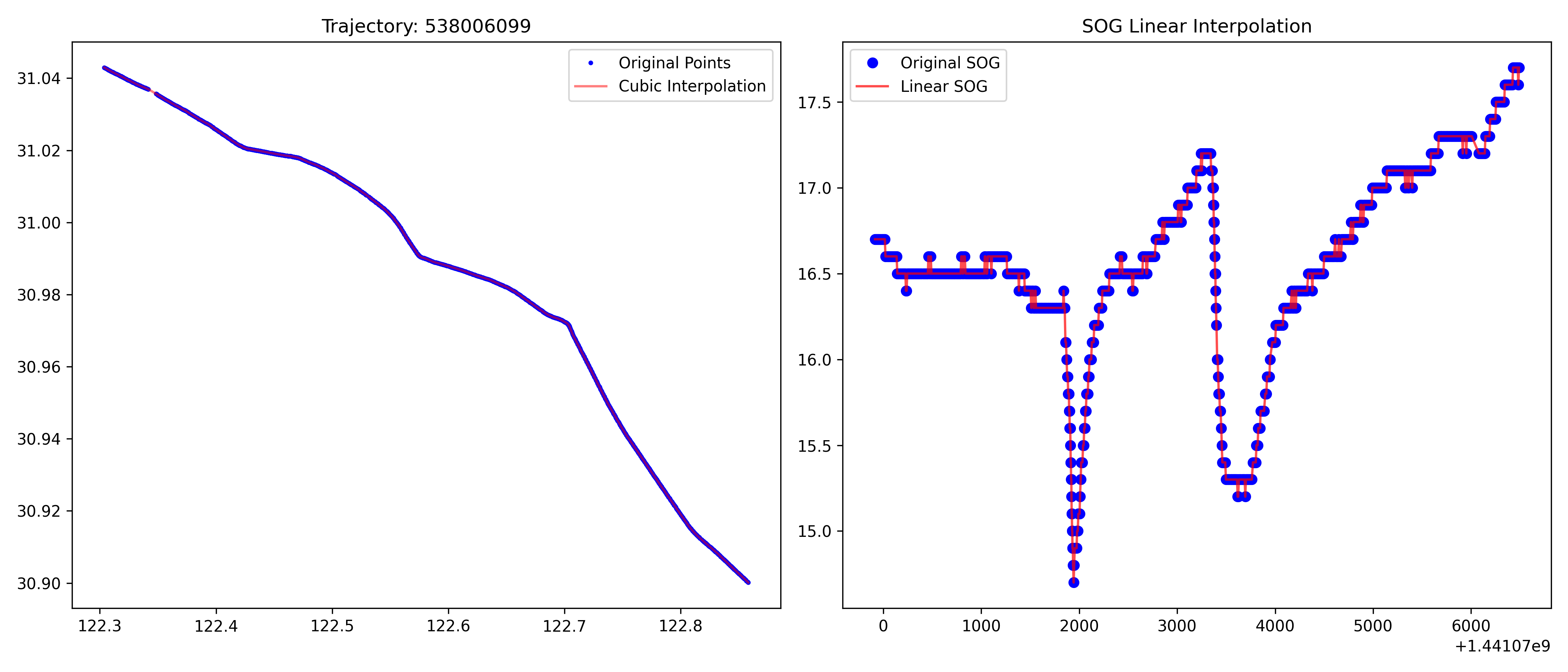

Supplement: S1 File — (ZIP) [file pone.0342781.s001.zip › data/interpolation/shipid_538006099_plot.png]

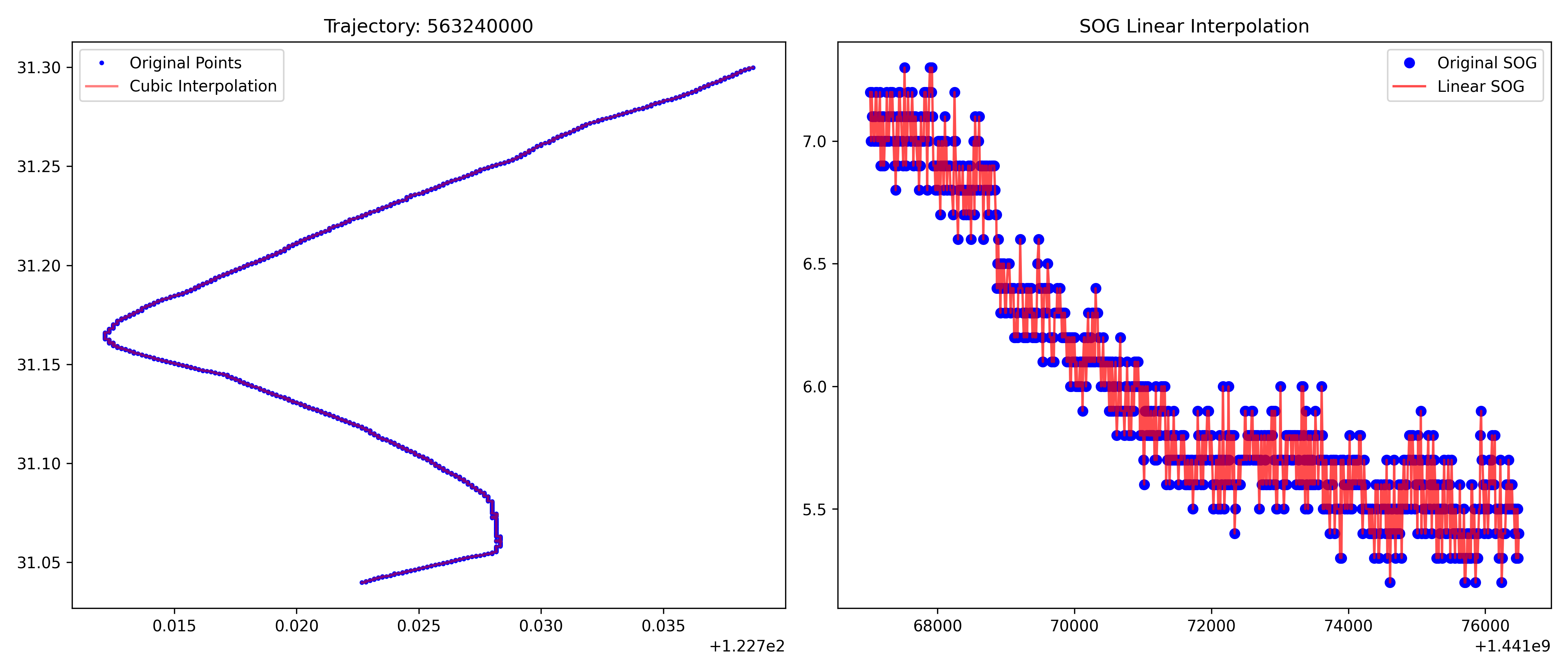

Supplement: S1 File — (ZIP) [file pone.0342781.s001.zip › data/interpolation/shipid_563240000_plot.png]

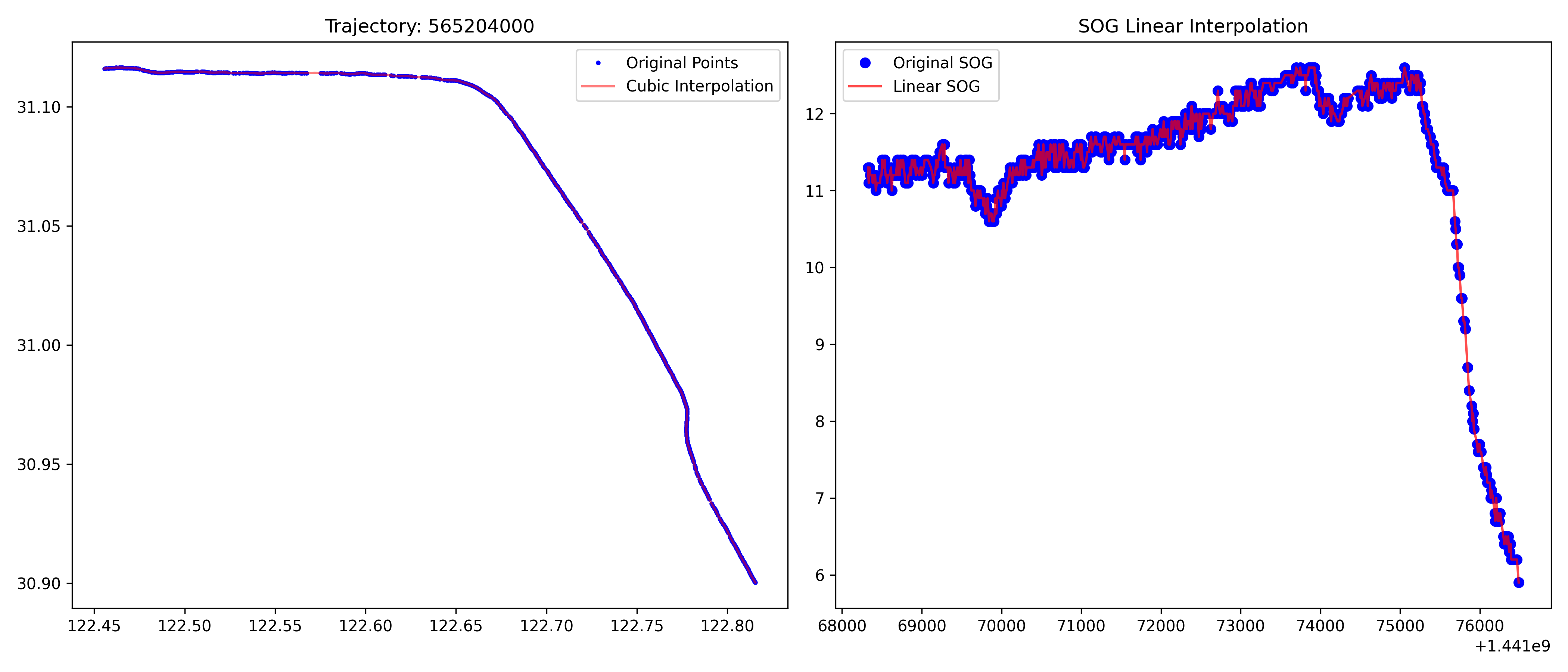

Supplement: S1 File — (ZIP) [file pone.0342781.s001.zip › data/interpolation/shipid_565204000_plot.png]

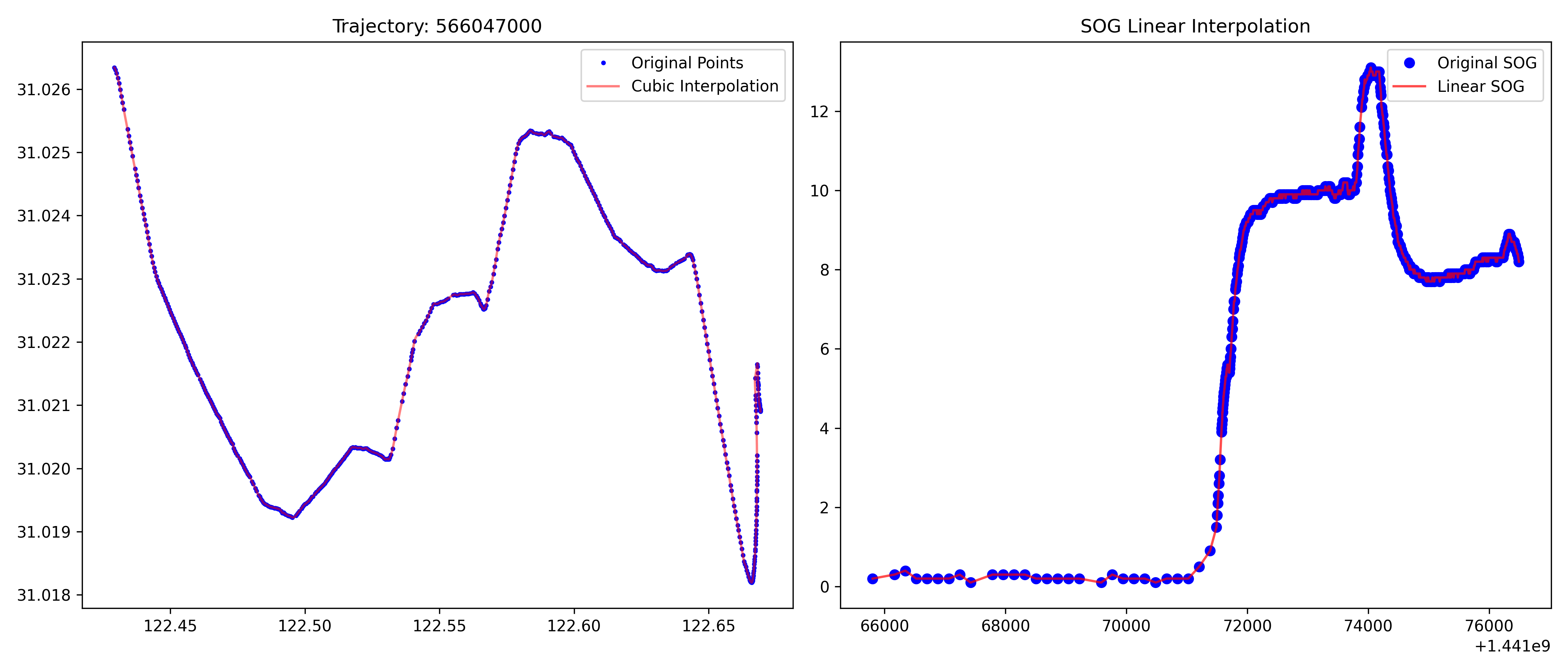

Supplement: S1 File — (ZIP) [file pone.0342781.s001.zip › data/interpolation/shipid_566047000_plot.png]

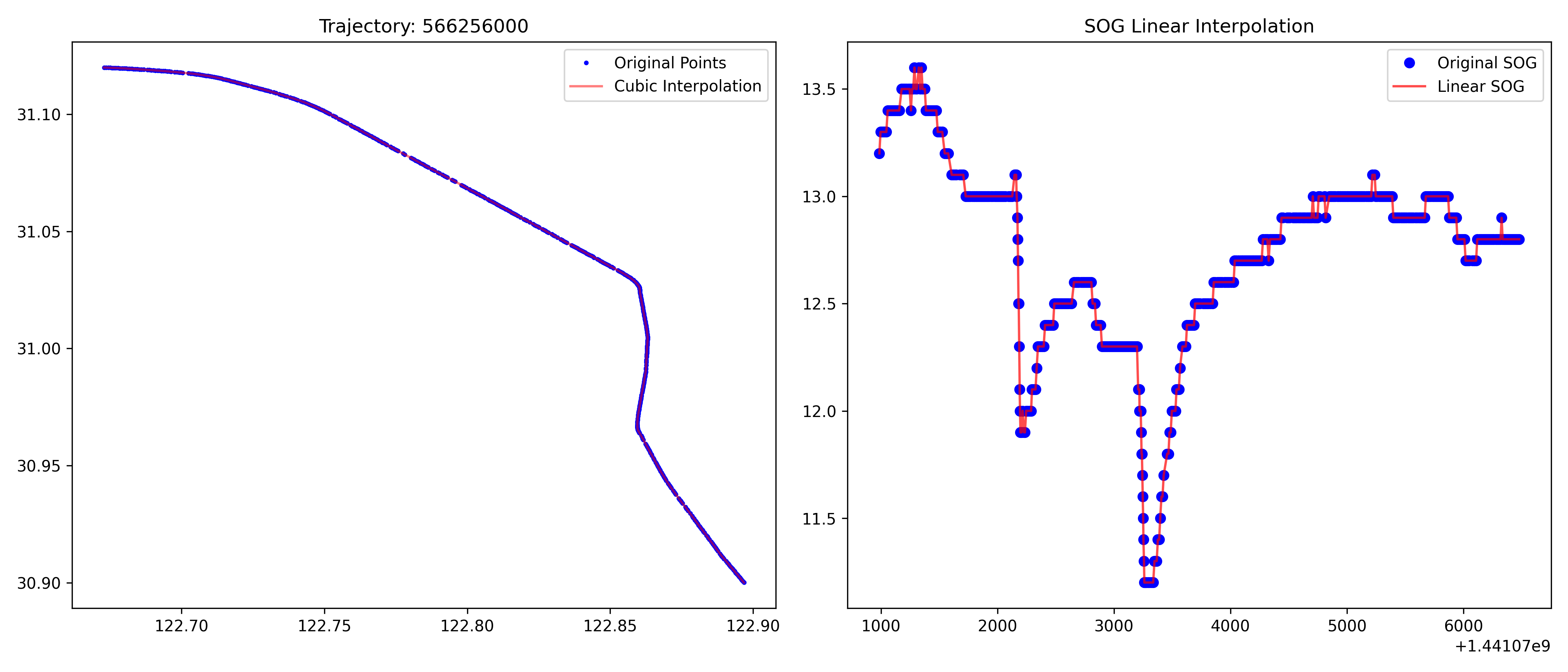

Supplement: S1 File — (ZIP) [file pone.0342781.s001.zip › data/interpolation/shipid_566256000_plot.png]
